# High Accuracy Classification of Developmental Toxicants by In Vitro Tests of Human Neuroepithelial and Cardiomyoblast Differentiation

**DOI:** 10.3390/cells11213404

**Published:** 2022-10-27

**Authors:** Florian Seidel, Anna Cherianidou, Franziska Kappenberg, Miriam Marta, Nadine Dreser, Jonathan Blum, Tanja Waldmann, Nils Blüthgen, Johannes Meisig, Katrin Madjar, Margit Henry, Tamara Rotshteyn, Andreas Scholtz-Illigens, Rosemarie Marchan, Karolina Edlund, Marcel Leist, Jörg Rahnenführer, Agapios Sachinidis, Jan Georg Hengstler

**Affiliations:** 1 Leibniz Research Centre for Working Environment and Human Factors (IfADo), Technical University of Dortmund, Ardeystrasse 67, 44139 Dortmund, Germany; 2 Working Group Sachinidis, Center for Physiology, Faculty of Medicine and University Hospital Cologne, University of Cologne, Robert-Koch-Str. 39, 50931 Cologne, Germany; 3 Department of Statistics, TU Dortmund University, Vogelpothsweg 87, 44227 Dortmund, Germany; 4 In Vitro Toxicology and Biomedicine, Department of Biology, University of Konstanz, Universitätsstr. 10, 78454 Konstanz, Germany; 5 Department of Advanced Cell Systems, trenzyme GmbH, Byk-Gulden-Str. 2, 78467 Konstanz, Germany; 6 Institute of Pathology, Charité-Universitätsmedizin Berlin, Chariteplatz 1, 10117 Berlin, Germany; 7 IRI Life Sciences, Humboldt Universität zu Berlin, Philippstraße 13, Haus 18, 10115 Berlin, Germany; 8 Center for Molecular Medicine Cologne (CMMC), University of Cologne, 50931 Cologne, Germany

**Keywords:** alternative testing strategies, in vitro test, induced pluripotent stem cells, developmental and reproductive toxicity, drug screening, toxicogenomics, transcriptomics, gene expression

## Abstract

Human-relevant tests to predict developmental toxicity are urgently needed. A currently intensively studied approach makes use of differentiating human stem cells to measure chemically-induced deviations of the normal developmental program, as in a recent study based on cardiac differentiation (UKK2). Here, we (i) tested the performance of an assay modeling neuroepithelial differentiation (UKN1), and (ii) explored the benefit of combining assays (UKN1 and UKK2) that model different germ layers. Substance-induced cytotoxicity and genome-wide expression profiles of 23 teratogens and 16 non-teratogens at human-relevant concentrations were generated and used for statistical classification, resulting in accuracies of the UKN1 assay of 87–90%. A comparison to the UKK2 assay (accuracies of 90–92%) showed, in general, a high congruence in compound classification that may be explained by the fact that there was a high overlap of signaling pathways. Finally, the combination of both assays improved the prediction compared to each test alone, and reached accuracies of 92–95%. Although some compounds were misclassified by the individual tests, we conclude that UKN1 and UKK2 can be used for a reliable detection of teratogens in vitro, and that a combined analysis of tests that differentiate hiPSCs into different germ layers and cell types can even further improve the prediction of developmental toxicants.

## 1. Introduction

Testing for developmental toxicity in vivo, for example, by two-generation reproduction studies, is cost-intensive and requires large numbers of experimental animals [1,2]. Therefore, a significant advancement in this field would be if developmental toxicity could reliably be identified in vitro. Recently, much effort has been invested into establishing in vitro tests using pluripotent stem cells [3,4], zebrafish [5], or neuronal cells [6,7]. Moreover, test compound-induced changes in amino acid concentrations in the culture medium of human embryonic stem cells have been used as a readout [8,9]. This test identified developmental toxicants with an accuracy, sensitivity, and specificity of 77, 57, and 100%, respectively. In our previous studies, we focused on gene expression changes induced by teratogens in human-induced pluripotent stem cells (hiPSC) and observed that distinct classes of substances, such as mercurials and HDAC inhibitors, lead to different gene expression patterns [10,11,12]. Moreover, adaptive and cytotoxic responses of teratogen-exposed differentiating hiPSC led to distinct expression changes [13].

In our most recent work [14], we used a previously-described hiPSC-based test (UKK2) that recapitulates cardiomyogenic differentiation [15,16]. Our procedure, based on substance-induced cytotoxicity and gene expression changes after exposure to 23 known teratogens and 16 non-teratogens at the human peak plasma concentration (C_max_), resulted in an AUC, accuracy, sensitivity, and specificity of 0.96, 0.92, 0.96, and 0.88, respectively, to correctly classify compounds as teratogenic or non-teratogenic [14]. These favorable performance metrics were unexpected, since most of the tested teratogens were not previously reported to specifically compromise cardiomyogenic differentiation. Rather, other developmental alterations are known, such as spina bifida by valproic acid [17] or limb deformations by thalidomide [18]. A possible explanation is that the gene regulatory networks involved in the differentiation of hiPSC to cardiomyocytes in the UKK2 protocol overlap with networks of limb and spine development. Despite the overall favorable results, the hiPSC-cardiomyocyte differentiation test (UKK2) also has some limitations; for example, the teratogen atorvastatin delivered false negative results [14]. A further limitation of the UKK2 protocol is the requirement of a relatively high concentration of the glycogen synthase kinase 3β inhibitor CHIR99021, which induces cell stress and expression changes itself.

In the present study, we used a second hiPSC-based cell system, UKN1, which models neural induction and results in the formation of neuroepithelial precursor cells (NEPs) [19,20]. It is, therefore, a promising cell system to investigate substance-induced developmental toxicity, in particular, developmental neurotoxicity (DNT). The biological meaning of UKN1 is further emphasized by a recent study, where gene expression changes introduced by DNT compounds, such as the above mentioned mercurials and HDAC inhibitors, could be linked to a phenotypic alteration of so-called neural rosettes, a further differentiated form of NEPs [21,22]. 

In the present work, the same set of compounds that was previously investigated in the cardiomyocyte differentiation test UKK2 [14] was analyzed with the UKN1 test, and identical methods of gene expression profiling and statistical evaluation were applied. The following questions were addressed: (1) Can classification performance be further improved by using the UKN1 test system compared to the previously published UKK2? Additionally, are the correct and false predictions of UKN1 and UKK2 similar, or would major differences be obtained? (2) By which degree do the induced gene expression changes in the two cell systems (UKN1 and UKK2) overlap, and are similar or distinct biological motifs affected? (3) Can the classification be improved by combining the results of both tests?

## 2. Materials and Methods

### 2.1. Test Compounds, Teratogenicity Information, and Tested Concentrations

The majority of test compounds were purchased from Sigma-Aldrich (St. Louis, MO, USA). These were 3,3′,5-triiodo-L-thyronine sodium salt (T6397, CAS# 55-06-1), acitretin (PHR1523, CAS# 55079-83-9), ampicillin anhydrous (A9393, CAS# 69-53-4), ascorbic acid (A0278, CAS# 50-81-7), atorvastatin calcium (PHR1422, CAS# 344423-98-9), buspirone hydrochloride (B7148, CAS# 33386-08-2), carbamazepine (C4024, CAS# 298-46-4), chlorpheniramine maleate salt (C3025, CAS# 113-92-8), dextromethorphan HBr (PHR1018, CAS# 6700-34-1), doxorubicin hydrochloride (D2975000, CAS# 25316-40-9), doxylamine succinate (D3775, CAS# 562-10-7), famotidine (F6889, CAS# 76824-35-6), folic acid (F7876, CAS# 89-30-3), isotretinoin (PHR1188, CAS# 4759-48-2), leflunomide (PHR1378, CAS# 75706-12-6), levothyroxine (PHR1613, CAS# 51-48-9), lithium chloride (L4408, CAS# 7447-41-8), magnesium chloride anhydrous (8147330500, CAS# 7786-30-3), methicillin sodium salt monohydrate (1410002, CAS# 7246-14-2), methotrexate (PHR1396, CAS# 59-05-2), methylmercury(II)-chloride (33368, CAS# 115-09-3), paroxetine hydrochloride (PHR1804, CAS# 110429-35-1), ranitidine hydrochloride (R101, CAS# 66357-59-3), retinol (17772, CAS# 68-26-8), sucralose (PHR1342, CAS# 56038-13-2), thalidomide (T144, CAS# 50-35-1), trichostatin A (T1952, CAS#58880-19-6), and valproic acid (PHR1061, CAS# 99-66-1). From Biomol (Hamburg, Germany), actinomycin D (BVT-0089, CAS# 50-76-0), entinostat/MS-275 (Cay13284, CAS# 209783-80-2), panobinostat (Cay13280, CAS# 404950-80-7), vinblastine sulfate salt (Cay11762, CAS# 143-67-9), and vorinostat/SAHA (Cay10009929, CAS# 149647-78-9) were ordered. Hycultec (Beutelsbach, Germany) supplied favipiravir (HY-14768, CAS# 259793-96-9), teriflunomide/A-771726 (HY-15405, CAS# 163451-81-8), and vismodegib (HY-10440, CAS# 879085-55-9). In addition, 5,5-diphenylhydantoin sodium salt (sc-214337, CAS# 690-93-3) and diphenhydramine hydrochloride (sc-204729, CAS# 147-24-0) were obtained from Santa Cruz Biotechnology, Inc (Dallas, TX, USA). All compounds were dissolved and stored at 20,000-fold C_max_ concentrations in 100% DMSO (Carl Roth, Germany) or, alternatively, in distilled water, if soluble, as in [14].

The tested concentrations (1- and 20-fold C_max_) of the teratogens and non-teratogens (Table 1), as well as the information on teratogenicity, correspond to a previously published study [14] with one exception: retinol (vitamin A) was included as a non-teratogen at a C_max_ of 1 µM and as a teratogen for a C_max_ of 20 µM. The rationale is given in the Supplemental Information.

### 2.2. Cultivation of hiPSCs

SBAD2 cells, a human induced pluripotent stem cell line that was originally produced for the StemBANCC project [23], were received from Prof. Marcel Leist (University of Konstanz). The Leibniz-Institute DSMZ (German Collection of Microorganisms and Cell Cultures) validated the cell identity by short tandem repeat profiling. 

For the UKN1 test system, cells were cultured in Essential 8^TM^ (E8) medium (Thermo Fisher Scientific Inc., Waltham, MA, USA) on Biolaminin 521 LN (BioLamina, Sweden) coated culture vessels and in the Cellartis^®^ DEF-CS^TM^ 500 Culture System (Takara Bio, Japan), according to the manufacturers’ guidelines. The cells were cultured following a three- or four-day protocol, i.e., the cells were seeded at a density of 20,000 cells/cm^2^ or 12,000 cells/cm^2^, respectively, and cultured (5% CO_2_, 37 °C) for three or four days until confluency. The medium was changed daily. For dissociation of the cells during each passaging, the dissociation reagent TrypLE^TM^ Select (Thermo Fisher Scientific Inc., Waltham, MA, USA) was used. When cells were passaged in Essential 8^TM^ medium, 10 µM Rho-kinase inhibitor Y-27632 (Cell Guidance Systems, Cambridge, UK) was added to the medium for the first 24 h.

### 2.3. Neuroepithelial Differentiation of hiPSCs and Compound Exposure

The differentiation of SBAD2 hiPSCs to neuroepithelial precursor cells [24] was performed using the UKN1 protocol as published before in [24], with minor changes. Briefly, hiPSCs were seeded in 1 mL pluripotent stem cell (PSC) medium (spiked with a Rho-kinase inhibitor (ROCKi)) per well on extracellular matrix protein-coated 12-well-plates, at a density of 12,000–24,000 cells/cm^2^ on day −3. On day −2 and day −1, the PSC medium was refreshed. On days 0, 1, and 2, the medium was changed to a differentiation medium, which was spiked with 21.6 µM SB431542, 0.64 µM dorsomorphin, 35 ng/mL noggin, and 0.1% DMSO to induce neural differentiation. At the same time, the cells were incubated (5% CO_2_, 37°C) with the test compounds at 1-fold C_max_ and 1.67-, 10-, or 20-fold C_max_ concentrations for a total of 96 h, as well as the vehicle alone (0.1% DMSO). The compounds leflunomide and teriflunomide were tested at a DMSO concentration of 0.5% and compared to a 0.5% DMSO vehicle control. On day 4, the medium was changed to a mixed medium of 75% differentiation medium/25% N2-S and the same concentrations of SB431542, dorsomorphin, and noggin as given above. On day 6, cells were collected for RNA extraction. When no adherent cells were visible upon microscopic inspection, or if the harvested amount of RNA was below 2 µg per well of the 12-well plate, the respective test compound concentration was considered as cytotoxic. A more detailed method description is given in the Supplemental Information.

For each non-cytotoxic compound and concentration (further named ‘condition’) in the UKN1 test, three independent biological replicates were generated. Exceptions from this were as follows: for all 1-fold and 20-fold C_max_ samples of ampicillin, ascorbic acid, buspirone, chlorpheniramine, doxylamine, folic acid, magnesium chloride, methicillin, and valproic acid, as well as for all 1-fold C_max_ samples of famotidine, isotretinoin, methotrexate, paroxetine, and thalidomide, four biological replicates were available. Further exceptions were levothyroxine and methylmercury at the 20-fold C_max_, where two biological replicates were generated. For UKK2, sample composition was as described in [14]. Briefly, for all non-cytotoxic conditions, three independent biological replicates were generated. Exceptions were as follows: 9-cis-retinoic acid at 20-fold C_max_, where two biological replicates were generated, as well as isotretinoin at 1-fold C_max_ and thalidomide at 1-fold and 20-fold C_max_, where six replicates were available in each case.

### 2.4. Affymetrix Microarray Analysis

Total RNA was isolated from sonicated cell lysates with the ExtractMe Total RNA-Kit (Blirt, Gdansk, Poland) according to the manufacturer’s instructions. A NanoDrop2000 instrument (Thermo Fisher Scientific Inc., Waltham, MA, USA) was used to assess the concentration and purity of the isolated RNA. Microarray gene expression studies were performed on Affymetrix Human Genome U133 Plus 2.0 arrays (Affymetrix, Santa Clara, CA, USA) as described previously in [14]. 

### 2.5. Data Pre-Processing

The Affymetrix CEL-files were pre-processed using the frozen robust multi-array average (fRMA) algorithm, obtaining expression values for 54,675 probe sets (PS). For this, the software R, version 4.0.5 [25], and the R-packages affy [26], frma [27], and hgu133plus2frmavecs [28] were used. 

In order to avoid batch effects, the samples were normalized with respect to the control samples. For the majority of samples in UKN1, matched control samples were available, such that differences between the expression values for the non-control samples and the corresponding matched controls were calculated. For the samples where this was not possible, a batch-wise mean of the corresponding control samples was calculated and subtracted from the expression values of the non-control samples. This was the case for all samples of entinostat, lithium chloride, methylmercury, sucralose, and trichostatin A of UKN1, which were compared to the mean of three biological replicates of corresponding control samples, and for all samples of UKK2.

### 2.6. PCA Plots

The principal component analyses (PCA) were based on the normalized expression values, as described above. The replicates for each condition, i.e., for each compound and each concentration, were summarized PS-wise by calculating the mean value.

### 2.7. Limma Analysis

The R-package limma [29] was used for the calculation of differential expression. This is an empirical Bayes method, where the complete set of all PS was considered for the adjustment of the variance estimates of single PS. The resulting moderated t-test is abbreviated here as ‘limma t-test.’ Resulting *p*-values were multiplicity adjusted to control the false discovery rate (FDR) by the Benjamini–Hochberg procedure [30]. The resulting gene list for each compound comprises estimates for fold-change (FC), log2 fold-change, and the *p*-values of the limma t-test (unadjusted and FDR-adjusted).

### 2.8. Classification Based on the Number of Significant Probe Sets (SPS-Procedure)

One classification of the compounds was obtained by using the number of significant probe sets (SPS). A probe set was considered to be a SPS if both the FDR-adjusted *p*-value from the limma t-test was smaller than 0.05 and the absolute value of the FC was larger than 2. The number of SPS was determined for each condition. The highest number of SPS across all conditions was identified for each test system (UKN1 and UKK2). Cytotoxic conditions were assigned test system-wise with the highest number plus five. 

Classification based on these SPS numbers was then conducted as follows: all conditions with the number of SPS higher than a defined threshold were considered to be test-positive, and all conditions with the number of SPS lower than this threshold were considered to be test-negative. This was done test system-wise and concentration-wise, meaning that SPS-numbers of substances at the 1-fold C_max_ were compared to 1-fold-C_max_ thresholds, and of substances at the 20-fold C_max_ to 20-fold C_max_ thresholds, for each test individually. Each threshold was defined as a number of SPS, where the highest accuracy (see below) was achieved for the compound classification. If two or more thresholds led to the same highest accuracy, a threshold was chosen where the highest accuracy and sensitivity were achieved. If two or more thresholds led to the same highest accuracy and sensitivity, a threshold was chosen where the highest accuracy, sensitivity, and specificity were achieved. 

The quality of the classification was assessed by calculating the following measures: sensitivity (true positive rate) was calculated as the number of true positives divided by the sum of true positives and false negatives; specificity (true negative rate) was calculated as the number of true negatives divided by the sum of true negatives and false positives; and accuracy, which was calculated as the proportion of correctly classified conditions. The area under the curve (AUC) was based on the receiver–operator characteristic curve (ROC-curve), where this ROC-curve was calculated as follows: for each possible threshold, the sensitivity and specificity were calculated. The ROC-curve was obtained by plotting all pairs of (1-specificity) and sensitivity against each other. The AUC was determined as the area under this ROC-curve.

For all substances, SPS numbers were available at the 1-fold and 20-fold C_max_, except for leflunomide (LFL), phenytoin (PHE), teriflunomide (TER), and vismodegib (VIS). For these four substances, solubility limits were exceeded at the 20-fold C_max_ so that only SPS numbers at the 1-fold C_max_ were available. In order to integrate these compounds in the classification at the 20-fold C_max_, the SPS numbers from the 1-fold C_max_ were used instead. Implications of this approach are addressed in the discussion. 

### 2.9. Classification Based on Penalized Logistic Regression (Top-1000-Procedure)

Based on the normalized gene expression values, a second classification procedure making use of penalized logistic regression was performed. For this, a leave-one-out cross-validation approach was chosen, where in an iteration over the non-cytotoxic compounds, all samples (i.e., all replicates for the 1-fold and 20-fold C_max_) corresponding to one compound were left out of the dataset in the respective iteration. The 1000 PS with highest variance for the normalized expression values across all samples of the remaining compounds were selected. An ℓ1-regularized logistic regression-based classifier was trained on this dataset and evaluated on the compound that was left out. This yielded a probability for teratogenicity for each replicate at the 1-fold and 20-fold C_max_ of the left-out compound. Over all replicates corresponding to the same concentration, the probabilities were summarized via the mean value, resulting in one average probability for the 1-fold C_max_ and one for the 20-fold C_max_ for each compound. The penalty parameter ‘lambda’ in the ℓ1-regularized logistic regression was optimized via 10-fold cross-validation in order to minimize the mean cross-validated error. Cytotoxic conditions were assigned with a probability of 1.

Using these predicted probabilities for teratogenicity, the classification was conducted as described above for the SPS-procedure, where SPS numbers were used instead of the predicted probabilities. Quality assessment of the classification and integration of the compounds LFL, PHE, TER, and VIS for the 20-fold C_max_ classification were performed as described above as well.

The R-package mlr [31] was used as the framework for the classification tasks, together with the package glmnet [32] for the calculation of the specific classifier.

### 2.10. Combination of the UKN1 and UKK2 Test Systems

The results obtained in the two test systems, UKN1 and UKK2, were combined to classify the conditions in a complementary approach. For both procedures (SPS-procedure and top-1000-procedure) the respective numbers of SPS or predicted probabilities for teratogenicity were compared condition-wise between the two test systems. Cytotoxic conditions were considered as described above for the SPS- and top-1000-procedure. For the combinations ‘min’ and ‘max’, the lower and the higher value was used, respectively. For the combination ‘mean,’ the respective mean value of the two SPS numbers or of the two probabilities of the two test systems was calculated. Definition of the thresholds and calculation of the AUC, accuracy, sensitivity and specificity were conducted as explained above for the SPS- and top-1000-procedure, except for the ‘gene only’ variant, where in ‘mean’ and ‘max,’ all conditions were removed that were cytotoxic in at least one test system.

### 2.11. Venn Diagrams, Top Genes, GO Group Overrepresentation and KEGG Pathway Enrichment

Significant probe sets in the UKN1 and UKK2 tests were used to identify top genes, overrepresented Gene Ontology (GO) groups, and enriched Kyoto Encyclopedia of Genes and Genomes (KEGG) pathways. This was conducted **a.** within the UKN1 test system, and **b.** in a comparative manner between the UKN1 and UKK2 test systems. For each **a.** and **b.**, six analyses were performed, where either all probe sets, only upregulated, or only downregulated SPS were considered, at 1-fold or 20-fold C_max_. 

At first, Venn diagrams were created to compare sets of SPS that were deregulated by non-teratogens and by teratogens. For all analyses, only such SPS were considered that were deregulated by teratogens; SPS that were exclusively deregulated by non-teratogens were not considered. For **b**, the SPS were further separated into three subgroups (further named ‘gene set’): the ‘overlap’ gene set, consisting of all SPS that were deregulated in both test systems, as well as the ‘UKN1′ and ‘UKK2′ gene set, consisting of SPS that were exclusively deregulated by the UKN1 and UKK2 test system, respectively.

For each gene set, ranked top lists of the corresponding PS and genes were determined. As the first level for the ranking, the number of compounds that led to differential expression was determined for each PS. In the ‘overlap’ set, the compounds were separately counted for the UKN1 and the UKK2 test system and summed up. For the second level of the ranking, the arithmetic mean of the log2 fold-changes across the compounds that led to differential expression was calculated. In the ‘overlap’ set, the arithmetic mean was calculated on the basis of absolute log2 fold-changes. For the translation of the top PS into top genes, only the highest ranked PS for each gene was considered. Lower ranked PS representing the same gene were removed. Additionally, for the displayed top-10-lists, only PS with the suffixes _at, _a_at, and _s_at were considered.

Overrepresentation analyses were conducted as follows: for each gene set, SPS were assigned to GO groups according to their biological process. It was statistically tested whether more PS in the respective GO groups were differentially expressed than expected at random, using Fisher’s exact test. This procedure was conducted in a bottom-up approach (‘elim’ approach) with respect to the GO group hierarchy. PS that were already contained in a more specific GO group were not considered again in more general groups [33]. For **b**, significant GO groups for each gene set were determined, where a GO group was called significant if the FDR-adjusted *p*-value of the ‘elim’ method was smaller than 0.05, and further analyzed with respect to their appearance in the UKN1 and UKK2 system using Venn diagrams.

Additionally, SPS were assigned to KEGG pathways. Fisher’s exact test was used to statistically test whether more PS in the respective pathway were differentially expressed than expected at random.

GO group analyses were conducted using the R package topGO [34], and KEGG pathway analyses were conducted using the R package clusterProfiler [35].

### 2.12. Classification Based on Seven Significantly Deregulated Top Genes in RT-qPCR

For the measurement of gene expression changes in UKN1 with RT-qPCR, the same RNA samples as for the microarray studies were used. Therefore, RNA was at first transcribed into complementary deoxyribonucleic acid (cDNA) with the ‘High-Capacity cDNA Reverse Transcription Kit’ (Thermo Fisher Scientific Inc., Waltham, MA, USA) following the manufacturer’s instructions. Then, the RT-qPCR measurements were performed using QuantiFast SYBR^®^ Green PCR master mix and QuantiTect Primer Assays (Qiagen, Germany) for the genes *CTHRC1*, *LMAN1*, *PNCK*, *RBM24*, *SEMA3C*, *SLIT2,* and *ZNF385B* in an ABI 7500 real-time PCR system (Thermo Fisher Scientific Inc., Waltham, MA, USA). Expression values were normalized to the housekeeping gene *TBP*, for which self-designed primers were used (5′--> 3′; forward: GGGCACCACTCCACTGTATC; reverse: GCAGCAAACCGCTTGGGATTATATTCG; Eurofins, Luxembourg). Fold-changes were obtained by using the 2^−ΔΔCT^-method [36]. Gene expression changes with an absolute fold-change larger than 2 and a *p*-value smaller than 0.05 were considered to be significant. The level of significance was obtained by application of a two-sided one-sample t-test using the software Excel (Microsoft, USA). Cytotoxic conditions were integrated as follows: for upregulated genes (*CTHRC1*, *SMEA3C,* and *SLIT2*), the ΔΔC_T_ mean-value was set as 2 with a *p*-value of 0.01; for downregulated genes (*LMAN1*, *PNCK*, *RBM24,* and *ZNF385B*) the ΔΔC_T_ mean-value was set as −1.6 with a *p*-value of 0.01. In order to classify the results based on significant gene expression changes (further called ‘SPS-like’), the number of genes where a substance caused a significant deregulation was counted for each condition. If that number was higher than 0, the condition was test-positive, otherwise it was test-negative. Furthermore, a penalized logistic regression was performed as described above for the top-1000-procedure in order to classify the results, where in each iteration of the leave-one-out procedure, all seven measured genes were considered (further named ‘Top-1000-like’). The identification of false and true negatives and positives, as well as the calculation of the AUC, accuracy, sensitivity, and specificity was done as explained above for the SPS- and top-1000-procedure. 

For all substances, 3–4 biological replicates were considered, except for acitretin, entinostat, lithium chloride, and trichostatin A at 1-fold C_max_, as well as dextromethorphan at 20-fold C_max_, where only two biological replicates were analyzed. Since no biological replicates were available for classification of levothyroxine at 20-fold C_max,_ the same values as for 1-fold C_max_ were considered instead.

## 3. Results

### 3.1. Gene Expression Profiling

**Figure 1:**
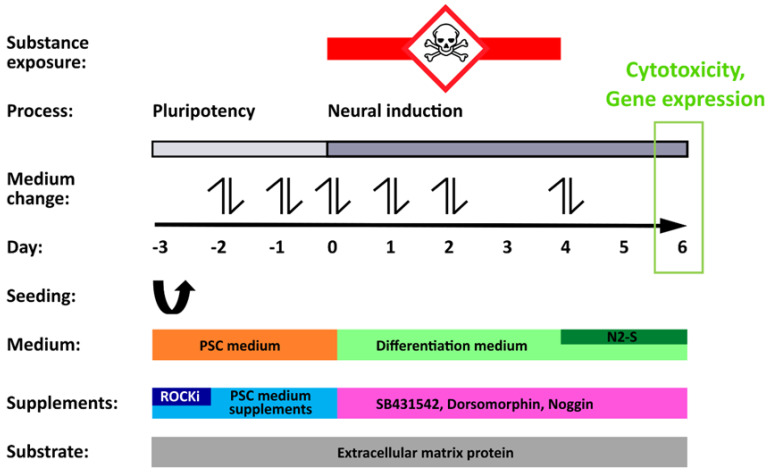
Schematic representation of the UKN1-test (modified from [20,37]). The overview scheme depicts the differentiation protocol, important experimental steps, and the principal of the toxicity assay. In the pluripotency phase (day −3 to 0), hiPSCs were cultured in a pluripotent stem cell (PSC) medium to maintain their pluripotent state. Factors that inhibited Rho-kinase (ROCKi) were additionally given on the day of seeding (day −3) to support the survival of hiPSCs seeded as single cells on extracellular matrix proteins. From day 0 onwards, the change to a differentiation medium spiked with SB431542, dorsomorphin, and noggin initiated neuroectodermal differentiation of the cells. Simultaneously, cells were exposed to test compounds for a total of 96 h. On day 4, substances were withdrawn and addition of 25% N2-S further enhanced the neural differentiation process. On day 6, compound-induced cytotoxicity was determined and the cells were harvested for gene array analysis. Media changes were conducted as indicated (double arrows) on the days −2, −1, 0, 1, 2, and 4.

Two concentrations (1-fold C_max_ and 20-fold C_max_) of 23 teratogenic and 16 non-teratogenic compounds (Table 1) were analyzed using the hiPSC-based UKN1 test (Figure 1), where hiPSCs were differentiated to neuroepithelial precursor cells while being exposed to test compounds with the aim of detecting developmental toxicity. The selected concentrations corresponded to 1-fold and 20-fold C_max_ reported in human blood after therapeutic doses (Table 1). Substance-induced gene expression changes were detected by microarrays after an incubation period of 4 days with the test compounds, followed by a washout period (without test compounds) of 2 days (Figure 1).

**Figure 2:**
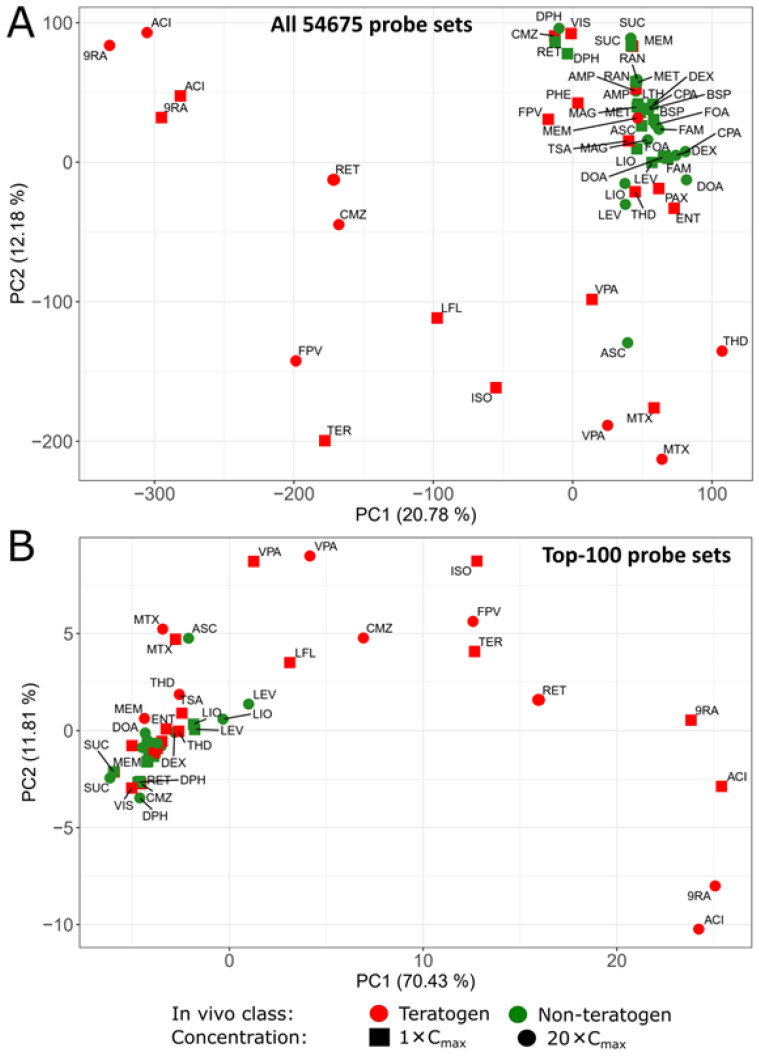
Principal component analysis (PCA) of the teratogenic and non-teratogenic compounds in the UKN1 test. Two PCA-Plots are presented of (**A**) all 54,675 probe sets and (**B**) the 100 probe sets with the highest variance across the mean of the condition-wise samples. Green and red tags represent in vivo non-teratogens and teratogens, respectively. 1-fold C_max_ and 20-fold C_max_ concentrations are indicated by squares and circles, respectively. The distribution of the data points on the *x*-axis is given by the principal component (PC) 1 and on the *y*-axis by PC2. The percentages in parentheses denote the proportion of explained variance for the respective PC. Compound abbreviations are explained in Table 1.

In a principal component analysis (PCA) considering all 54,675 analyzed probe sets (Figure 2A), as well as the 100 probe sets with the highest variance (Figure 2B), all non-teratogens except for ascorbic acid (ASC, abbreviations defined in Table 1) at the 20-fold C_max_ formed a narrow cluster, which was intermixed with 11 of 25 non-cytotoxic teratogenic conditions, such as phenytoin (PHE) or methylmercury (MEM), which deregulated either none or only a small number of probe sets (Table 2). The high percentage of explained variance by PC1 and PC2 (82.24%) in the top-100-PCA suggests that only a small subset of genes is sufficient to identify teratogens that cause major gene expression changes. 

**Figure 3:**
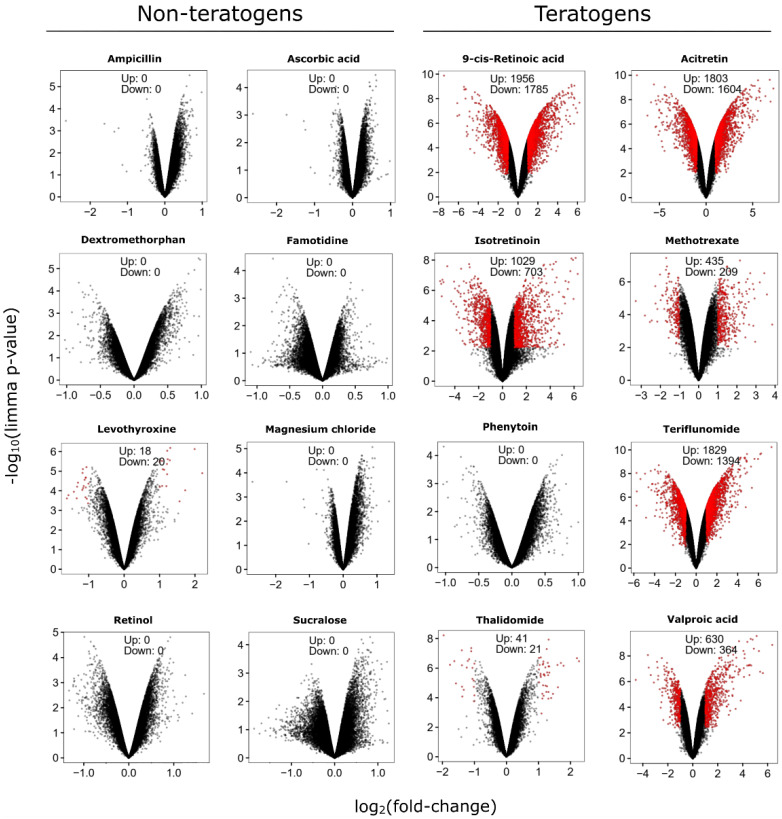
Volcano plots of deregulated probe sets of selected test compounds in the UKN1 test. Volcano plots show genome-wide gene expression changes in substance-exposed SBAD2 cells for a representative subset of known teratogens and non-teratogens at therapeutic 1-fold C_max_ concentrations. Each dot represents one out of 54,675 probe sets from the Affymetrix gene chips. The fold-change of the differentially-expressed probe sets in substance-exposed cells is given on the *x*-axis in log2-values, and the corresponding *p*-values of the limma-analyses are given on the *y*-axis in negative log10-values. Red dots represent probe sets with a statistically significant, FDR-adjusted *p*-value < 0.05 and an absolute fold-change > 2. The numbers of up- and downregulated red-dot-probe sets are indicated.

Genome-wide expression changes were illustrated in volcano plots for a representative set of non-teratogenic and teratogenic test compounds (Figure 3). Plots of all compounds and concentrations (further named ‘conditions’) are available in the Supplemental Information. In subsequent analyses, all probe sets that were at least 2-fold deregulated and statistically significant with a false discovery rate (FDR) adjusted *p*-value < 0.05 were identified. In general, a large number of significantly deregulated probe sets (SPS) was obtained for many teratogens, whereas none or only a few were observed for the non-teratogens (Table 2). Raw data are available in the Gene Expression Omnibus (GEO) database under the accession number GSE209962.

### 3.2. Gene Expression-Based Classification to Identify Teratogens and Non-Teratogens by the UKN1 Test

**Figure 4:**
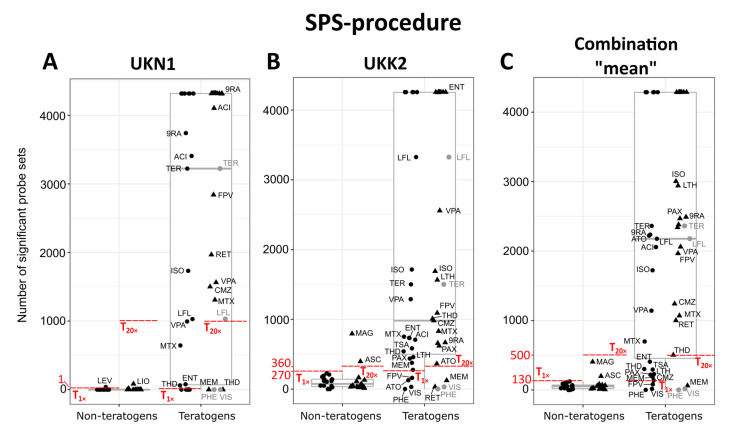

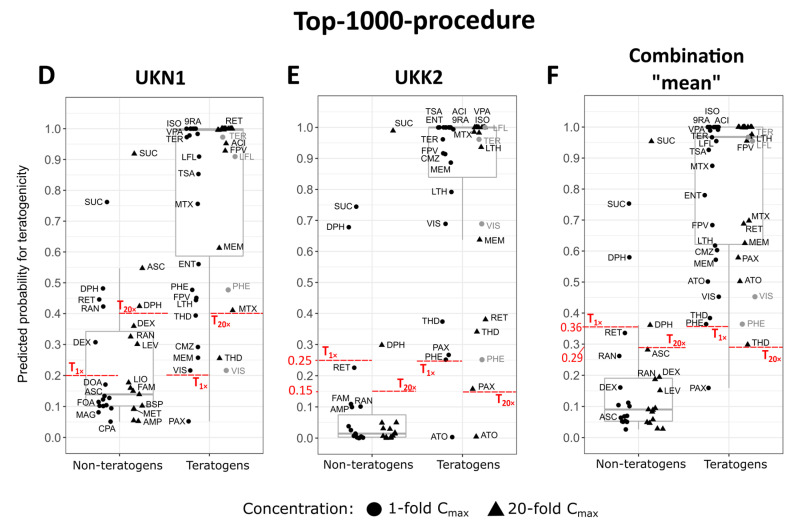
Classification of the teratogenic and non-teratogenic compounds by the SPS-procedure, a method based on the number of significantly deregulated probe sets (SPS), and the top-1000-procedure, a penalized logistic regression-based technique using the 1000 probe sets with the highest variance. (**A**–**C**) SPS-procedure. The number of SPS for each test condition is given on the *y*-axis and the *x*-axis marks non-teratogens and teratogens (compound abbreviations are explained in Table 1). 1-fold-C_max_ conditions are indicated with black dots, 20-fold C_max_ conditions with black triangles. Grey dots represent the numbers of SPS at 1-fold C_max_ of LFL, PHE, TER, and VIS, which were compared to 20-fold C_max_ thresholds. For the UKK2 test, SPS numbers were adapted from Cherianidou et al. 2022, but retinol at 20-fold C_max_ was considered as a teratogen. SPS numbers above or below the thresholds T_1×_ and T_20×_ (red dashed lines) for 1-fold and 20-fold C_max_ conditions, respectively, were considered to be test-positive or test-negative. Cytotoxic conditions were considered to be test-positive and were assigned with a high number of SPS (UKN1: 4318; UKK2: 4257). Thresholds UKN1 (**A**): 1-fold-C_max_: 1 SPS; 20-fold-C_max_: 1000 SPS; UKK2 (**B**): 1-fold-C_max_: 270 SPS; 20-fold-C_max_: 360 SPS; combination ‘mean’ (**C**): 1-fold-C_max_: 130 SPS; 20-fold-C_max_: 500 SPS. (**D**–**F**) Top-1000-procedure. The predicted probability for teratogenicity is given on the *y*-axis, and the *x*-axis marks non-teratogens and teratogens. Cytotoxic conditions were considered to be 100% test-positive (predicted probability of 1.0). Thresholds UKN1 (**D**): 1-fold-C_max_: 0.2; 20-fold-C_max_: 0.4; UKK2 (**E**): 1-fold-C_max_: 0.25; 20-fold-C_max_: 0.15; combination ‘mean’ (**F**): 1-fold-C_max_: 0.36; 20-fold-C_max_: 0.29.

We used two techniques to classify the test compounds analyzed by the UKN1 test: (i) the ‘SPS-procedure’ that is exclusively based on the number of SPS, and (ii) the ‘top-1000-procedure’, a penalized logistic regression-technique based on the 1000 probe sets with the highest variance and leave-one-out-cross-validation (Figure 4A,D), as previously described [14]. A compound was classified as test-positive or test-negative if the test result, i.e., SPS number in the SPS-procedure and the predicted probability for teratogenicity in the top-1000-procedure, was above or below a defined threshold. Moreover, cytotoxicity was included by considering the in vitro result as test-positive when the tested concentration was cytotoxic. Both procedures identified most teratogens as test-positive and most of the non-teratogens as test-negative, resulting in a high rate of true positives and true negatives (Supplementary Table S1). 

The top-1000-procedure was consistently more sensitive than the SPS-procedure, i.e., it classified more teratogenic compounds as true positives, and it also obtained higher AUC-values (Table 3). At the 20-fold C_max_ concentration, it reached the highest values for the AUC (0.95) and sensitivity (0.92) compared to the SPS-procedure (0.90 and 0.83, respectively). However, the non-teratogens ASC, diphenhydramine (DPH), and sucralose (SUC) were misclassified, as well as the teratogens thalidomide (THD) and vismodegib (VIS) (Table 4). In contrast, the SPS-procedure was consistently more specific than the top-1000-procedure (Table 3). At 20-fold C_max_, all non-teratogens were correctly classified, but four of the teratogens (MEM, PHE, THD, and VIS) were not identified as test-positives (Table 4). A comprehensive overview of the classification results (true/false positive; true/false negative) and the predicted probabilities of all compounds in all tests can be found in Supplementary Tables S1 and S2, respectively.

### 3.3. Biological Interpretation of Genes Differentially Expressed in the UKN1 Test

**Figure 5:**
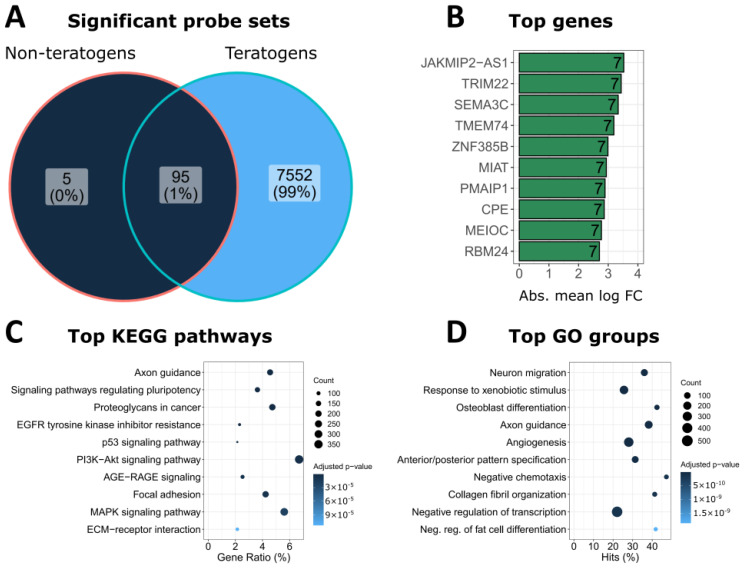
Biological interpretation of genes differentially expressed after exposure of hiPSC to teratogens at 20-fold C_max_. (**A**) Number of significant probe sets (log2 fold change > 1; adjusted *p*-value < 0.05) induced by non-teratogens and teratogens at the 20-fold C_max_ (including also 10-fold C_max_ carbamazepine and 1.67-fold C_max_ VPA). (**B**) Top-10 genes from the 7647 SPS deregulated by teratogens. The number in the bar indicates the number of compounds that deregulated the specific gene. The absolute mean log2 fold-change of each gene is given on the *x*-axis. A comprehensive gene list is given in the Supplementary Excel-file 1. (**C**) KEGG pathway enrichment analysis of the 7647 SPS deregulated by teratogens. The ten KEGG pathways with the lowest adj. *p*-values are given. Full names and complete KEGG-pathway lists are given in the Supplementary Excel-file 2. ‘‘Count:’’ number of significant genes from A linked to the KEGG pathway. ‘‘Gene Ratio:’’ percentage of significant genes associated with the pathway compared to the number of all significant genes associated with any pathway. (**D**) The ten GO groups with the lowest adj. *p*-values from all significantly (adj. *p*-value < 0.05) overrepresented GO groups in the 7647 SPS deregulated by teratogens. The names of the GO groups have been shortened. Full names and complete GO group lists can be found in the Supplementary Excel-file 3. ‘Count:’ number of significant genes from A linked to the GO group. ‘Hits:’ percentage of significant genes compared to all genes assigned to the GO group.

Analysis of the significantly altered probe sets at 20-fold C_max_ showed that a total of 7647 (7552 + 95) different PS were significantly influenced by the 23 teratogens, while the 16 non-teratogenic compounds only altered the expression of 100 PS (Figure 5A). Among the genes altered by most individual teratogens (Figure 5B) were *SEMA3C*, a member of the class of semaphorins that function as axonal growth guidance molecules [38,39]; *MIAT*, a long non-coding RNA which is expressed in neurons and plays a role in retinal development [40,41], and carboxypeptidase E (*CPE*), which is involved in the biosynthesis of neuropeptides [42]. KEGG pathway analysis and the analysis of GO groups resulted in ‘axon guidance’ and ‘neuron migration’ as the top overrepresented motifs (Figure 5C,D), which correspond to the intended neuroepithelial differentiation in UKN1. Moreover, genes involved in several pathways relevant in developmental processes were overrepresented, such as PI3K-Akt, MAPK, P53, and EGFR signaling (Figure 5C). Similar top genes, KEGG pathways, and GO groups were obtained when probe sets at the 1-fold C_max_, or only up- or downregulated probe sets at the 20-fold C_max_, were analyzed (Supplementary Figures S1–S5).

### 3.4. Comparing the UKN1 Test with UKK2 for the Classification of Developmental Toxicity

We next compared the performance of the here-described UKN1 test system to the previously published UKK2-based test [14], after adjusting for retinol as a teratogen at 20-fold C_max_ (Figure 4B,E), which was carried out based on the rationale given in the Supplemental Information. From this comparison, the following conclusions could be drawn: (i) the use of gene expression and cytotoxicity data together led to a higher test-performance in both tests than the use of gene expression or cytotoxicity data alone (Table 3); (ii) the SPS-procedure was more specific than the top-1000-procedure, but the teratogens PHE and VIS were consistently misclassified as false-negatives; (iii) the top-1000-procedure was more sensitive than the SPS-procedure, but consistently misclassified the non-teratogens DPH and SUC as false-positives; (iv) the UKN1 test performed better at 20-fold C_max_; and (v) the UKK2 test performed better at the 1-fold C_max_.

Overall, UKN1 and UKK2 showed a high congruency in their classifications of the tested compounds. When the tests were compared to each other at their optimal ‘working concentration,’ that is, UKN1 at 20-fold C_max_ and UKK2 at the 1-fold C_max_, 34 of the 38 substances (89%, retinol not considered) were identically classified in the SPS-procedure, as well as in the top-1000-procedure (Table 4). Among the exceptions were atorvastatin (ATO), which was a true-positive in UKN1 but false-negative in UKK2, and thalidomide, which was a true-positive in UKK2 but a false negative in UKN1. Different classifications by the two tests (including both the SPS and the top-1000-procedure) were further observed for ASC, ATO, favipiravir (FPV), MEM, THD, and VIS. 

### 3.5. Overlap of Teratogen-Induced Expression Patterns in UKN1 and UKK2

**Figure 6:**
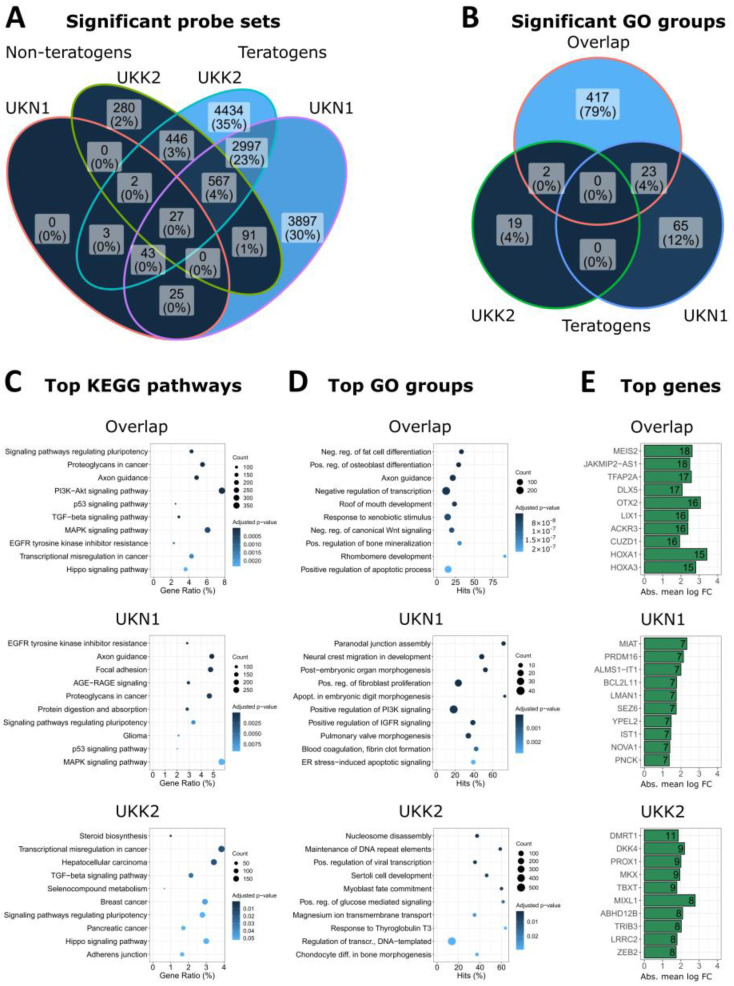
Biological interpretation and comparison of genes differentially expressed in the UKN1 and UKK2 test after exposure of hiPSC to teratogens at 20-fold C_max_. (**A**) Number of significant probe sets (log2 fold change >1; adjusted *p*-value < 0.05) induced by non-teratogens and teratogens at the 20-fold C_max_ (including also 10-fold C_max_ carbamazepine and 1.67-fold C_max_ VPA). The following gene sets were defined: ‘Overlap:’ SPS that were deregulated by teratogens in UKN1 and UKK2 (3634 SPS); ‘UKN1′ and ‘UKK2:’ SPS that were deregulated by teratogens exclusively in UKN1 (4013 SPS) and UKK2 (4885 SPS), respectively. (**B**) Number of significantly (adj. *p*-value < 0.05) overrepresented GO groups in the gene sets ‘Overlap,’ ‘UKN1,’ and ‘UKK2.’ (**C**) KEGG pathway enrichment analysis of the gene sets ‘Overlap,’ ‘UKN1,’ and ‘UKK2.’ The ten KEGG pathways with the lowest adj. *p*-values are given. Full names and complete KEGG-pathway lists are given in the Supplementary Excel-file 4. ‘‘Count:’’ number of significant genes from A linked to the KEGG pathway. ‘‘Gene Ratio:’’ percentage of significant genes associated with the pathway compared to the number of all significant genes associated with any pathway. (**D**) The ten GO groups with the lowest adj. *p*-values from all significantly (adj. *p*-value <0.05) overrepresented GO groups in each gene set. The following adjustments applied here: ‘Overlap’ included all GO groups encompassed by the overlap-circle in B (442 GO groups); ‘UKN1′ (65 GO groups) and ‘UKK2′ (19 GO groups) only considered remained GO groups. The names of the GO groups were shortened. Full names and complete GO group lists can be found in the Supplementary Excel-file 5. ‘‘Count:’’ Number of significant genes from A linked to the GO group. ‘‘Hits:’’ percentage of significant genes compared to all genes assigned to the GO group. (**E**) Top-10 genes deregulated by teratogens within each gene set. The number in the bar indicates the number of compounds that deregulated the specific gene. The absolute mean log fold-change of each gene is given on the *x*-axis. A comprehensive gene list is provided in the Supplementary Excel-file 6.

That similar classification results were obtained for both the UKN1 and the UKK2 tests may appear surprising since the protocols recapitulate different biological processes: differentiation to NEPs for UKN1 versus myoblast development for UKK2. In order to gain more insight into the involved genes and pathways, we compared gene expression changes for both cell systems. A relatively large overlap of teratogen-induced gene expression changes was obtained for both tests; nevertheless, the number of genes exclusively influenced by either the UKN1 or UKK2 test was higher than the number altered by both tests, for example, 4013 or 4885 genes, respectively, versus 3634 genes for all probe sets at 20-fold C_max_ (Figure 6A). Analyzing the numbers of significantly overrepresented GO groups in the gene sets of the overlap, UKN1 (only) and UKK2 (only) demonstrated that 83% of all significantly overrepresented GO groups were obtained from the overlap (Figure 6B). The most significant KEGG pathways and GO groups of the UKN1 (only) gene set included ‘axon guidance’ and ‘neuronal crest migration’ (Figure 6C,D), in agreement with the above-reported motifs in the complete set of genes altered in UKN1 (Figure 5C,D). The UKK2 (only) gene set resulted in overrepresentation of ‘myoblast fate commitment’, in agreement with the purpose of this protocol, but unexpectedly also resulted in enriched cancer motifs, such as ‘hepatocellular cancer,’ ‘breast cancer,’ and ‘pancreatic cancer’ (Figure 6C,D). A conspicuous feature of the overlap gene set was overrepresentation of signaling pathways known to be relevant in embryonic development and carcinogenesis, such as PI3K-Akt, P53, TGF-beta, EGFR, and Hippo (Figure 6C), similar to the pathways obtained for UKN1 (Figure 5C). The overlap of the top probe sets of UKN1 and UKK2 included genes that play a role in both neural crest and cardiac development, such as MEIS2 and the helix-span-helix transcription factor TFAP2A (AP-2α) (Machon et al., 2015; Brewer et al., 2002) (Figure 6E). In conclusion, while UKN1 and UKK2 only overlapped by 27% at the level of significant probe sets, there was a more than 80% overlap when the biological motifs, such as GO groups, were considered. Similar results were obtained when probe sets at the 1-fold C_max_ were investigated (Supplemental Figures S6–S8), or when only up- or downregulated probe sets at the 20-fold C_max_ were analyzed (Supplemental Figures S9 and S10).

### 3.6. Combining UKN1 and UKK2 Improves the Classification Performance

Using the SPS numbers and the predicted probabilities of UKN1 and UKK2, we finally investigated whether combining both tests could further improve the outcome of the classification. Therefore, three different combinations were created where for each condition, either the lowest (‘min’) or the highest value (‘max’) from one of the tests was used, or where the arithmetic mean of both tests was calculated (‘mean’). From these combinations, the ‘mean’ value (Figure 4C,F) improved the outcome of both the top-1000- and the SPS-procedure (Table 3) compared to each test alone. Furthermore, the top-1000-procedure at 20-fold C_max_ classified all compounds except DPH and SUC correctly (Table 4), thus improving the AUC, accuracy, and specificity to 0.98, 0.95, and 0.87, respectively, while reaching a sensitivity of 1.0 (Table 3). In addition, the SPS-procedure led to an AUC, accuracy, sensitivity, and specificity of 0.91, 0.92, 0.88, and 1.0, respectively, at 20-fold C_max_, with only three misclassified compounds, namely MEM, PHE, and VIS, as false-negatives (Table 4). The combination ‘max’ (Supplementary Figure S11B,D) improved the classification with the SPS-procedure, but not the top-1000-procedure (Supplementary Table S3). Finally, ‘Min’ (Supplementary Figure S11A,C) did not improve the classification compared to each test alone. The condition-wise results for all the combined tests are available in the Supplementary Materials (Supplementary Table S1: Classification of the in vitro test results; Supplementary Table S2: Predicted probabilities for teratogenicity; Supplementary Table S4: Cytotoxicity status and number of SPS).

### 3.7. Top Genes-Based Classification in UKN1 by RT-qPCR

Seven so-called ‘top genes’ were identified based on the definition that they were altered by the highest numbers of teratogens: *CTHRC1*, *LMAN1*, *PNCK*, *RBM24*, *SEMA3C*, *SLIT2,* and *ZNF385B*. We tested whether these genes could be used for compound classification in a simplified approach that utilized RT-qPCR instead of gene arrays. The RT-qPCR data highly correlated with the gene array analysis with r = 0.97 (Figure S12). Significant changes (absolute fold-change > 2, *p*-value < 0.05) were only observed for the teratogenic substances. By considering each condition that showed at least one significantly deregulated top-gene or that was cytotoxic (SPS-like) as test-positive, an accuracy, sensitivity, and specificity of 0.90, 0.83, and 1.0, respectively, could be reached at 20-fold C_max_ (Table 3). Interestingly, the result of the classification based on the seven top-genes was identical to the classification obtained by the SPS-procedure (Table 4). The top-1000-like classification using logistic regression and leave-one-out cross-validation (Supplementary Figure S13) led to a lower accuracy and specificity of each 0.87, but a higher sensitivity of 0.88 (Table 3). Further information is available as Supplemental Information: classification of all conditions (Supplementary Table S1), predicted probabilities for teratogenicity (Supplementary Table S2), gene expression diagrams (Supplemental Figures S14–S20), and data of all seven genes (Supplementary Excel-File 7).

## 4. Discussion

The identification of teratogenic substances that affect embryonic development and lead to congenital malformations in newborns remains an important task in toxicity testing. However, conventional in vivo tests are expensive, and the number of required experimental animals is high. As a result, alternative test strategies, such as stem cell-based in vitro tests, are urgently needed [1,2,3]. In the current study, we used the UKN1 test, an approach based on hiPSCs differentiating to NEPs, to identify developmental toxicants in vitro. Using transcriptomics, we analyzed the effect of 23 teratogenic and 16 non-teratogenic compounds at concentrations of 1-fold and 20-fold C_max_, and classified the results by using either the number of significantly deregulated probe sets (SPS-procedure) or a penalized logistic regression procedure based on the 1000 probe sets with the highest variance (top-1000-procedure). Together with cytotoxicity data, the SPS-procedure at the 20-fold C_max_ was able to classify the teratogens with an AUC, accuracy, sensitivity, and specificity of 0.90, 0.90, 0.83, and 1.0, respectively. Alternatively, a higher sensitivity but lower specificity was obtained for the top-1000-procedure at the 20-fold C_max_ with the AUC, accuracy, sensitivity, and specificity at 0.95, 0.87, 0.92, and 0.80, respectively.

Compared to the previously published UKK2 test, which used the same set of compounds and techniques to analyze and classify compound-induced effects on gene expression, but employed a cardiomyogenic rather than a neuronal differentiation process [14], the classification outcome was surprisingly similar and overlapped for 90% of the analyzed compounds. In addition, the efficiency of UKK2 to detect teratogens was very similar to UKN1, even though UKK2 performed best at 1-fold C_max_ and not at 20-fold C_max_ like UKN1. These findings led to the question of whether a combination of both tests could further improve the classification metrics. Indeed, when the arithmetic means of the results at 20-fold C_max_ from both tests were combined, the AUC, accuracy, sensitivity, and specificity of the SPS-procedure slightly improved to 0.91, 0.92, 0.88, and 1.0, respectively, and to 0.98, 0.95, 1.0, and 0.87 for the top-1000-procedure.

Although both tests classified most of the compounds correctly and could clearly determine if a compound and concentration influenced gene expression, some limitations should be considered. First, the information on cytotoxicity was required to obtain the best classification performance, as some teratogens were cytotoxic at the tested 1-fold or 20-fold C_max_, especially in UKN1. This observation was unexpected, since, when designing the present study, we did not expect cytotoxicity so close to the therapeutic C_max_. Thus, a cytotoxicity assay was not included, but the cytotoxicity information was derived from the observation of cell detachment from the culture dishes and the corresponding lack of a sufficient amount of RNA for gene array measurement. In future, studies should integrate quantitative cytotoxicity analysis and consider this information for the classification. Moreover, a concentration-dependent gene expression analysis should be performed instead of restricting the analysis to the here-chosen 1-fold and 20-fold C_max_. In combination with cell viability assays, such an approach would directly link gene expression and cell viability and enable the precise discrimination between teratogenicity- and cytotoxicity-related gene expression alterations. 

The second limitation is the consistent misclassification of some compounds. The SPS-procedure was unable to identify PHE and VIS as teratogens, whereas the top-1000-procedure misclassified the non-teratogens DPH and SUC as teratogens. Although DPH-induced toxicity is documented [43], and some effects were also reported for SUC in mesenchymal stromal cells and mice [44,45,46], the positive test results point to a shortcoming of the top-1000-procedure rather than to an actual adverse effect. Nevertheless, the top-1000-procedure was cross-validated in contrast to the SPS-procedure, thus avoiding the problem of overfitting the data.

For the comparison of the accuracy (and further performance metrics) at the 1-fold and the 20-fold C_max_, the challenge had to be addressed that four compounds (LFL, PHE, TER, and VIS) exceeded their solubility limits at the 20-fold C_max_. In order to, nevertheless, allow a comparison, the SPS numbers and predicted probabilities obtained for the 1-fold C_max_ were also used for the classification at the (not testable) 20-fold C_max_ for these four compounds. The here-applied procedure to also use the 1-fold C_max_ results for calculations (e.g., of accuracy) at the 20-fold C_max_ was chosen in order to avoid these compounds influencing the comparison, since their classifications were identical at the 1-fold and 20-fold C_max_ in all cases (except for VIS in UKN1 for the top-1000-procedure). The difference in accuracy for both concentrations must be due to the other compounds that could all be tested at both 1- and 20-fold C_max_. In principle, an alternative approach would have been to calculate the classifiers without the compounds LFL, PHE, TER, and VIS. This approach was not chosen because two of these four compounds, PHE and VIS, led to false negative classifications in the SPS-procedure; classifier construction without PHE and VIS may, therefore, have resulted in overoptimistic performance metrics. 

Another limitation of the present study is that the influence of the different exposure schedules on gene expression and cytotoxicity has not yet been systematically evaluated. In the UKK2 test, a 24 h incubation period with test substances was used and gene expression was analyzed immediately afterwards. In contrast, a four-day incubation period followed by two days of a test compound-free washout period was applied for the UKN1 test. The intention of the washout period was to allow the recovery of the cells from reversible compound-induced expression changes while only retaining irreversible expression changes, for example, due to the differentiation to aberrant cell types. These differences in the protocols could be the reason why, for example, the teratogens ATO and THD were differently classified by UKN1 and UKK2. ATO only had small effects on gene expression and was not cytotoxic in the UKK2 test, resulting in a false-negative classification, whereas, possibly due to the longer incubation period, ATO was cytotoxic in the UKN1 test, resulting in a true-positive classification. In contrast, UKN1 was not able to clearly identify the well-known teratogen THD, a limitation that requires further investigations.

Analysis of KEGG pathways and GO groups of individual probe sets that were significantly influenced by the teratogens in the UKN1 test demonstrated that genes involved in axon guidance, neuron migration, or anterior/posterior specification were overrepresented. These findings suggest that the differentiation process of stem cells to NEPs may be compromised by the test substances. The same set of test compounds analyzed in the present work was also previously analyzed in the UKK2 system [14] that recapitulates the differentiation of stem cells to myoblasts, thereby offering the possibility to compare both differentiation protocols. Both UKN1 and UKK2 showed a large overlap of overrepresented GO groups, e.g., 83% for all probe sets at 20-fold C_max_, although the overlap of significant probe sets was smaller (27%). Moreover, UKN1 and UKK2 overlapped for a substantial number of signaling pathways critical for developmental processes, such as PI3K-Akt, P53, TGF-beta, MAPK, EGFR, and Hippo, which are influenced by the teratogens in both tests. This overlap may explain why UKN1 and UKK2 led to a similar classification of most teratogens and non-teratogens, although the applied protocols and induced differentiation processes were quite different.

Finally, validation of the gene array data by RT-qPCR showed a high correlation of gene expression changes obtained for both methods for seven selected top-genes. Interestingly, the top-genes, which were selected because their expression was influenced by the largest numbers of teratogens, allowed classification with identical sensitivity and specificity as the SPS-procedure with genome-wide data. Thus, by selecting a small, well-chosen set of top genes, it may be possible to identify teratogens with targeted gene expression analysis in a manner that is cost-efficient, instead of using cost-intensive whole-transcriptome analysis.

In conclusion, both the UKN1 and UKK2 tests allow for the identification of teratogens at human-relevant concentrations. Despite recapitulating distinct differentiation processes, a high degree of overlap in the classification results was obtained by both tests, likely because similar pathways were affected. A combined analysis of tests that differentiate hiPSCs into different germ layers may even further improve the prediction of developmental toxicants.

## Figures and Tables

**Table 1:** Substances and concentrations in the UKN1 test (adapted from Cherianidou et al., 2022).

Compound	Abbreviation	Pregnancy Category ^a^	Drug Class	Concentration [µM]	
				1-Fold C_max_^b^	20-Fold C_max_^b^
**Non-teratogens**					
Ampicillin	AMP	A, B	Antibiotic	107	2140
Ascorbic acid	ASC	A	Vitamin	200	4000
Buspirone	BSP	B	Anxiolytic, serotonin 5-HT1A receptor agonist	0.0244	0.488
Chlorpheniramine	CPA	B	Antihistamine, histamine H1 receptor antagonist	0.0304	0.608
Dextromethorphan	DEX	A	Antitussive and psychoactive agent	0.15	3
Diphenhydramine	DPH	A, B	Antihistamine, histamine H1 receptor antagonist	0.3	6
Doxylamine	DOA	A	Antihistamine, histamine H1 receptor antagonist	0.38	7.6
Famotidine	FAM	B	Antihistamine, histamine H2 receptor antagonist	1.06	21.2
Folic acid	FOA	A	Vitamin	0.38	7.6
Levothyroxine	LEV	A	Synthetic thyroid hormone	0.077	1.54
Liothyronine	LIO	A	Synthetic thyroid hormone	0.00307	0.06145
Magnesium (chloride)	MAG	n/a	Dietary supplement	1200	24,000
Methicillin	MET	B	Antibiotic	140	2800
Ranitidine	RAN	B	Antihistamine, histamine H2 receptor antagonist	0.8	16
Retinol ^d^	RET	n/a	Vitamin and retinoid	1 ^d^	-- ^d^
Sucralose	SUC	n/a	Artificial sweetener	2.5	50
**Teratogens**					
9-cis-Retinoic acid	9RA	D	Retinoid, RAR and RXR ligand	1	20
Acitretin	ACI	X	Retinoid, RAR activator	1.2	24
Actinomycin D	ACD	D	Antineoplastic agent, RNA synthesis inhibitor	0.1	2
Atorvastatin	ATO	X	Antilipemic agent, HMG-CoA reductase inhibitor	0.54	10.8
Carbamazepine	CMZ	D	Anticonvulsant, voltage-gated sodium channel blocker	19	10-fold C_max_: 190 ^c^
Doxorubicin	DXR	D	Antineoplastic agent, affects DNA and related proteins; produces ROS	1.84	36.8
Entinostat	ENT	n/a	Potential antineoplastic agent, HDAC inhibitor	0.2	4
Favipiravir	FPV	n/a	Antiviral drug, selective inhibitor of RNA polymerase of influenza virus	382	7600
Isotretinoin	ISO	X	Retinoid, RAR ligand	1.7	34
Leflunomide	LFL	X	Anti-inflammatory agent, DHODH inhibitor	370	-- ^c^
Lithium (chloride)	LTH	D	Mood stabilizer	1000	20,000
Methotrexate	MTX	D/X	Antineoplastic, dihydrofolate reductase inhibitor	1	20
Methylmercury	MEM	n/a	Bioaccumulative environmental toxicant, hypothesized ROS production	0.020	0.4
Panobinostat	PAN	n/a, (D)	Antineoplastic agent, HDAC inhibitor	0.06	1.2
Paroxetine	PAX	D	Antidepressant, SSR inhibitor	1.2	24
Phenytoin	PHE	D	Anticonvulsant, voltage-gated sodium channel blocker	20	--- ^c^
Retinol ^d^	RET	n/a	Vitamin and retinoid	-- ^d^	20 ^d^
Teriflunomide	TER	X	Anti-inflammatory agent, DHODH inhibitor	370	--- ^c^
Thalidomide	THD	X	Antiangiogenic	3.9	78
Trichostatin A	TSA	n/a	Antifungal antibiotic, HDAC inhibitor	0.01	0.2
Valproic acid	VPA	D, X	Anticonvulsant, voltage-gated sodium channel blocker, antifolate agent, HDAC inhibitor	600	1.67-fold C_max_: 1000 ^c^
Vinblastine	VIN	D	Antimitotic agent, affects microtubule dynamics	0.0247	0.494
Vismodegib	VIS	X	Antineoplastic agent, hedgehog pathway inhibitor	20	-- ^c^
Vorinostat	VST	D	Antineoplastic agent, HDAC inhibitor	3	60

**^a^** U.S. Food and Drug Administration (FDA) and Australian Therapeutic Goods Administration (TGA) pregnancy categories: A = compounds are safe to use during pregnancy, proven by well-controlled studies in humans or abundant data from pregnant women; B = compounds are considered to be safe, but they lack sufficient human data; C and D = compounds showed little or some evidence of teratogenicity in humans or animals; X = compounds with known teratogenic activity in humans or with a suspected high teratogenic potential based on animal experiments; n/a = not available; **^b^** maximal plasma or blood concentration after administration of therapeutic compound dose; **^c^** Carbamazepine and VPA were tested at 10-fold and 1.67-fold C_max_, respectively, instead of 20-fold C_max_; leflunomide, phenytoin, teriflunomide, and vismodegib were only tested at 1-fold C_max_ due to limited solubility; **^d^** Retinol was considered as a non-teratogen at 1-fold C_max_ and as a teratogen at 20-fold C_max_. Rationale is given in the supplemental information.

**Table 2:** Cytotoxicity and number of significantly deregulated probe sets in compound-exposed cells in the UKN1 test.

Compounds	Abbreviation	Cytotoxicity ^b^		Number of Up-/Downregulated Probe Sets ^c^			
				1-Fold C_max_^a^		20-Fold C_max_^a^	
		1-Fold C_max_^a^	20-Fold C_max_^a^	Up	Down	Up	Down
**Non-teratogens**							
Ampicillin	AMP	No	No	0	0	0	0
Ascorbic acid	ASC	No	No	0	0	0	0
Buspirone	BSP	No	No	0	0	0	0
Chlorpheniramine	CPA	No	No	0	0	0	0
Dextromethorphan	DEX	No	No	0	0	0	0
Diphenhydramine	DPH	No	No	0	0	0	0
Doxylamine	DOA	No	No	0	0	0	0
Famotidine	FAM	No	No	0	0	0	0
Folic acid	FOA	No	No	0	0	0	0
Levothyroxine	LEV	No	No	18	20	0	0
Liothyronine	LIO	No	No	0	0	27	57
Magnesium chloride	MAG	No	No	0	0	13	3
Methicillin	MET	No	No	0	0	0	2
Ranitidine	RAN	No	No	0	0	0	0
Retinol ^e^	RET	No	-- ^e^	0 ^e^	0 ^e^	-- ^e^	-- ^e^
Sucralose	SUC	No	No	0	0	0	0
**Teratogens**							
9-cis-retinoic acid	9RA	No	No	1956	1785	2426	1887
Acitretin	ACI	No	No	1803	1604	2309	1795
Actinomycin D	ACD	Yes	Yes	toxic	toxic	toxic	toxic
Atorvastatin	ATO	Yes	Yes	toxic	toxic	toxic	toxic
Carbamazepine	CMZ	No	No ^d^	0	0	910 ^d^	591 ^d^
Doxorubicin	DXR	Yes	Yes	toxic	toxic	toxic	toxic
Entinostat	ENT	No	Yes	48	30	toxic	toxic
Favipiravir	FPV	No	No	0	0	1551	1290
Isotretinoin	ISO	No	Yes	1029	703	toxic	toxic
Leflunomide	LFL	No	--- ^d^	614	415	--- ^d^	--- ^d^
Lithium chloride	LTH	No	Yes	0	0	toxic	toxic
Methotrexate	MTX	No	No	435	209	687	622
Methylmercury	MEM	No	No	0	0	0	0
Panobinostat	PAN	Yes	Yes	toxic	toxic	toxic	toxic
Paroxetine	PAX	No	Yes	0	0	toxic	toxic
Phenytoin	PHE	No	--- ^d^	0	0	--- ^d^	--- ^d^
Retinol ^e^	RET	-- ^e^	No	-- ^e^	-- ^e^	1032 ^e^	936 ^e^
Teriflunomide	TER	No	--- ^d^	1829	1394	--- ^d^	--- ^d^
Thalidomide	THD	No	No	41	21	0	0
Trichostatin A	TSA	No	Yes	4	0	toxic	toxic
Valproic acid	VPA	No	No ^d^	630	364	878 ^d^	685 ^d^
Vinblastine	VIN	Yes	Yes	toxic	toxic	toxic	toxic
Vismodegib	VIS	No	--- ^d^	0	0	--- ^d^	--- ^d^
Vorinostat	VST	Yes	Yes	toxic	toxic	toxic	toxic

**^a^** Maximal plasma or blood concentrations after administration of therapeutic compound dose; **^b^** Yes, if the compound was highly cytotoxic; no, if the compound showed no cytotoxicity; **^c^** Gene array-probe sets that were deregulated with an FDR-adjusted *p*-value < 0.05 and an absolute fold-change > 2 compared to untreated control cells; **^d^** Carbamazepine and VPA were tested at 10-fold and 1.67-fold C_max_, respectively, instead of 20-fold C_max_; leflunomide, phenytoin, teriflunomide, and vismodegib were only tested at 1-fold C_max_ due to limited solubility; **^e^** Retinol was considered as a non-teratogen at 1-fold C_max_ and as a teratogen at 20-fold C_max_. Rationale is given in the supplemental information.

**Table 3:** Performance metrics of the classification procedures in the UKN1 test, the UKK2 test, and the combination ‘mean’.

			1-Fold C_max_				20-Fold C_max_^a^			
Test	Data	Procedure	AUC	Accuracy	Sensitivity	Specificity	AUC	Accuracy	Sensitivity	Specificity
UKN1	Cytotoxicity		0.63	0.56	0.26	1	0.73	0.67	0.46	1
	Gene expression	SPS	0.78	0.64	0.43	0.94	0.82	0.62	0.38	1
		Top-1000	0.84	0.69	0.70	0.69	0.90	0.59	0.46	0.8
	Cytotoxicity and gene expression	SPS	0.84	0.79	0.70	0.94	**0.90**	**0.90**	**0.83**	**1**
		Top-1000	0.88	0.85	0.96	0.69	**0.95**	**0.87**	**0.92**	**0.8**
		RT-qPCR (SPS-like)	Not calculated	0.74	0.57	1	Not calculated	**0.90**	**0.83**	**1**
		RT-qPCR (Top-1000-like)	0.76	0.77	0.74	0.81	**0.91**	**0.87**	**0.88**	**0.87**
UKK2	Cytotoxicity		0.61	0.54	0.22	1	0.63	0.54	0.25	1
	Gene expression	SPS	0.87	0.77	0.61	1	0.83	0.69	0.58	0.87
		Top-1000	0.93	0.79	0.74	0.88	0.92	0.77	0.71	0.87
	Cytotoxicity and gene expression	SPS	**0.9**	**0.9**	**0.83**	**1**	0.87	0.85	0.83	0.87
		Top-1000	**0.95**	**0.92**	**0.96**	**0.88**	0.94	0.92	0.96	0.87
Combination ‘mean’	Cytotoxicity		0.63	0.56	0.26	1	0.73	0.67	0.46	1
	Gene expression	SPS	0.89	0.77	0.61	1	0.84	0.64	0.42	1
		Top-1000	0.94	0.79	0.74	0.88	0.96	0.79	0.75	0.87
	Cytotoxicity and gene expression	SPS	0.92	0.92	0.87	1	**0.91**	**0.92**	**0.88**	**1**
		Top-1000	0.95	0.92	0.96	0.88	**0.98**	**0.95**	**1**	**0.87**

Cytotoxicity = Only cytotoxicity data were considered for the calculation of the metrics, i.e., cytotoxic conditions were considered as positive and non-cytotoxic conditions as negative test results. Gene expression = only gene expression data were considered for the calculation of the metrics. Cytotoxicity and gene expression = all data for cytotoxicity, as well as for gene expression, were considered for the calculation of the metrics. AUC (area-under-curve) = for each possible cut-off used as threshold, predictions were made for each of the conditions based on which sensitivity and specificity were calculated. The ROC-curve (receiver operator characteristic) was obtained by plotting all pairs of (1-specificity) and sensitivity against each other. The AUC was determined as the area under this ROC-curve. Accuracy = ratio of correct predictions ((true negatives and positives)/(true and false negatives and positives)) (Supplementary Table S1). Sensitivity = ratio of detected teratogens (true positives/(false negatives + true positives)) (Supplementary Table S1). Specificity = ratio of detected non-teratogens (true negatives/(true negatives + false positives)) (Supplementary Table S1. ^a^ Including 10-fold C_max_ carbamazepine, 1.67-fold C_max_ VPA, and 1-fold C_max_ samples of leflunomide, phenytoin, teriflunomide, and vismodegib. RT-qPCR metrics used 1-fold C_max_ results instead of 20-fold C_max_ results for levothyroxine. Bold = best metrices for each test for the SPS- and top-1000-procedure and RT-qPCR.

**Table 4:** Classification of the in vitro results of the test conditions with the highest accuracies.

Compounds	Abbreviation	SPS-Procedure			Top-1000-Procedure			RT-qPCR (SPS-Like)	RT-qPCR (Top-1000-Like)
		UKN1 20-Fold Cmax ^a^	UKK2 1-Fold Cmax ^a^	Mean 20-Fold Cmax ^a^	UKN1 20-Fold Cmax ^a^	UKK2 1-Fold Cmax ^a^	Mean 20-Fold Cmax ^a^	UKN1 20-Fold Cmax ^a^	UKN1 20-Fold Cmax ^a^
**Non-teratogens**									
Ampicillin	AMP	TN	TN	TN	TN	TN	TN	TN	TN
Ascorbic acid	ASC	TN	TN	TN	FP	TN	TN	TN	FP
Buspirone	BSP	TN	TN	TN	TN	TN	TN	TN	TN
Chlorpheniramine	CPA	TN	TN	TN	TN	TN	TN	TN	TN
Dextromethorphan	DEX	TN	TN	TN	TN	TN	TN	TN	TN
Diphenhydramine	DPH	TN	TN	TN	FP	FP	FP	TN	TN
Doxylamine	DOA	TN	TN	TN	TN	TN	TN	TN	TN
Famotidine	FAM	TN	TN	TN	TN	TN	TN	TN	TN
Folic acid	FOA	TN	TN	TN	TN	TN	TN	TN	TN
Levothyroxine	LEV	TN	TN	TN	TN	TN	TN	TN ^e^	TN ^e^
Liothyronine	LIO	TN	TN	TN	TN	TN	TN	TN	FP
Magnesium chloride	MAG	TN	TN	TN	TN	TN	TN	TN	TN
Methicillin	MET	TN	TN	TN	TN	TN	TN	TN	TN
Ranitidine	RAN	TN	TN	TN	TN	TN	TN	TN	TN
Retinol ^d^	RET	-- ^d^	TN ^d^	-- ^d^	-- ^d^	TN ^d^	-- ^d^	-- ^d^	-- ^d^
Sucralose	SUC	TN	TN	TN	FP	FP	FP	TN	TN
**Teratogens**									
9-cis-retinoic acid	9RA	TP	TP	TP	TP	TP	TP	TP	TP
Acitretin	ACI	TP	TP	TP	TP	TP	TP	TP	TP
Actinomycin D	ACD	TP	TP	TP	TP	TP	TP	TP	TP
Atorvastatin	ATO	TP	FN	TP	TP	FN	TP	TP	TP
Carbamazepine	CMZ	TP ^b^	TP	TP ^b^	TP ^b^	TP	TP ^b^	TP ^b^	TP ^b^
Doxorubicin	DXR	TP	TP	TP	TP	TP	TP	TP	TP
Entinostat	ENT	TP	TP	TP	TP	TP	TP	TP	TP
Favipiravir	FPV	TP	FN	TP	TP	TP	TP	TP	TP
Isotretinoin	ISO	TP	TP	TP	TP	TP	TP	TP	TP
Leflunomide	LFL	TP ^c^	TP	TP ^c^	TP ^c^	TP	TP ^c^	TP ^c^	TP ^c^
Lithium chloride	LTH	TP	TP	TP	TP	TP	TP	TP	TP
Methotrexate	MTX	TP	TP	TP	TP	TP	TP	TP	TP
Methylmercury	MEM	FN	TP	FN	TP	TP	TP	FN	FN
Panobinostat	PAN	TP	TP	TP	TP	TP	TP	TP	TP
Paroxetine	PAX	TP	TP	TP	TP	TP	TP	TP	TP
Phenytoin	PHE	FN ^c^	FN	FN ^c^	TP ^c^	TP	TP ^c^	FN ^c^	FN ^c^
Retinol ^d^	RET	TP ^d^	-- ^d^	TP ^d^	TP ^d^	-- ^d^	TP ^d^	TP ^d^	TP ^d^
Teriflunomide	TER	TP ^c^	TP	TP ^c^	TP ^c^	TP	TP ^c^	TP ^c^	TP ^c^
Thalidomide	THD	FN	TP	TP	FN	TP	TP	FN	TP
Trichostatin A	TSA	TP	TP	TP	TP	TP	TP	TP	TP
Valproic acid	VPA	TP ^b^	TP	TP ^b^	TP ^b^	TP	TP ^b^	TP ^b^	TP ^b^
Vinblastine	VIN	TP	TP	TP	TP	TP	TP	TP	TP
Vismodegib	VIS	FN ^c^	FN	FN ^c^	FN ^c^	TP	TP ^c^	FN ^c^	FN ^c^
Vorinostat	VST	TP	TP	TP	TP	TP	TP	TP	TP

TN = True Negative; FN = False Negative; FP = False Positive; TP = True Positive. Grey = Misclassifications. ^a^ Maximal plasma or blood concentrations after administration of therapeutic compound dose; ^b^ Carbamazepine and VPA were tested at 10-fold and 1.67-fold C_max_, respectively, instead of 20-fold C_max_; ^c^ Due to a limited solubility of LFL, PHE, TER and VIS, the highest tested concentration was 1-fold C_max_; here, SPS-numbers and predicted probabilities for teratogenicity obtained at 1-fold C_max_ were used to classify the compounds compared to the 20-fold C_max_ threshold. See methods and discussion for further details; ^d^ Retinol was considered as a non-teratogen at 1-fold C_max_ and as a teratogen at 20-fold C_max_. Rationale is given in the supplemental information; ^e^ RT-qPCR measurements were not available for levothyroxine at 20-fold C_max_; instead, the RT-qPCR results of levothyroxine at 1-fold C_max_ were used to classify levothyroxine at 20-fold C_max_.

## Data Availability

The raw data generated with the gene arrays during the current study are accessible in the Gene Expression Omnibus (GEO) database under GSE209962; additional data from gene array data analyses and RT-qPCR data are included in this published article and its Supplementary Information Files.

## Supplementary Materials

The following supporting information can be downloaded at: https://www.mdpi.com/article/10.3390/cells11213404/s1, Teratogenicity of high-dosed retinol; SOP for the UKN1 protocol; Table S1: Classification of the in vitro test results; Table S2: Predicted probabilities for teratogenicity in the UKN1 test, the UKK2 test, the test combinations, and the RT-qPCR test; Table S3: Performance metrics of the test combinations ‘min’ and ‘max’; Table S4: Cytotoxicity and number of significantly deregulated probe sets in the test combinations; Figure S1–S5: Biological interpretation of genes differentially expressed after exposure of hiPSC to teratogens in the UKN1 test; Figure S6–S10: Biological interpretation and comparison of genes differentially expressed in the UKN1 and UKK2 test after exposure of hiPSC to teratogens; Figure S11: Classification of the teratogenic and non-teratogenic compounds by (A and B) combinations of the SPS-procedures and (C and D) the top-1000-procedures of the UKN1 and UKK2 test; Figure S12: Correlation plot of substance-induced gene expression changes in UKN1 measured in gene arrays and RT-qPCR; Figure S13: Classification of the teratogenic and non-teratogenic compounds based on the expression of 7-top-genes of the UKN1 test measured in RT-qPCR; Figure S14–S20: Expression changes of selected top genes in UKN1 samples relative to controls obtained by gene array and RT-qPCR; Supplementary Excel-File S1 ‘UKN1_Top genes’; Supplementary Excel-File S2 ‘UKN1_KEGG pathways;’ Supplementary Excel-File S3 ‘UKN1_GO groups;’ Supplementary Excel-File S4 ‘UKN1 vs. UKK2_KEGG pathways;’ Supplementary Excel-File S5 ‘UKN1 vs. UKK2_GO groups;’ Supplementary Excel-File S6 ‘UKN1 vs. UKK2_Top genes;’ Supplemental Excel-File S7 ‘Biomarker expression in gene array and RT-qPCR;’ and volcano plots of all substance-induced gene expression changes in UKN1. References [47,48,49] are mentioned in Supplementary Materials.

## Abbreviations

hiPSC	Human induced pluripotent stem cell
NEP	Neuroepithelial precursors
C_max_	Maximal plasma concentration
PS	Probe set
SPS	Significant probe set
AUC	Area-under-the-(receiver operator characteristic)-curve
GO	Gene Ontology
KEGG	Kyoto Encyclopedia of Genes and Genomes
