# Supplementary material for: High Accuracy Classification of Developmental Toxicants by In Vitro Tests of Human Neuroepithelial and Cardiomyoblast Differentiation
Source: Cells. 2022 Oct 27;11(21):3404. doi: 10.3390/cells11213404 (PMC9653768; doi:10.3390/cells11213404)
Supplement: Supplementary file 1 [file cells-11-03404-s001.zip › Supporting information 1.pdf]

SUPPORTING INFORMATION 1

High accuracy classification of developmental toxicants by in vitro tests of human neuroepithelial and cardiomyoblast differentiation

Florian Seidel [1,*,‡], Anna Cherianidou [2,‡], Franziska Kappenberg [3,‡], Miriam Marta [1], Nadine Dreser [4], Jonathan Blum [4], Tanja Waldmann [5], Nils Blüthgen [6,7], Johannes Meisig [6,7], Katrin Madjar [3], Margit Henry [2,8], Tamara Rotshteyn [2,8], Andreas Scholtz-Illigens [1], Rosemarie Marchan [1], Karolina Edlund [1], Marcel Leist [4,†], Jörg Rahnenführer [3,†], Agapios Sachinidis [2,8,†] and Jan G. Hengstler [1,*,†]

[1] Leibniz Research Centre for Working Environment and Human Factors (IfADo), Technical University of Dortmund, Ardeystrasse 67, 44139 Dortmund, Germany

[2] Working Group Sachinidis, Center for Physiology, Faculty of Medicine and University Hospital Cologne, University of Cologne, Robert-Koch-Str. 39, 50931 Cologne, Germany

[3] Department of Statistics, TU Dortmund University, Vogelpothsweg 87, 44227 Dortmund, Germany

[4] In Vitro Toxicology and Biomedicine, Department of Biology, University of Konstanz, Universitätsstr. 10, 78454 Konstanz, Germany

[5] Department of Advanced Cell Systems, trenzyme GmbH, Byk-Gulden-Str. 2, 78467 Konstanz, Germany

[6] Institute of Pathology, Charité-Universitätsmedizin Berlin, Chariteplatz 1, 10117 Berlin, Germany

[7] IRI Life Sciences, Humboldt Universität zu Berlin, Philippstraße 13, Haus 18, 10115 Berlin, Germany

[8] Center for Molecular Medicine Cologne (CMMC), University of Cologne, 50931 Cologne, Germany

* Correspondence: seidelf@ifado.de (F.S.); hengstler@ifado.de (J.G.H.)

† These authors contributed equally to this work.

‡ These authors contributed equally to this work.

Table of Contents

Teratogenicity of high-dosed retinol 3

SOP UKN1 protocol 4

Table S1 7

Table S2 9

Table S3 10

Table S4 11

Fig. S1-S5 12

Fig. S6-S10 18

Fig. S11 24

Fig. S12 25

Fig. S13 26

Fig. S14-S20 27

Teratogenicity of high-dosed retinol

The assessment for 1 µM retinol as a non-teratogenic concentration is based on blood concentrations reported for the general population, such as 0.67 – 1.0 µM [47] or cord blood concentrations of 0.7 – 1.3 µM [48]. The teratogenic concentration (20 µM) is based on a published evaluation that a dose of 277 mg retinol for a 50 kg pregnant woman would represent the lowest teratogenic intake if one assumes that humans are as sensitive as rabbits [49]. This assumption is conservative since rabbits were the most sensitive species to the teratogenicity of retinoids compared to rats, mice and hamsters [49]. It should be considered that relatively high doses of retinol used for pharmacokinetic tests in humans (150 mg, oral dose in oil) caused only a moderate increase of the retinol concentration in blood that was clearly below 20 µM [49]. Thus, the assumption that 20 µM retinol in blood represents a teratogenic concentration is still affected with some uncertainty, because teratogenicity has not yet been directly demonstrated in humans; nevertheless, it can be expected that 20 µM in human serum represents an extremely high concentration of retinol that most probably exceeds the C_{max} after teratogenic doses, if humans are as susceptible to retinol-induced teratogenicity as rabbits.

References

47. Sklan, D.; Shalit, I.; Lasebnik, N.; Spirer, Z.; Weisman, Y. (1985): Retinol transport proteins and concentrations in human amniotic fluid, placenta, and fetal and maternal sera. In The British journal of nutrition 54 (3), pp. 577–583. DOI: 10.1079/bjn19850144.

48. Manolescu, Daniel C.; El-Kares, Reyhan; Lakhal-Chaieb, Lajmi; Montpetit, Alexandre; Bhat, Pangala V.; Goodyer, Paul (2010): Newborn serum retinoic acid level is associated with variants of genes in the retinol metabolism pathway. In Pediatric research 67 (6), pp. 598–602. DOI: 10.1203/PDR.0b013e3181dcf18a.

49. Blomhoff, Rune (2001): Vitamin A and Carotenoid Toxicity. In Food Nutr Bull 22 (3), pp. 320–334. DOI: 10.1177/156482650102200309.

SOP UKN1 protocol

(Version September 12th, 2022)

Developmental toxicity testing with hiPSCs differentiating to neuroectodermal precursor cells

Material:

1. **Equipment:**
   - Laminar airflow cabinet
   - Incubator (37 °C, humidified atmosphere of air containing 5 % CO2)
   - Thermostated water bath (37 °C)
   - Bright field microscope
   - Sonicator
   - Tissue culture plate, 12-Well (Sarstedt, Cat. 833921)

2. **Culture medium for hiPSC maintenance:**
   - Cellartis® DEF-CS™ 500 Culture System (Takara Bio, Cat. Y30010), includes:
     i. DEF-CS basal medium
     ii. GF-1, GF-2, GF-3 (growth factors)
     iii. Coat-1 stock solution (extracellular matrix protein)
   - Essential 8™ (E8) medium (ThermoFisher Scientific, Cat. A1517001); includes:
     i. Essential 8 basal medium
     ii. Essential 8 supplement, 50×

3. **Culture medium for hiPSC differentiation:**
   - Essential 6™ (E6) (ThermoFisher Scientific, Cat. A1516401)
   - KSR-S:
     i. Knockout DMEM (ThermoFisher Scientific, Cat. 10829-018)
     ii. 15% Knockout serum replacement (ThermoFisher Scientific, Cat. 10828-010)
     iii. 1× GlutaMax (ThermoFisher Scientific, Germany, Cat. 35050038)
     iv. 1× MEM non-essential amino acids (ThermoFisher Scientific, Cat. 11140035)
     v. 50 µM beta-mercaptoethanol (ThermoFisher Scientific, Cat. 31350010)
   - N2-S:
     i. DMEM/F12 medium, no glutamine (ThermoFisher Scientific, Cat. 21331-020)
     ii. 1× GlutaMax (ThermoFisher Scientific, Cat. 35050038)
     iii. 0.1 mg/ml apotransferrin (Sigma Aldrich, Cat. T2036)
     iv. 1.55 mg/ml glucose (Sigma Aldrich, Cat. 49159)
     v. 25 µg/ml insulin (Sigma Aldrich, Cat. I9278)
     vi. 100 µM putrescine (Sigma Aldrich, Cat. P5780)
     vii. 30 nM selenium (Sigma Aldrich, Cat. S5261)
     viii. 20 nM progesterone (Sigma Aldrich, Cat. P7556)

4. **Cell culture additives and reagents:**
   - DPBS (+/+) (ThermoFisher Scientific, Cat. 14040091)
   - DPBS (-/-) (ThermoFisher Scientific, Cat. 14190094)

- Biolaminin 521 LN (BioLamina, Cat. LN521-05)
- Vitronectin (ThermoFisher Scientific, Cat. A31804)
- Y-27632 (Cell Guidance Systems, Cat. SM02)
- SB431542 (Cell Guidance Systems, Cat. SM33)
- Dorsomorphin (Cell Guidance Systems, Cat. SM03)
- Noggin (R&D Systems, Cat. 6057-NG)
- Dimethyl sulfoxide (DMSO) (Carl Roth, Cat. A994.1)

5. **RNA-Isolation-Kit:** ExtractMe Total RNA-Kit (Blirt, EM09.1)

6. **Test compounds**

Methods:

1. **Preparation of cell culture additives and reagents:**

- Putrescine stock solution: 161 mg putrescine dihydrochloride is solved in 1 ml dH_2O at a concentration of 1 M and filtered by an 0.2 µM syringe filter. Aliquots à 55 µl. Freeze aliquots at -80 °C.
- Selenium stock solution: 86.5 µg sodium selenite is solved in 1 ml dH_2O at a concentration of 500 µM and filtered by an 0.2 µM syringe filter. Aliquots à 33 µl. Freeze aliquots at -80 °C.
- Progesterone stock solution: 425 µg is solved in 1 ml dH_2O at a concentration of 100 µM and filtered by an 0.2 µM syringe filter. Aliquots à 110 µl. Freeze aliquots at -80 °C.
- Y-27632: Solve sterile 10 mg lyophilizate in 3 ml dH_2O at a concentration of 10 mM. Aliquots à 150 µl. Freeze aliquots at -80 °C.
- SB431542: Solve sterile 10 mg lyophilizate in 1.2 ml absolute (100 %) ethanol at a concentration of 21.6 mM. Aliquots à 200 µl. Freeze aliquots at -80 °C.
- Dorsomorphin: Solve sterile 1 mg lyophilizate in 2 ml dH_2O at a concentration of 1 mM. Aliquots à 100 µl. Freeze aliquots at -80 °C.
- Noggin: Solve sterile 100 µg lyophilizate in 400 µl 0.1 % human serum albumin/DPBS at a concentration of ca. 250 µg/ml. Aliquots à 40 µl. Freeze aliquots at -80 °C.

2. **Preparation of cell culture media and coated tissue culture plates**

- KSR-S and N2-S are prepared as mentioned above. N2-S is further filtrated with a 0.2 µm filter bottle.
- DEF-CS basal medium is spiked with 1:333 GF-1 and 1:1000 GF-2. If cells will be passaged, GF-3 is added at a concentration of 1:1000
- Essential 8 basal medium is supplemented with 1× Essential 8 supplement. If cells will be passaged, Y-27632 is added at a concentration of 10 µM
- Tissue culture plates are coated with either Coat-1, biolaminin 521 or vitronectin. Therefore, stock solutions were diluted to 5-10 µg/ml in DPBS (+/+). Tissue culture plates are incubated with 100 µl coating solution per cm² for 30 min at 37 °C. The solution is aspirated immediately before use of the culture plate.

3. Neuroepithelial differentiation of hiPSCs

An overview of the differentiation protocol is given in the figure below. Human induced pluripotent stem cells (hiPSCs) are seeded in 1 ml pluripotent stem cell (PSC) medium (DEF-CS or Essential 8) per well on coated 12-well-plates at a density of 12,000 - 24,000 cells/cm² on day -3. A rho-kinase inhibitor (ROCKi) was added (GF-3 or Y27632). Up to one million cells from the cell suspension are collected for RNA isolation in 600 µl Rlys Buffer from the ExtractMe RNA extraction kit spiked with 20 µl antifoam reagent. To improve lysis, the lysate can be pipetted up and down multiple times without creating foam. Lysate is sonicated on ice and stored at -80 °C for up to one month prior to RNA isolation.
On day -2 and day -1, the PSC medium is refreshed.
On day 0, cells should be 80 % confluent. Cells are washed once with DPBS (-/-) before addition of 1 ml/well differentiation medium (KSR-S or Essential 6) which is spiked with 21.6 µM SB431542, 0.64 µM dorsomorphin, 35 ng/ml noggin and 0.1% DMSO to induce neural differentiation. At the same time, cells are incubated with test compounds as well as with the vehicle alone (0.1% DMSO).
On days 1 and 2, medium is refreshed.
On day 4, cells are washed once with DPBS (-/-). Then, 1 ml/well mixed medium of 75% differentiation medium / 25% N2-S and same concentrations of SB431542, dorsomorphin and noggin as above is added.
On day 6, cells are washed once with DPBS (-/-) before they are collected for RNA extraction in 350 µl/well ExtractMe lysis buffer spiked with 12 µl antifoam reagent. Each well is rinsed several times to collect as much cells as possible. To improve lysis, the lysate can be pipetted up and down multiple times without creating foam. The cell lysate is sonicated and frozen at -80 °C for up to one month prior to RNA isolation.

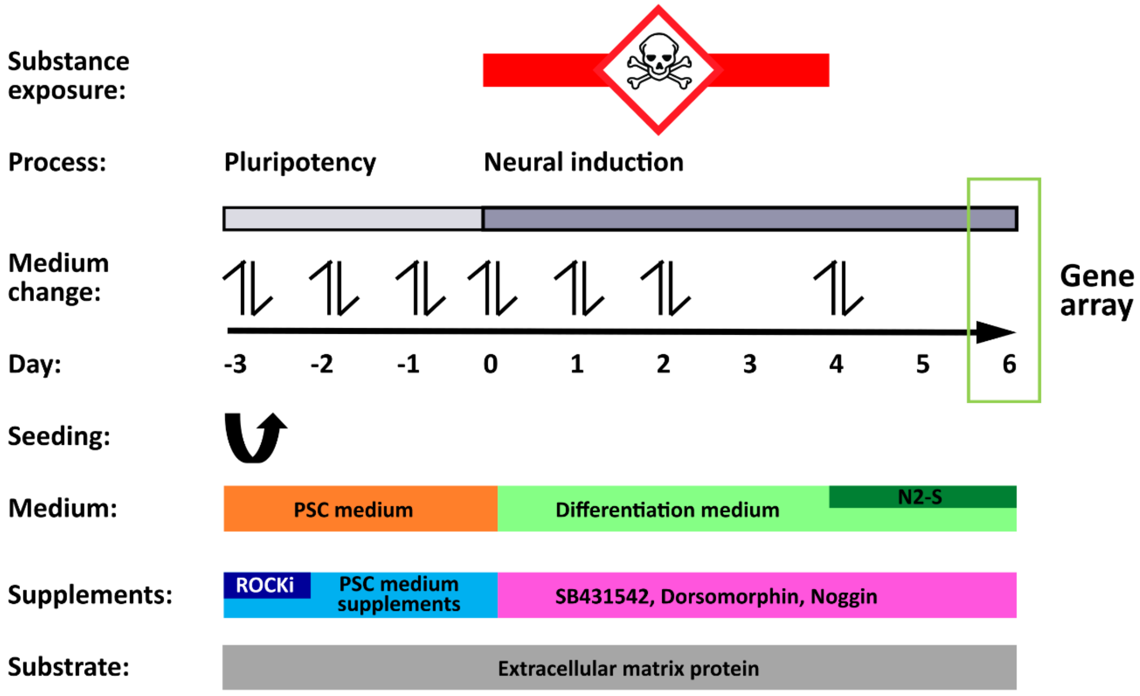

Table S1 Classification of the in vitro test results (when cytotoxicity and gene expression were considered, with respect to the particular thresholds)

	SPS-procedure										Top-1,000-procedure										RT-qPCR (SPS-like-procedure)		RT-qPCR (Top-1,000-like-procedure)	
**Test**	**UKN1**		**UKK2**		**Min**		**Mean**		**Max**		**UKN1**		**UKK2**		**Min**		**Mean**		**Max**		**UKN1**			
**C_{max}[a] / Compounds**	**1-fold**	**20-fold**	**1-fold**	**20-fold**	**1-fold**	**20-fold**	**1-fold**	**20-fold**	**1-fold**	**20-fold**	**1-fold**	**20-fold**	**1-fold**	**20-fold**	**1-fold**	**20-fold**	**1-fold**	**20-fold**	**1-fold**	**20-fold**	**1-fold**	**20-fold**	**1-fold**	**20-fold**
**Non-teratogens**																								
Ampicillin	TN	TN	TN	TN	TN	TN	TN	TN	TN	TN	TN	TN	TN	TN	TN	TN	TN	TN	TN	TN	TN	TN	TN	TN
Ascorbic acid	TN	TN	TN	FP	TN	TN	TN	TN	TN	TN	TN	FP	TN	TN	TN	TN	TN	TN	TN	FP	TN	TN	FP	FP
Buspirone	TN	TN	TN	TN	TN	TN	TN	TN	TN	TN	TN	TN	TN	TN	TN	TN	TN	TN	TN	TN	TN	TN	TN	TN
Chlorpheniramine	TN	TN	TN	TN	TN	TN	TN	TN	TN	TN	TN	TN	TN	TN	TN	TN	TN	TN	TN	TN	TN	TN	TN	TN
Dextromethorphan	TN	TN	TN	TN	TN	TN	TN	TN	TN	TN	FP	TN	TN	TN	TN	TN	TN	TN	TN	TN	TN	TN	TN	TN
Diphenhydramine	TN	TN	TN	TN	TN	TN	TN	TN	TN	TN	FP	FP	FP	FP	FP	FP	FP	FP	FP	TN	TN	TN	TN	TN
Doxylamine	TN	TN	TN	TN	TN	TN	TN	TN	TN	TN	TN	TN	TN	TN	TN	TN	TN	TN	TN	TN	TN	TN	TN	TN
Famotidine	TN	TN	TN	TN	TN	TN	TN	TN	TN	TN	TN	TN	TN	TN	TN	TN	TN	TN	TN	TN	TN	TN	TN	TN
Folic acid	TN	TN	TN	TN	TN	TN	TN	TN	TN	TN	TN	TN	TN	TN	TN	TN	TN	TN	TN	TN	TN	TN	TN	TN
Levothyroxine	FP	TN	TN	TN	TN	TN	TN	TN	TN	TN	TN	TN	TN	TN	TN	TN	TN	TN	TN	TN	TN	TN[e]	FP	TN[e]
Liothyronine	TN	TN	TN	TN	TN	FP	TN	TN	TN	TN	TN	TN	TN	TN	TN	TN	TN	TN	TN	TN	TN	TN	FP	FP
Magnesium chloride	TN	TN	TN	FP	TN	TN	TN	TN	TN	TN	TN	TN	TN	TN	TN	TN	TN	TN	TN	TN	TN	TN	TN	TN
Methicillin	TN	TN	TN	TN	TN	TN	TN	TN	TN	TN	TN	TN	TN	TN	TN	TN	TN	TN	TN	TN	TN	TN	TN	TN
Ranitidine	TN	TN	TN	TN	TN	TN	TN	TN	TN	TN	FP	TN	TN	TN	TN	TN	TN	TN	TN	TN	TN	TN	TN	TN
Retinol[d]	TN[d]	--[d]	TN[d]	--[d]	TN[d]	--[d]	TN[d]	--[d]	TN[d]	--[d]	FP[d]	--[d]	TN[d]	--[d]	FP[d]	--[d]	TN[d]	--[d]	TN[d]	--[d]	TN	--[d]	TN[d]	--[d]
Sucralose	TN	TN	TN	TN	TN	TN	TN	TN	TN	TN	FP	FP	FP	FP	FP	FP	FP	FP	FP	FP	TN	TN	TN	TN
**Teratogens**																								
9-cis-retinoic acid	TP	TP	TP	TP	TP	TP	TP	TP	TP	TP	TP	TP	TP	TP	TP	TP	TP	TP	TP	TP	TP	TP	TP	TP
Acitretin	TP	TP	TP	TP	TP	TP	TP	TP	TP	TP	TP	TP	TP	TP	TP	TP	TP	TP	TP	TP	TP	TP	TP	TP
Actinomycin D	TP	TP	TP	TP	TP	TP	TP	TP	TP	TP	TP	TP	TP	TP	TP	TP	TP	TP	TP	TP	TP	TP	TP	TP
Atorvastatin	TP	TP	FN	TP	TP	TP	TP	TP	TP	TP	TP	TP	FN	FN	FN	FN	TP	TP	TP	TP	TP	TP	TP	TP
Carbamazepine	FN	TP[b]	TP	TP	FN	TP[b]	TP	TP[b]	TP	TP[b]	TP	TP[b]	TP	TP	TP	TP[b]	TP	TP[b]	TP	TP[b]	FN	TP[b]	FN	TP[b]
Doxorubicin	TP	TP	TP	TP	TP	TP	TP	TP	TP	TP	TP	TP	TP	TP	TP	TP	TP	TP	TP	TP	TP	TP	TP	TP
Entinostat	TP	TP	TP	TP	TP	TP	TP	TP	TP	TP	TP	TP	TP	TP	TP	TP	TP	TP	TP	TP	FN	TP	FN	TP
Favipiravir	FN	TP	FN	TP	FN	TP	FN	TP	FN	TP	TP	TP	TP	TP	TP	TP	TP	TP	TP	TP	FN	TP	TP	TP
Isotretinoin	TP	TP	TP	TP	TP	TP	TP	TP	TP	TP	TP	TP	TP	TP	TP	TP	TP	TP	TP	TP	TP	TP	TP	TP
Leflunomide	TP	TP[c]	TP	TP[c]	TP	TP[c]	TP	TP[c]	TP	TP[c]	TP	TP[c]	TP	TP[c]	TP	TP[c]	TP	TP[c]	TP	TP[c]	TP	TP[c]	TP	TP[c]

	SPS-procedure										Top-1,000-procedure										RT-qPCR (SPS-like-procedure)		RT-qPCR (Top-1,000-like-procedure)	
**Test**	**UKN1**		**UKK2**		**Min**		**Mean**		**Max**		**UKN1**		**UKK2**		**Min**		**Mean**		**Max**		**UKN1**			
**C_{max}[a] / Compounds**	**1-fold**	**20-fold**	**1-fold**	**20-fold**	**1-fold**	**20-fold**	**1-fold**	**20-fold**	**1-fold**	**20-fold**	**1-fold**	**20-fold**	**1-fold**	**20-fold**	**1-fold**	**20-fold**	**1-fold**	**20-fold**	**1-fold**	**20-fold**	**1-fold**	**20-fold**	**1-fold**	**20-fold**
Lithium chloride	FN	TP	TP	TP	FN	TP	TP	TP	TP	TP	TP	TP	TP	TP	TP	TP	TP	TP	TP	TP	FN	TP	FN	TP
Methotrexate	TP	TP	TP	TP	TP	TP	TP	TP	TP	TP	TP	TP	TP	TP	TP	TP	TP	TP	TP	TP	TP	TP	TP	TP
Methylmercury	FN	FN	TP	FN	FN	FN	TP	FN	TP	FN	TP	TP	TP	TP	TP	TP	TP	TP	TP	TP	FN	FN	FN	FN
Panobinostat	TP	TP	TP	TP	TP	TP	TP	TP	TP	TP	TP	TP	TP	TP	TP	TP	TP	TP	TP	TP	TP	TP	TP	TP
Paroxetine	FN	TP	TP	TP	FN	TP	TP	TP	TP	TP	FN	TP	TP	TP	FN	TP	FN	TP	FN	TP	FN	TP	FN	TP
Phenytoin	FN	FN[c]	FN	FN[c]	FN	FN[c]	FN	FN[c]	FN	FN[c]	TP	TP[c]	TP	TP	TP	TP[c]	TP	TP[c]	TP	TP[c]	FN	FN[c]	TP	FN[c]
Retinol[d]	--[d]	TP [d]	--[d]	FN [d]	--[d]	TP [d]	--[d]	TP [d]	--[d]	TP [d]	--[d]	TP [d]	--[d]	TP [d]	--[d]	TP [d]	--[d]	TP [d]	--[d]	TP [d]	--[d]	TP [d]	--[d]	TP [d]
Teriflunomide	TP	TP[c]	TP	TP[c]	TP	TP[c]	TP	TP[c]	TP	TP[c]	TP	TP[c]	TP	TP[c]	TP	TP[c]	TP	TP[c]	TP	TP[c]	TP	TP[c]	TP	TP[c]
Thalidomide	TP	FN	TP	TP	TP	FN	TP	TP	TP	TP	TP	FN	TP	TP	TP	TP	TP	TP	FN	FN	FN	FN	TP	TP
Trichostatin A	TP	TP	TP	TP	FN	TP	TP	TP	TP	TP	TP	TP	TP	TP	TP	TP	TP	TP	TP	TP	FN	TP	TP	TP
Valproic acid	TP	TP[b]	TP	TP	TP	TP[b]	TP	TP[b]	TP	TP[b]	TP	TP[b]	TP	TP	TP	TP[b]	TP	TP[b]	TP	TP[b]	TP	TP[b]	TP	TP[b]
Vinblastine	TP	TP	TP	TP	TP	TP	TP	TP	TP	TP	TP	TP	TP	TP	TP	TP	TP	TP	TP	TP	TP	TP	TP	TP
Vismodegib	FN	FN[c]	FN	FN[c]	FN	FN[c]	FN	FN[c]	FN	FN[c]	TP	FN[c]	TP	TP[c]	TP	TP[c]	TP	TP[c]	TP	TP[c]	FN	FN[c]	FN	FN[c]
Vorinostat	TP	TP	TP	TP	TP	TP	TP	TP	TP	TP	TP	TP	TP	TP	TP	TP	TP	TP	TP	TP	TP	TP	TP	TP

TN = True Negative; FN = False Negative; FP = False Positive; TP = True Positive

[a]Maximal plasma or blood concentrations after administration of therapeutic compound dose.

[b]Carbamazepine and VPA were tested at 10-fold and 1.67-fold C_{max}, respectively, instead of 20-fold C_{max}.

[c]Due to a limited solubility of LFL, PHE, TER and VIS, the highest tested concentration was 1-fold C_{max}; here, SPS-numbers and predicted probabilitites for teratogenicity obtained at 1-fold C_{max} were used to classify the compounds compared to the 20-fold C_{max} threshold. See methods and discussion for further details.

[d]Retinol was considered as a non-teratogen at 1-fold C_{max} and as a teratogen at 20-fold C_{max}. Rationale is given in the methods.

[e]RT-qPCR measurements were not available for levothyroxine at 20-fold C_{max}; instead, the RT-qPCR results of levothyroxine at 1-fold C_{max} were used to classify levothyroxine at 20-fold C_{max}.

Table S2 Predicted probabilities for teratogenicity in the UKN1 test, the UKK2 test, the test combinations and the RT-qPCR test

	Predicted probability for teratogenicity[b]											
Test	UKN1		UKK2		Min		Mean		Max		RT-qPCR	
C_{max}[a] / Compounds	1-fold	20-fold	1-fold	20-fold	1-fold	20-fold	1-fold	20-fold	1-fold	20-fold	1-fold	20-fold
**Non-teratogens**												
Ampicillin	0.10	0.05	0.10	0.00	0.10	0.00	0.10	0.03	0.10	0.05	0.22	0.30
Ascorbic acid	0.13	0.55	0.00	0.02	0.00	0.02	0.07	0.28	0.13	0.55	0.40	0.85
Buspirone	0.08	0.10	0.03	0.01	0.03	0.01	0.05	0.06	0.08	0.10	0.23	0.24
Chlorpheniramine	0.05	0.05	0.00	0.00	0.00	0.00	0.03	0.03	0.05	0.05	0.22	0.22
Dextromethorphan	0.31	0.36	0.01	0.03	0.01	0.03	0.16	0.19	0.31	0.36	0.24	0.37
Diphenhydramine	0.48	0.42	0.68	0.30	0.48	0.30	0.58	0.36	0.68	0.42	0.22	0.21
Doxylamine	0.17	0.18	0.04	0.00	0.04	0.00	0.10	0.09	0.17	0.18	0.23	0.29
Famotidine	0.11	0.16	0.11	0.01	0.11	0.01	0.11	0.08	0.11	0.16	0.23	0.22
Folic acid	0.10	0.09	0.00	0.00	0.00	0.00	0.05	0.05	0.10	0.09	0.23	0.20
Levothyroxine	0.13	0.15	0.00	0.00	0.01	0.03	0.07	0.09	0.13	0.15	0.37	--[e]
Liothyronine	0.12	0.30	0.01	0.03	0.00	0.00	0.06	0.15	0.12	0.30	0.46	0.59
Magnesium	0.10	0.14	0.00	0.05	0.00	0.05	0.05	0.09	0.10	0.14	0.22	0.30
Methicillin	0.09	0.09	0.01	0.01	0.01	0.01	0.05	0.05	0.09	0.09	0.21	0.27
Ranitidine	0.42	0.33	0.10	0.05	0.10	0.05	0.26	0.19	0.42	0.33	0.26	0.27
Retinol[d]	0.45[d]	--[d]	0.23[d]	--[d]	0.23[d]	--[d]	0.34[d]	--[d]	0.45[d]	--[d]	0.22	--[d]
Sucralose	0.76	0.92	0.74	0.99	0.74	0.92	0.75	0.95	0.76	0.99	0.21	0.19
**Teratogens**												
9-cis-retinoic acid	1.00	1.00	1.00	1.00	1.00	1.00	1.00	1.00	1.00	1.00	1.00	1.00
Acitretin	1.00	0.95	1.00	1.00	1.00	0.95	1.00	0.98	1.00	1.00	1.00	0.99
Actinomycin D	1.00	1.00	1.00	1.00	1.00	1.00	1.00	1.00	1.00	1.00	1.00	1.00
Atorvastatin	1.00	1.00	0.00	0.00	0.00	0.00	0.50	0.50	1.00	1.00	1.00	1.00
Carbamazepine	0.29	1.00[c]	0.91	1.00	0.29	1.00[c]	0.60	1.00[c]	0.91	1.00[c]	0.17	0.94
Doxorubicin	1.00	1.00	1.00	1.00	1.00	1.00	1.00	1.00	1.00	1.00	1.00	1.00
Entinostat	0.56	1.00	1.00	1.00	0.56	1.00	0.78	1.00	1.00	1.00	0.17	1.00
Favipiravir	0.45	0.93	0.92	0.98	0.45	0.93	0.68	0.96	0.92	0.98	0.31	0.96
Isotretinoin	0.98	1.00	1.00	1.00	0.98	1.00	0.99	1.00	1.00	1.00	1.00	1.00
Leflunomide	0.91	--[c]	1.00	1.00	0.91	--[c]	0.95	--[c]	1.00	--[c]	0.71	--[c]
Lithium chloride	0.44	1.00	0.79	0.94	0.44	0.94	0.62	0.97	0.79	1.00	0.24	1.00
Methotrexate	0.76	0.41	0.99	0.99	0.76	0.41	0.87	0.70	0.99	0.99	0.88	0.89
Methylmercury	0.26	0.61	0.89	0.64	0.26	0.61	0.57	0.63	0.89	0.64	0.12	0.22
Panobinostat	1.00	1.00	1.00	1.00	1.00	1.00	1.00	1.00	1.00	1.00	1.00	1.00
Paroxetine	0.05	1.00	0.27	0.16	0.05	0.16	0.16	0.58	0.27	1.00	0.23	1.00
Phenytoin	0.48	--[c]	0.25	0.25	0.25	--[c]	0.36	--[c]	0.48	--[c]	0.31	0.93
Retinol[d]	--[d]	1.00[d]	--[d]	0.38[d]	--[d]	0.38[d]	--[d]	0.69[d]	--[d]	1.00[d]	--[d]	1.00
Teriflunomide	0.97	--[c]	0.96	0.96	0.96	--[c]	0.97	--[c]	0.97	--[c]	0.91	--[c]
Thalidomide	0.39	0.26	0.37	0.34	0.37	0.26	0.38	0.30	0.39	0.34	0.33	0.49
Trichostatin A	0.85	1.00	1.00	1.00	0.85	1.00	0.93	1.00	1.00	1.00	0.30	1.00
Valproic acid	1.00	1.00[c]	1.00	1.00	1.00	1.00[c]	1.00	1.00[c]	1.00	1.00[c]	1.00	1.00
Vinblastine	1.00	1.00	1.00	1.00	0.22	0.22	0.45	0.45	0.69	0.69	1.00	1.00
Vismodegib	0.22	--[c]	0.69	--[c]	1.00	--[c]	1.00	--[c]	1.00	--[c]	0.13	--[c]
Vorinostat	0.98	1.00	1.00	1.00	0.98	1.00	0.99	1.00	1.00	1.00	1.00	1.00

[a]Maximal plasma or blood concentrations after administration of therapeutic compound dose.

[b]Probability of a compound to be a teratogen in the top-1,000-procedure.

[c]Carbamazepine and valproic acid were tested at 10-fold and 1.67-fold C_{max}, respectively, instead of 20-fold C_{max}; leflunomide, phenytoin, teriflunomide and vismodegib were only tested at 1-fold C_{max} due to limited solubility.

[d]Retinol was considered as a non-teratogen at 1-fold Cmax and as a teratogen at 20-fold C_{max}. Rationale is given in the methods.

[e]RT-qPCR measurements were not available for levothyroxine at 20-fold C_{max}; instead, the RT-qPCR results of levothyroxine at 1-fold C_{max} were used to classify levothyroxine at 20-fold C_{max}.

Table S3 Performance metrics of the test combinations “min” and “max”

Test	Data	Procedure	1-fold C_{max} AUC	1-fold C_{max} Accuracy	1-fold C_{max} Sensitivity	1-fold C_{max} Specificity	20-fold C_{max} [a] AUC	20-fold C_{max} [a] Accuracy	20-fold C_{max} [a] Sensitivity	20-fold C_{max} [a] Specificity
Combination „min“	Cytotoxicity		0.61	0.56	0.26	1	0.63	0.67	0.46	1
	Gene expression	SPS	0.79	0.64	0.39	1	0.86	0.59	0.38	0.93
		Top-1,000	0.88	0.74	0.7	0.81	0.92	0.77	0.71	0.87
	Cytotoxicity and gene expression	SPS	0.84	0.79	0.65	1	0.9	0.87	0.83	0.93
		Top-1,000	0.91	0.87	0.91	0.81	0.94	0.92	0.96	0.87
Combination „max“	Cytotoxicity		0.63	0.56	0.26	1	0.73	0.67	0.46	1
	Gene expression	SPS	0.89	0.77	0.61	1	0.84	0.64	0.42	1
		Top-1,000	0.96	0.74	0.65	0.88	0.94	0.64	0.5	0.87
	Cytotoxicity and gene expression	SPS	0.92	0.92	0.87	1	0.91	0.92	0.88	1
		Top-1,000	0.97	0.9	0.91	0.88	0.97	0.92	0.96	0.87

- Cytotoxicity: Only cytotoxicity data were considered for the calculation of the metrics, i.e., cytotoxic conditions were considered as positive and non-cytotoxic conditions as negative test results.
- Gene expression: Only gene expression data were considered for the calculation of the metrics.
- Cytotoxicity and gene expression: All data for cytotoxicity as well as for gene expression were considered for the calculation of the metrics.
- AUC (Area-under-curve): For each possible cut-off used as threshold, predictions were made for each of the conditions, based on which sensitivity and specificity were calculated. The ROC-curve (receiver operator characteristic) was obtained by plotting all pairs of (1-specificity) and sensitivity against each other. The AUC was determined as the area under this ROC-curve.
- Accuracy: Ratio of correct predictions ((true negatives and positives) / (true and false negatives and positives)) (Supp. Table S 1).
- Sensitivity: Ratio of detected teratogens (true positives/(false negatives + true positives)) (Supp. Table S 1).
- Specificity: Ratio of detected non-teratogens (true negatives / (true negatives + false positives)) (Supp. Table S 1).

[a]Including 10-fold C_{max} carbamazepine, 1.67-fold C_{max} VPA and 1-fold C_{max} samples of leflunomide, phenytoin, teriflunomide and vismodegib.

Table S4 Cytotoxicity and number of significantly deregulated probe sets in the test combinations

	Cytotoxicity[b]						Number of deregulated probe sets[c]					
**Test**	**Min**		**Mean**		**Max**		**Min**		**Mean**		**Max**	
**Compounds** / C_{max}[a]	**1-fold**	**20-fold**	**1-fold**	**20-fold**	**1-fold**	**20-fold**	**1-fold**	**20-fold**	**1-fold**	**20-fold**	**1-fold**	**20-fold**
**Non-teratogens**												
Ampicillin	No	No	No	No	No	No	0	0	68	14	136	28
Ascorbic acid	No	No	No	No	No	No	0	0	52.5	198	105	396
Buspirone	No	No	No	No	No	No	0	0	22	25.5	44	51
Chlorpheniramine	No	No	No	No	No	No	0	0	24.5	20.5	49	41
Dextromethorphan	No	No	No	No	No	No	0	0	59.5	16	119	32
Diphenhydramine	No	No	No	No	No	No	0	0	0	18	0	36
Doxylamine	No	No	No	No	No	No	0	0	37.5	71	75	142
Famotidine	No	No	No	No	No	No	0	0	13	11.5	26	23
Folic acid	No	No	No	No	No	No	0	0	71	65.5	142	131
Levothyroxine	No	No	No	No	No	No	38	0	123	6.5	208	13
Liothyronine	No	No	No	No	No	No	0	36	88.5	60	177	84
Magnesium chloride	No	No	No	No	No	No	0	16	113.5	405	227	794
Methicillin	No	No	No	No	No	No	0	2	25	30	50	58
Ranitidine	No	No	No	No	No	No	0	0	58	56.5	116	113
Retinol[e]	No[e]	--[e]	No[e]	--[e]	No[e]	--[e]	0[e]	--[e]	0[e]	--[e]	0[e]	--[e]
Sucralose	No	No	No	No	No	No	0	33	95.5	83	191	166
**Teratogens**												
9-cis-retinoic acid	No	No	No	No	No	No	731	731	2236	2490.5	3741	4313
Acitretin	No	No	No	No	No	No	708	708	2057.5	2381	3407	4104
Actinomycin D	Yes	Yes	Yes	Yes	Yes	Yes	4257	4257	4287.5	4287.5	4318	4318
Atorvastatin	No	No	Yes	Yes	Yes	Yes	128	128	2223	2341	4318	4318
Carbamazepine	No	No[d]	No	No[d]	No	No[d]	0	0[d]	138	1241.5[d]	276	1501[d]
Doxorubicin	Yes	Yes	Yes	Yes	Yes	Yes	4257	4257	4287.5	4287.5	4318	4318
Entinostat	No	No	No	Yes	No	Yes	78	78	406.5	4285	735	4318
Favipiravir	No	No	No	No	No	No	0	0	80.5	1966	161	2841
Isotretinoin	No	No	No	Yes	No	Yes	1714.5	1714.5	1723.25	3004	1732	4318
Leflunomide	No	--[d]	No	--[d]	No	--[d]	1029	--[d]	2177.5	--[d]	3326	--[d]
Lithium chloride	No	No	No	Yes	No	Yes	0	0	229.5	2941.5	459	4318
Methotrexate	No	No	No	No	No	No	644	644	698	1069.5	752	1309
Methylmercury	No	No	No	No	No	No	0	0	188.5	62	377	124
Panobinostat	Yes	Yes	Yes	Yes	Yes	Yes	4257	4257	4287.5	4287.5	4318	4318
Paroxetine	No	No	No	Yes	No	Yes	0	0	219	2469	438	4318
Phenytoin	No	--[d]	No	--[d]	No	--[d]	0	--[d]	1	--[d]	2	--[d]
Retinol[d]	--[e]	No[e]	--[e]	No[e]	--[e]	No[e]	--[e]	0*	--[e]	1000.5[e]	--[e]	1968[e]
Teriflunomide	No	--[d]	No	--[d]	No	--[d]	1501	--[d]	2362	--[d]	3223	--[d]
Thalidomide	No	No	No	No	No	No	62	62	301.75	503.75	541.5	1007.5
Trichostatin A	No	Yes	No	Yes	No	Yes	4	4	294	4287.5	584	4318
Valproic acid	No	Yes[d]	No	Yes[d]	No	Yes[d]	994	994[d]	1141.5	2060.5[d]	1289	2558[d]
Vinblastine	Yes	Yes	Yes	Yes	Yes	Yes	4257	4257	4287.5	4287.5	4318	4318
Vismodegib	No	--[d]	No	--[d]	No	--[d]	0	--[d]	16	--[d]	32	--[d]
Vorinostat	Yes	Yes	Yes	Yes	Yes	Yes	4257	4257	4287.5	4287.5	4318	4318

[a]Maximal plasma or blood concentrations after administration of therapeutic compound dose.
[b]Yes, if the compound was highly cytotoxic; No, if the compound showed no cytotoxicity.
[c]Gene array-probe sets that were deregulated with an FDR-adjusted p-value<0.05 and an absolute fold-change>2 compared to untreated control cells.
[d]Carbamazepine and VPA were tested at 10-fold and 1.67-fold C_{max}, respectively, instead of 20-fold C_{max}; leflunomide, phenytoin, teriflunomide and vismodegib were only tested at 1-fold C_{max} due to limited solubility.
[e]Retinol was considered as a non-teratogen at 1-fold C_{max} and as a teratogen at 20-fold C_{max}. Rationale is given in the methods.

Fig. S1-S5 Biological interpretation of genes differentially expressed after exposure of hiPSC to teratogens in the UKN1 test. The following probe sets were investigated:

- Fig. S1: All deregulated probe sets at 1-fold C_{max}
- Fig. S2: Only upregulated probe sets at 1-fold C_{max}
- Fig. S3: Only downregulated probe sets at 1-fold C_{max}
- Fig. S4: Only upregulated probe sets at 20-fold C_{max}
- Fig. S5: Only downregulated probe sets at 20-fold C_{max}

(A) Number of significant probe sets (log2 fold change >1; adjusted p-value <0.05) induced by non-teratogens and teratogens at the given concentration. 20-fold C_{max} samples included also 10-fold C_{max} carbamazepine and 1.67-fold C_{max} VPA. **(B)** Top-10 genes deregulated by teratogens. The number in the bar indicates the number of compounds that deregulated the specific gene. The (absolute) mean log fold-change of each gene is given on the x-axis. A comprehensive gene list is given in the Supp. excel-file 1. **(C)** KEGG pathway enrichment analysis of the genes deregulated by teratogens. The ten KEGG pathways with the lowest adj. p-values are given. Full names and complete KEGG-pathway lists are given in the Supp. excel-file 2. "Count": Number of significant genes from A linked to the KEGG pathway. "Gene Ratio": Percentage of significant genes associated with the pathway compared to the number of all significant genes associated with any pathway. **(D)** The ten GO groups with the lowest adj. p-values from all significantly (adj. p-value <0.05) overrepresented GO groups of genes deregulated by teratogens. The names of the GO groups were shortened. Full names and complete GO group lists can be found in the Supp. excel-file 3. "Count": Number of significant genes from A linked to the GO group. "Hits": Percentage of significant genes compared to all genes assigned to the GO group.

Fig. S1:

All probe sets at 1-fold C_{max}

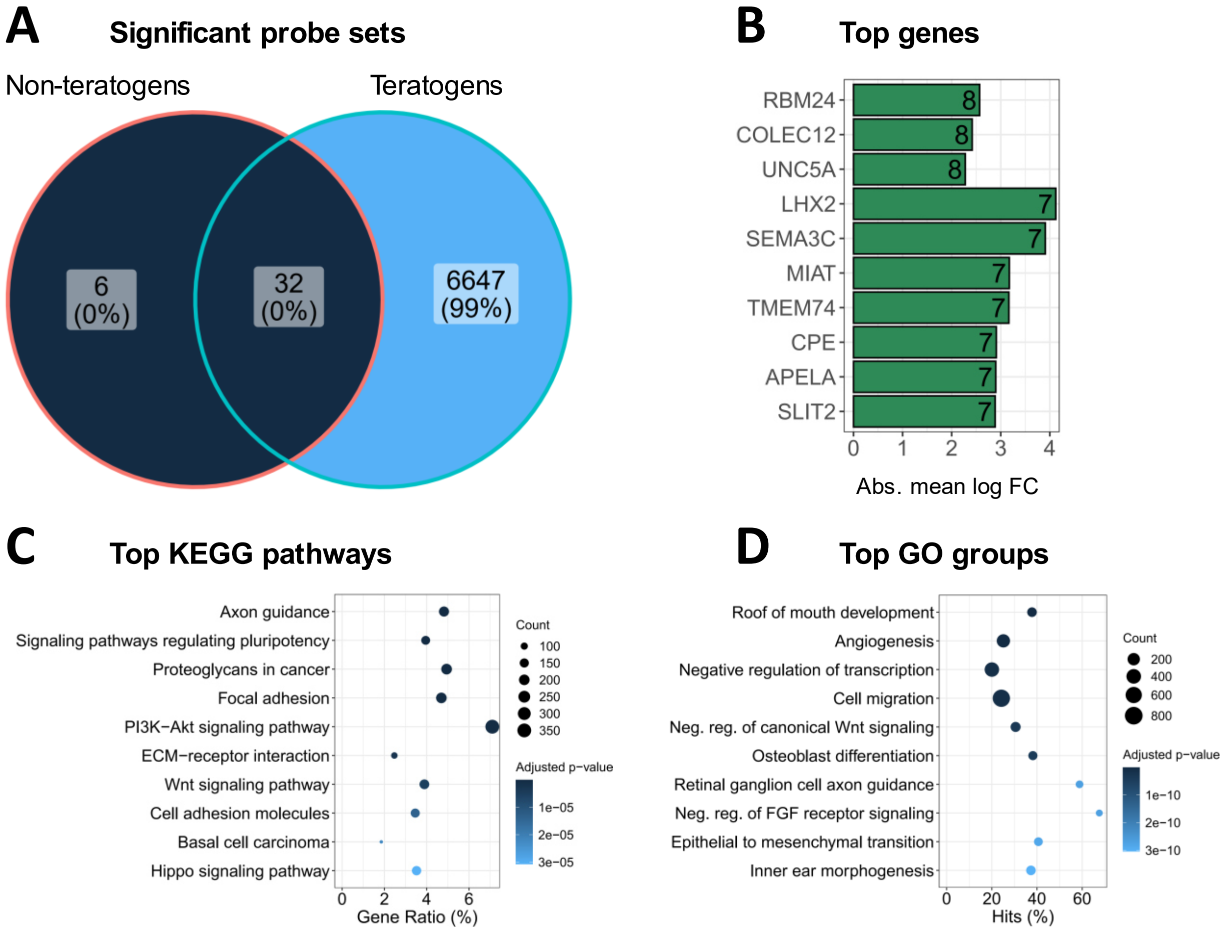

Fig. S2:
Upregulated probe sets at 1-fold C_{max}

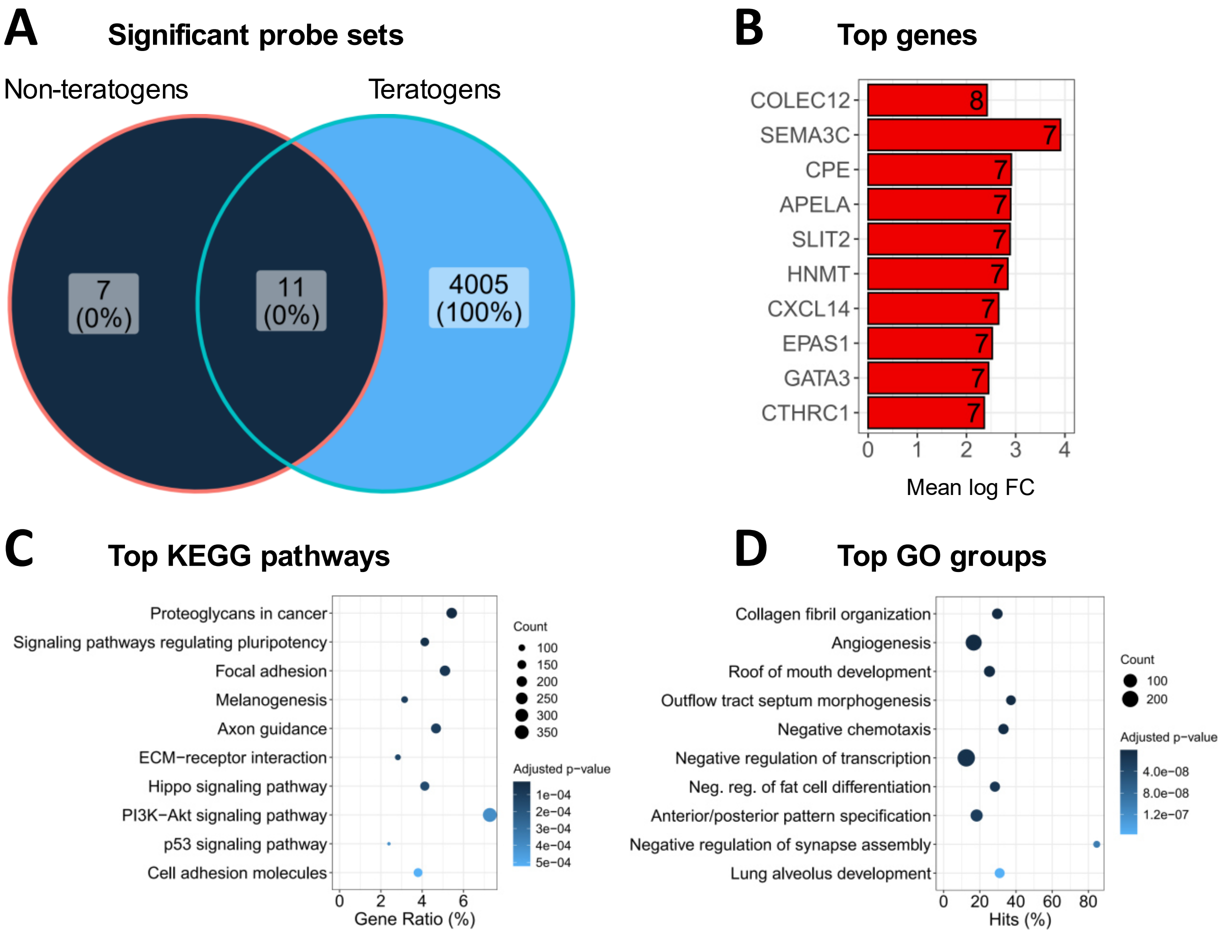

Fig. S3:

Downregulated probe sets at 1-fold C_{max}

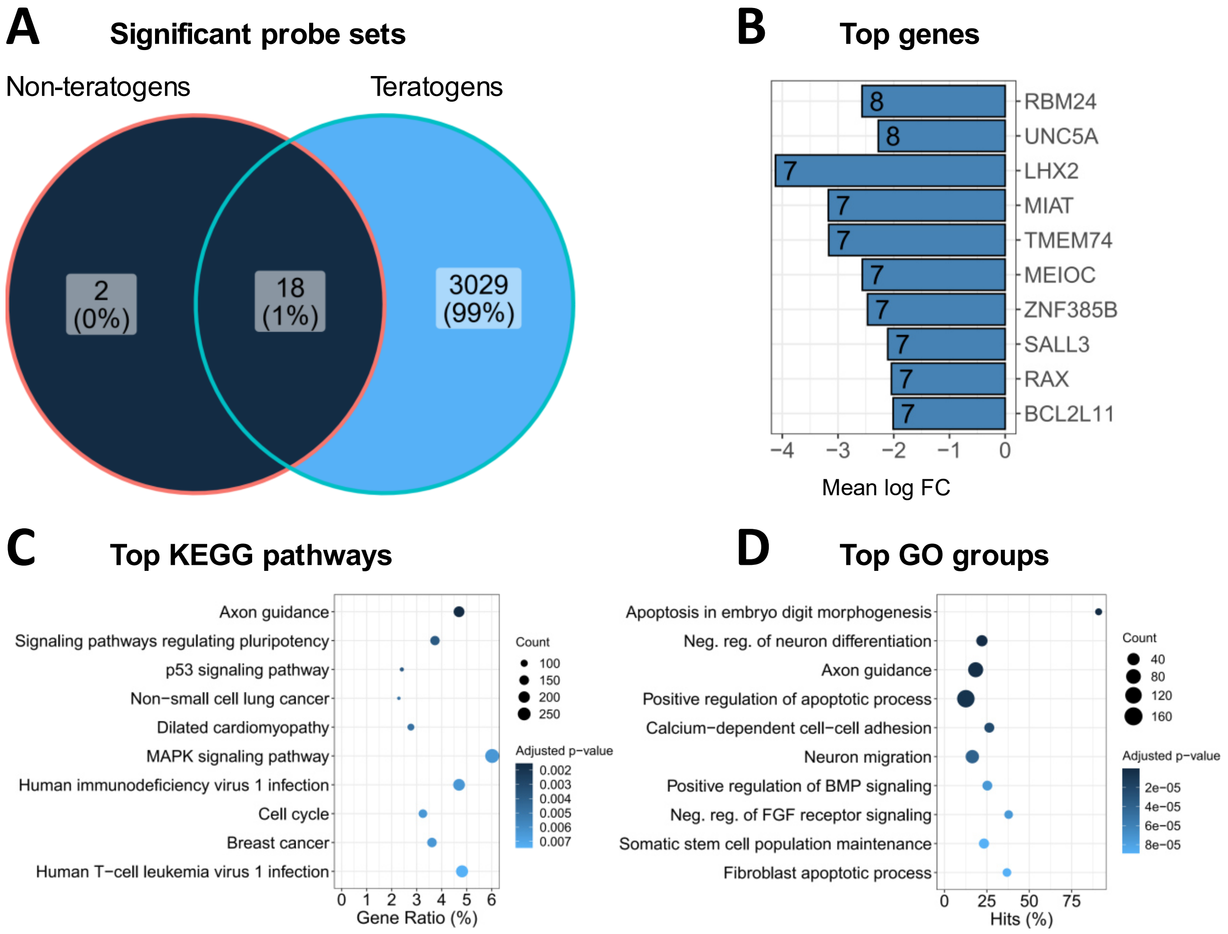

Fig. S4:
Upregulated probe sets at 20-fold C_{max}

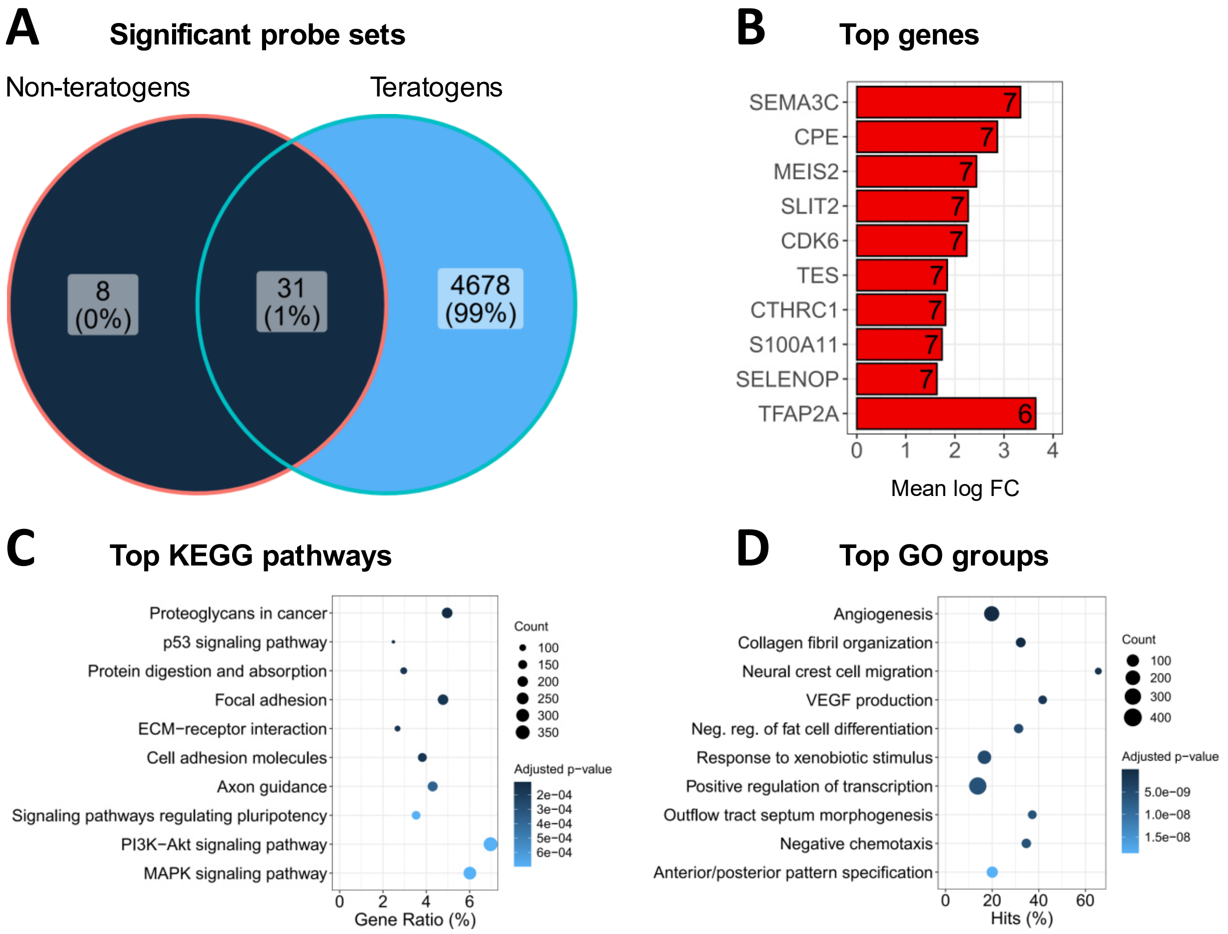

Fig. S5:

Downregulated probe sets at 20-fold C_{max}

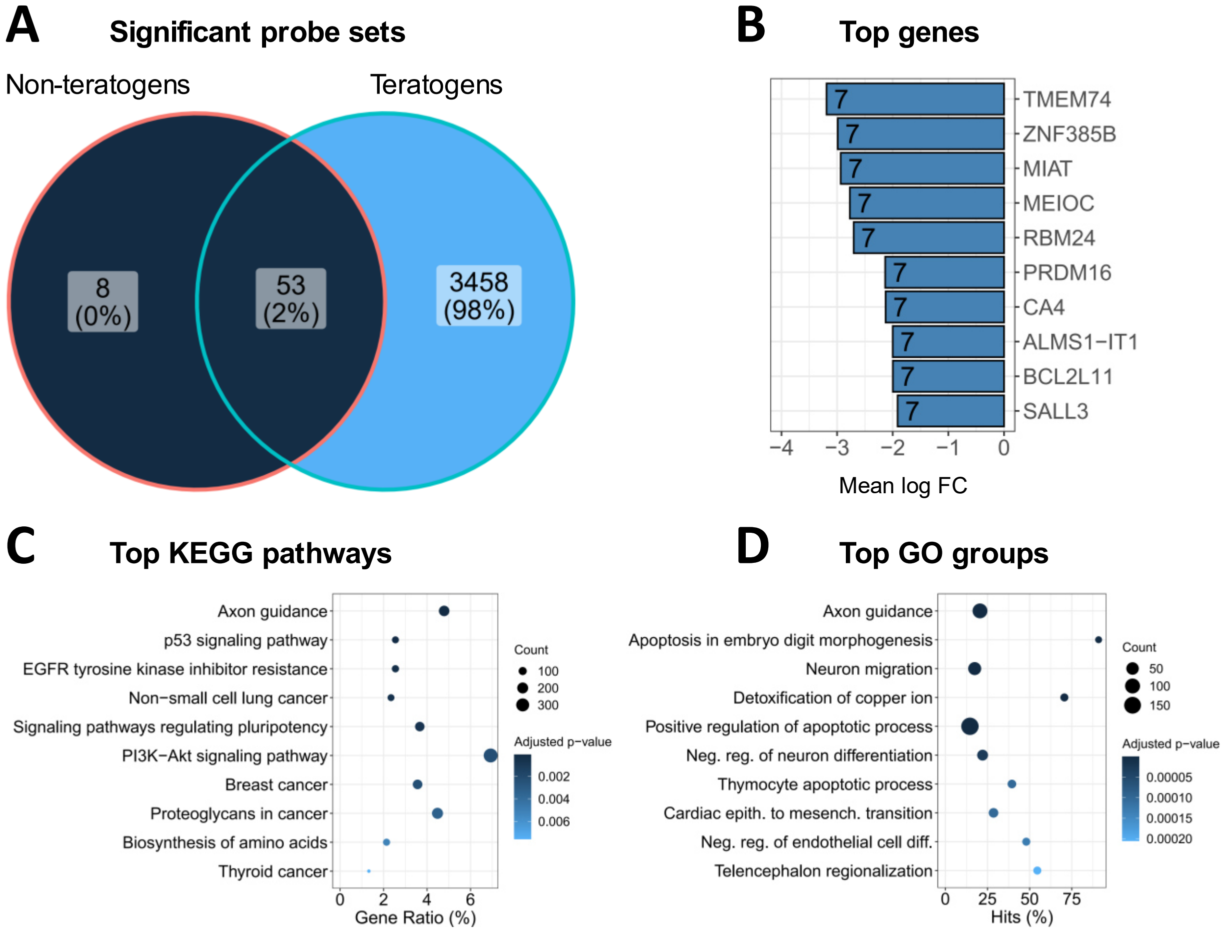

Fig. S6-S10 Biological interpretation and comparison of genes differentially expressed in the UKN1 and UKK2 test after exposure of hiPSC to teratogens. The following probe sets were investigated:

- Fig. S6: All deregulated probe sets at 1-fold C_{max}
- Fig. S7: Only upregulated probe sets at 1-fold C_{max}
- Fig. S8: Only downregulated probe sets at 1-fold C_{max}
- Fig. S9: Only upregulated probe sets at 20-fold C_{max}
- Fig. S10: Only downregulated probe sets at 20-fold C_{max}

(A) Number of significant probe sets (log2 fold change >1; adjusted p-value <0.05) induced by non-teratogens and teratogens at the given concentration. 20-fold C_{max} samples included also 10-fold C_{max} carbamazepine and 1.67-fold C_{max} VPA. **(B)** Number of significantly (adj. p-value <0.05) overrepresented GO groups in the overlap, UKN1 and UKK2 gene set. **(C)** KEGG pathway enrichment analysis of the overlap, UKN1 and UKK2 gene set. The ten KEGG pathways with the lowest adj. p-values are given. Full names and complete KEGG-pathway lists are given in the Supp. excel-file 4. “Count”: Number of significant genes from A linked to the KEGG pathway. “Gene Ratio”: Percentage of significant genes associated with the pathway compared to the number of all significant genes associated with any pathway. **(D)** The ten GO groups with the lowest adj. p-values from all significantly (adj. p-value <0.05) overrepresented GO groups from B that were overrepresented in both tests (‘overlap’) or exclusively in one of the tests ‘UKN1’ and ‘UKK2’. The names of the GO groups were shortened. Full names and complete GO group lists can be found in the Supp. excel-file 5. “Count”: Number of significant genes from A linked to the GO group. “Hits”: Percentage of significant genes compared to all genes assigned to the GO group. **(E)** Top-10 genes deregulated by teratogens within each gene set. The number in the bar indicates the number of compounds that deregulated the specific gene. The absolute mean log fold-change of each gene is given on the x-axis. A comprehensive gene list is given in the Supp. excel-file 6.

Fig. S6:

All probe sets at 1-fold C_{max}

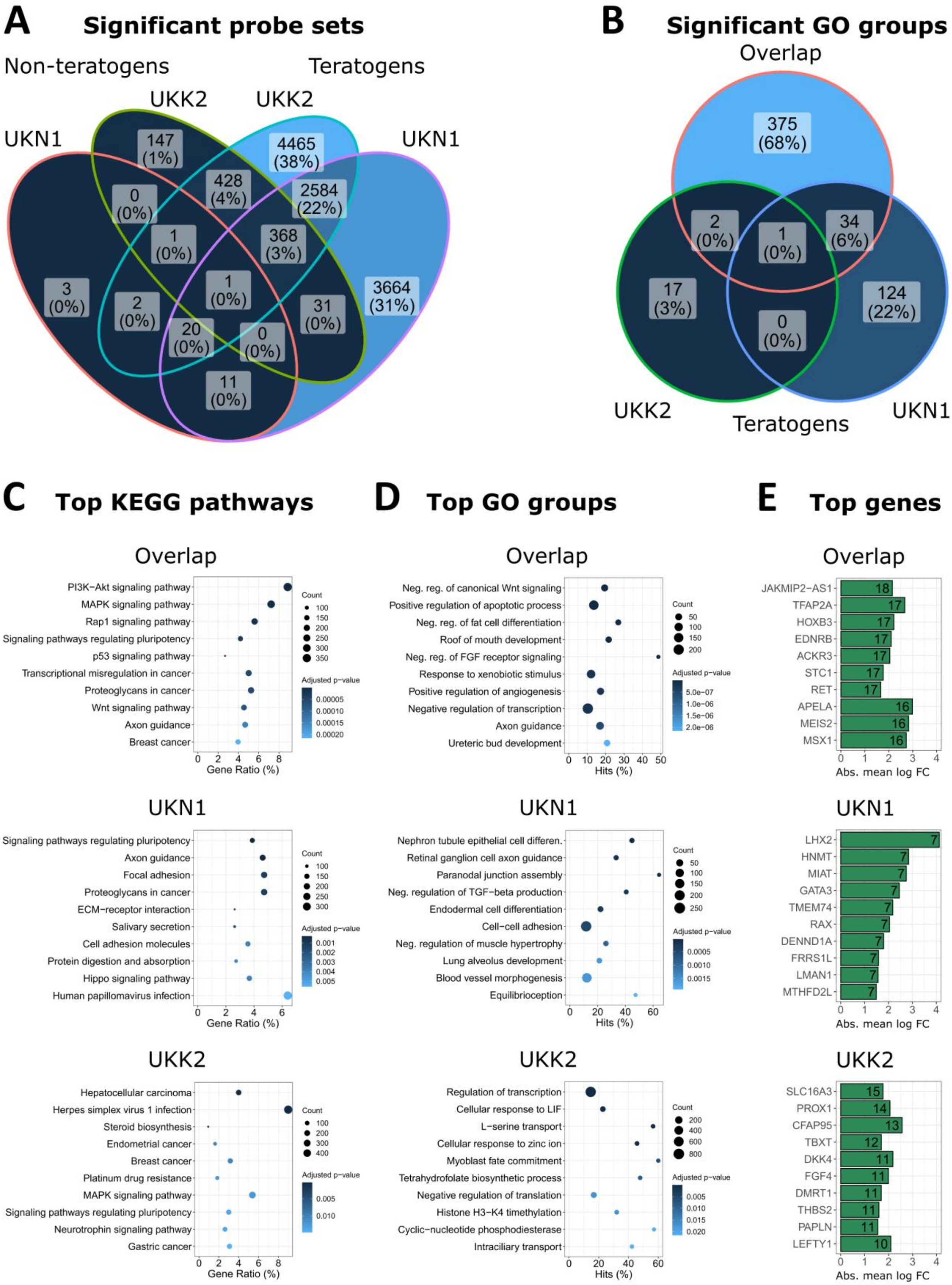

Fig. S7:

Upregulated probe sets at 1-fold C_{max}

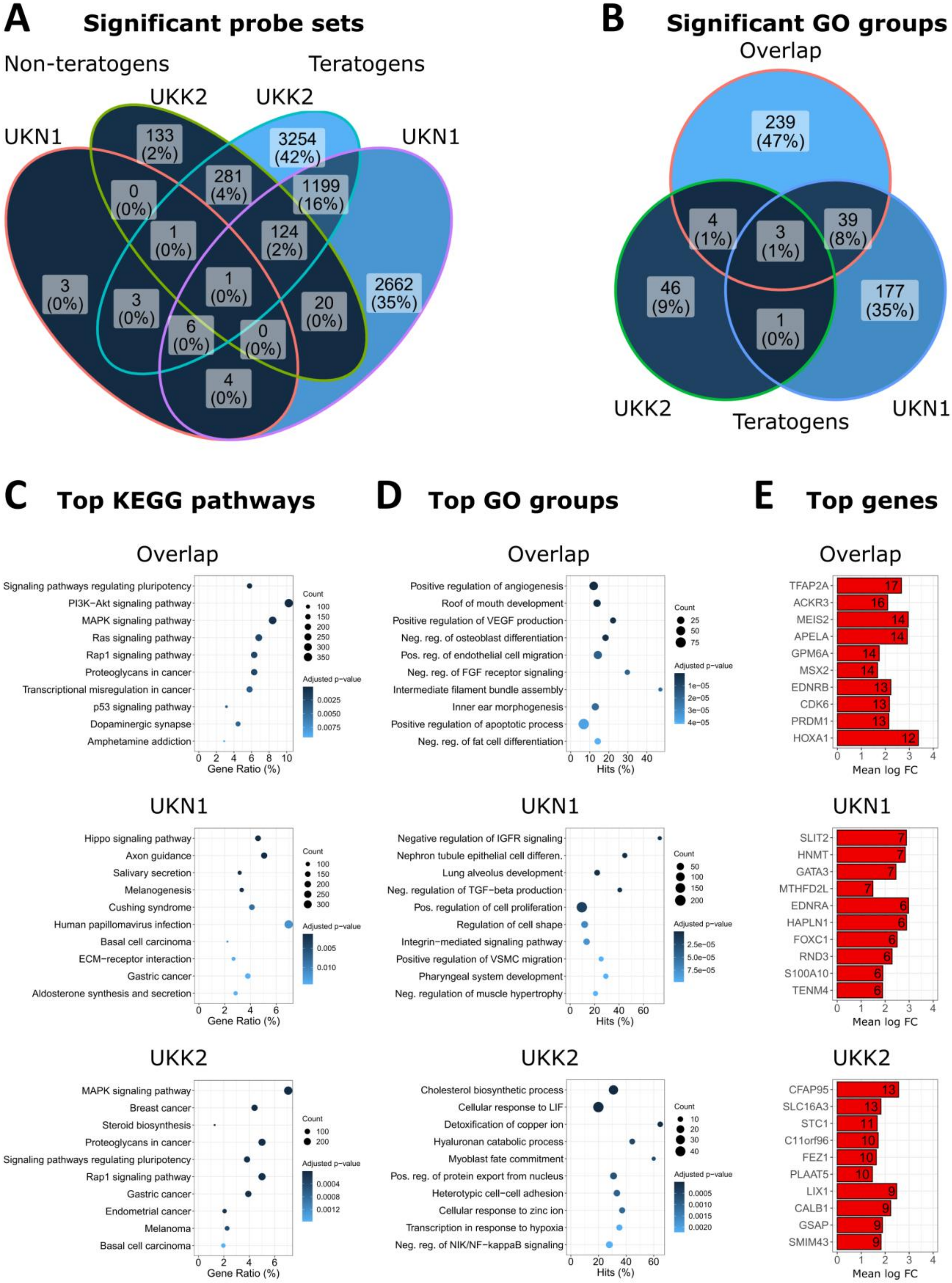

Fig. S8:

Downregulated probe sets at 1-fold C_{max}

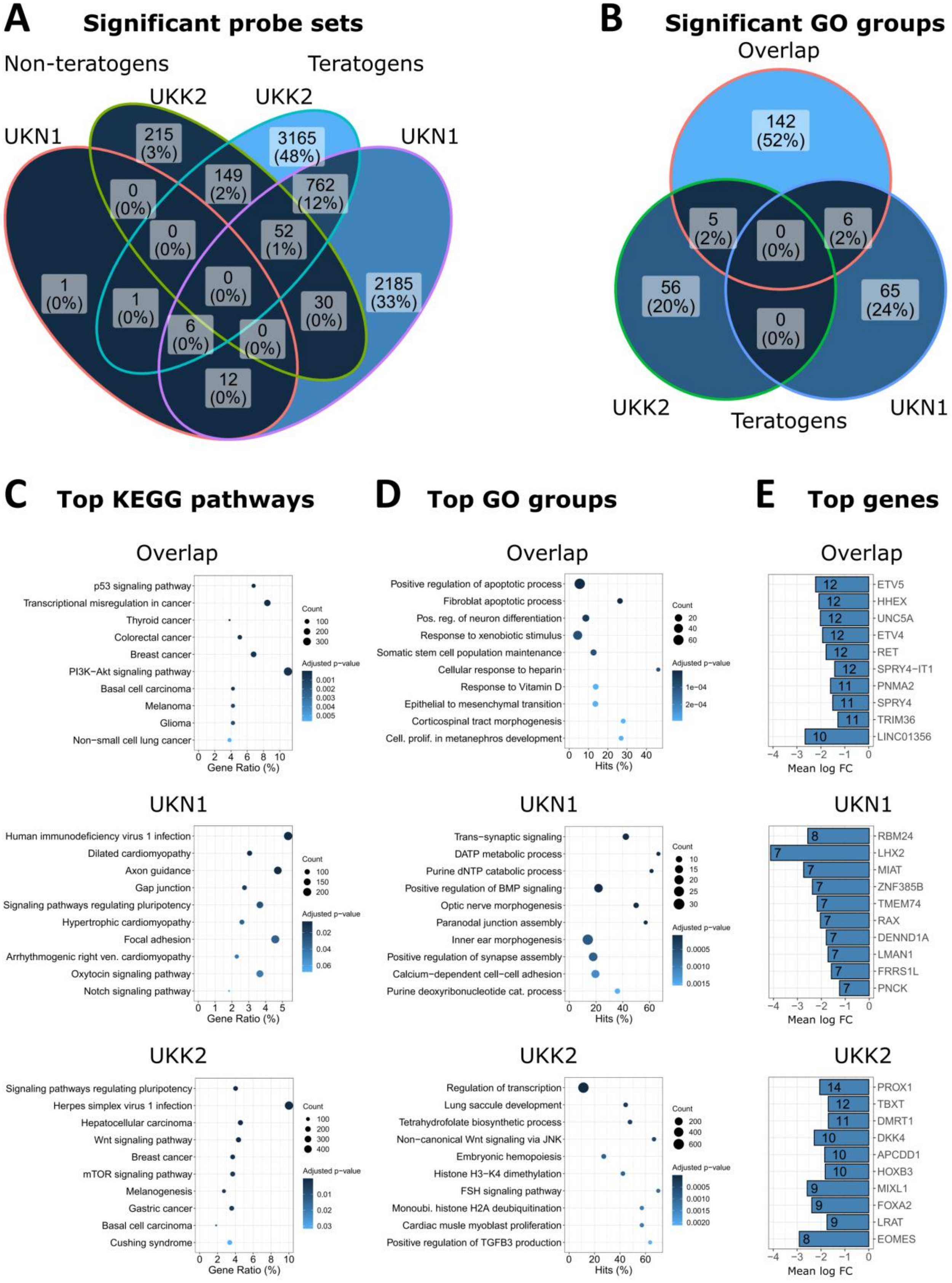

Fig. S9:

Upregulated probe sets at 20-fold C_{max}

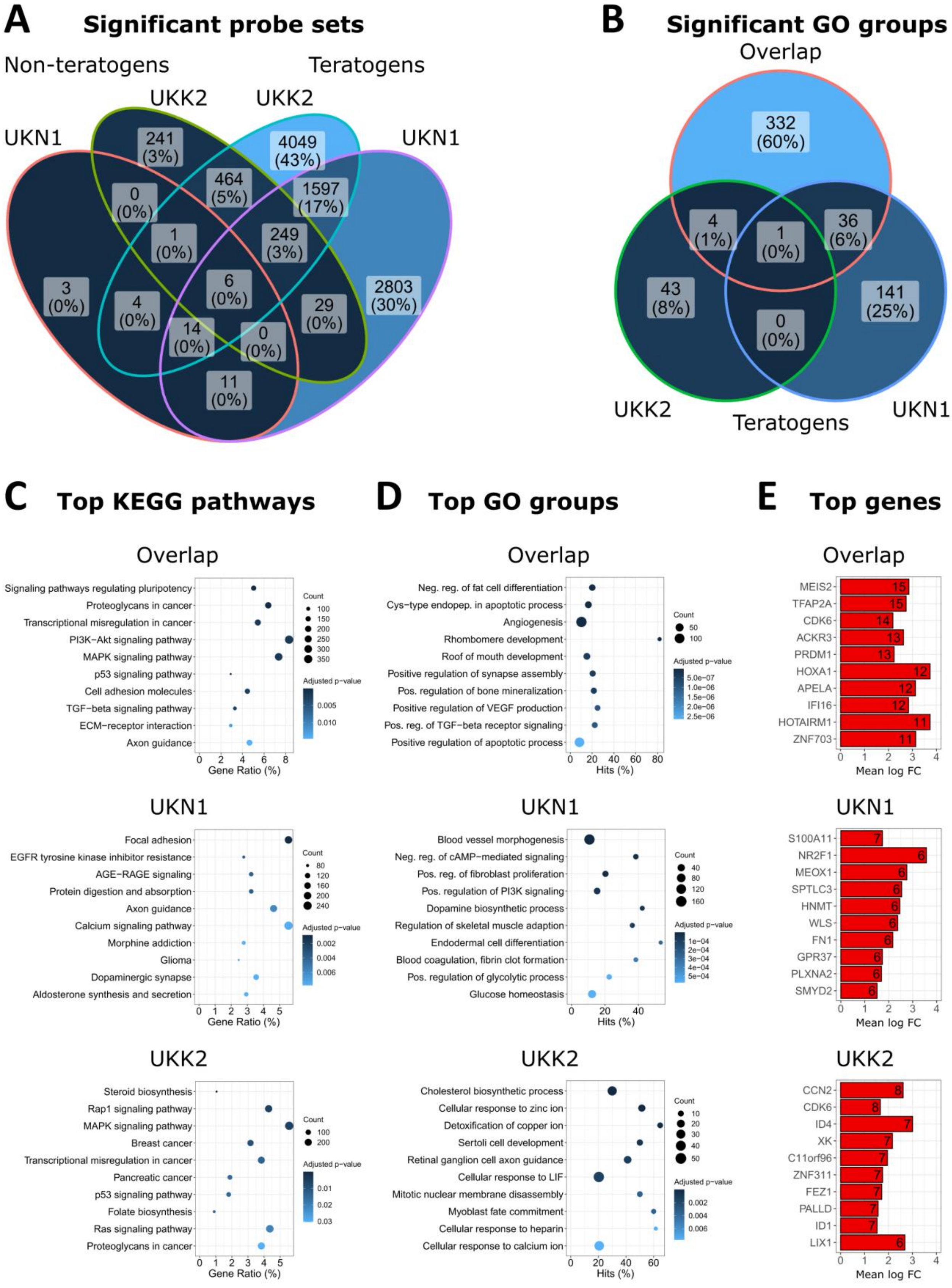

Fig. S10:

Downregulated probe sets at 20-fold C_{max}

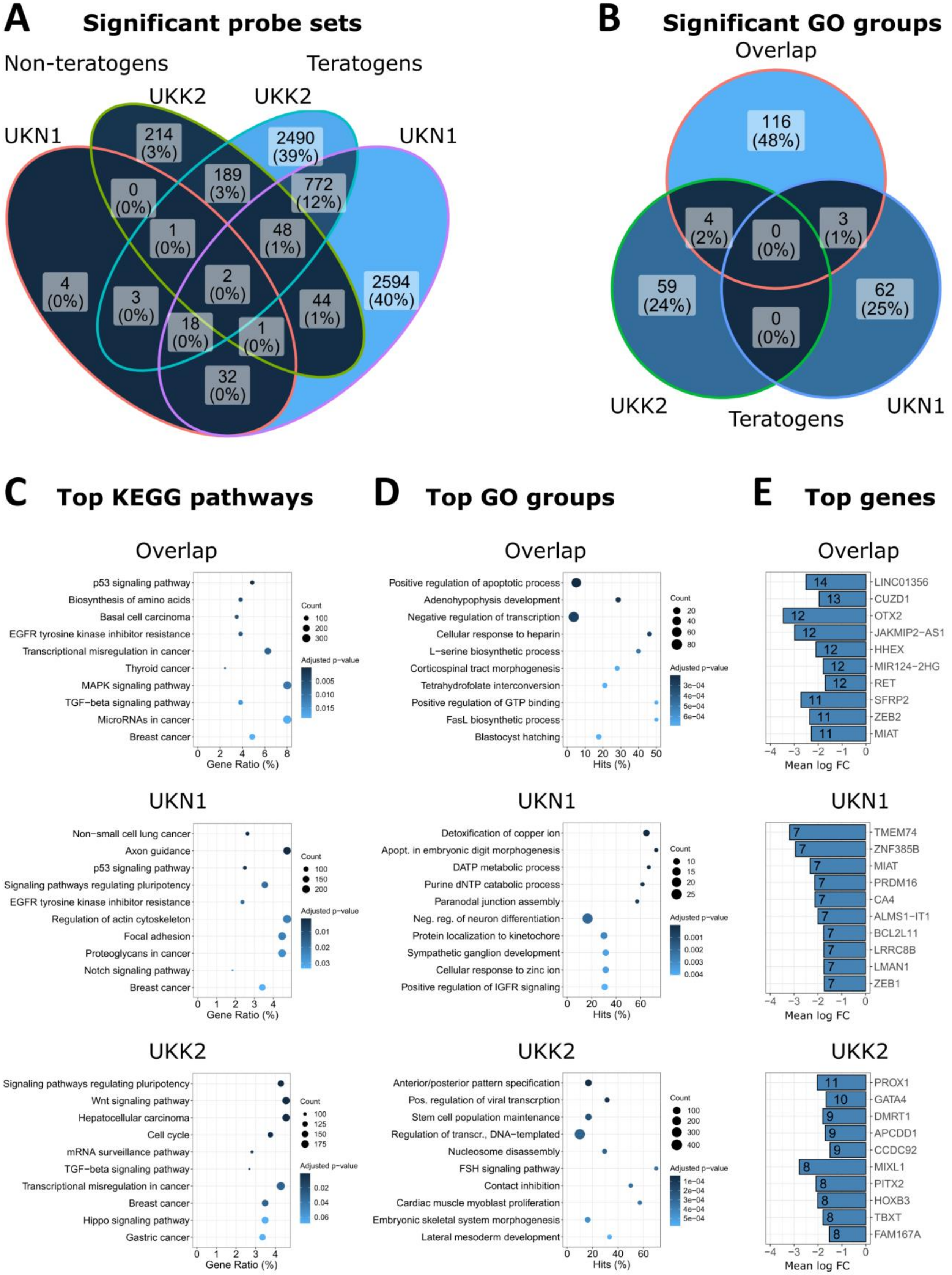

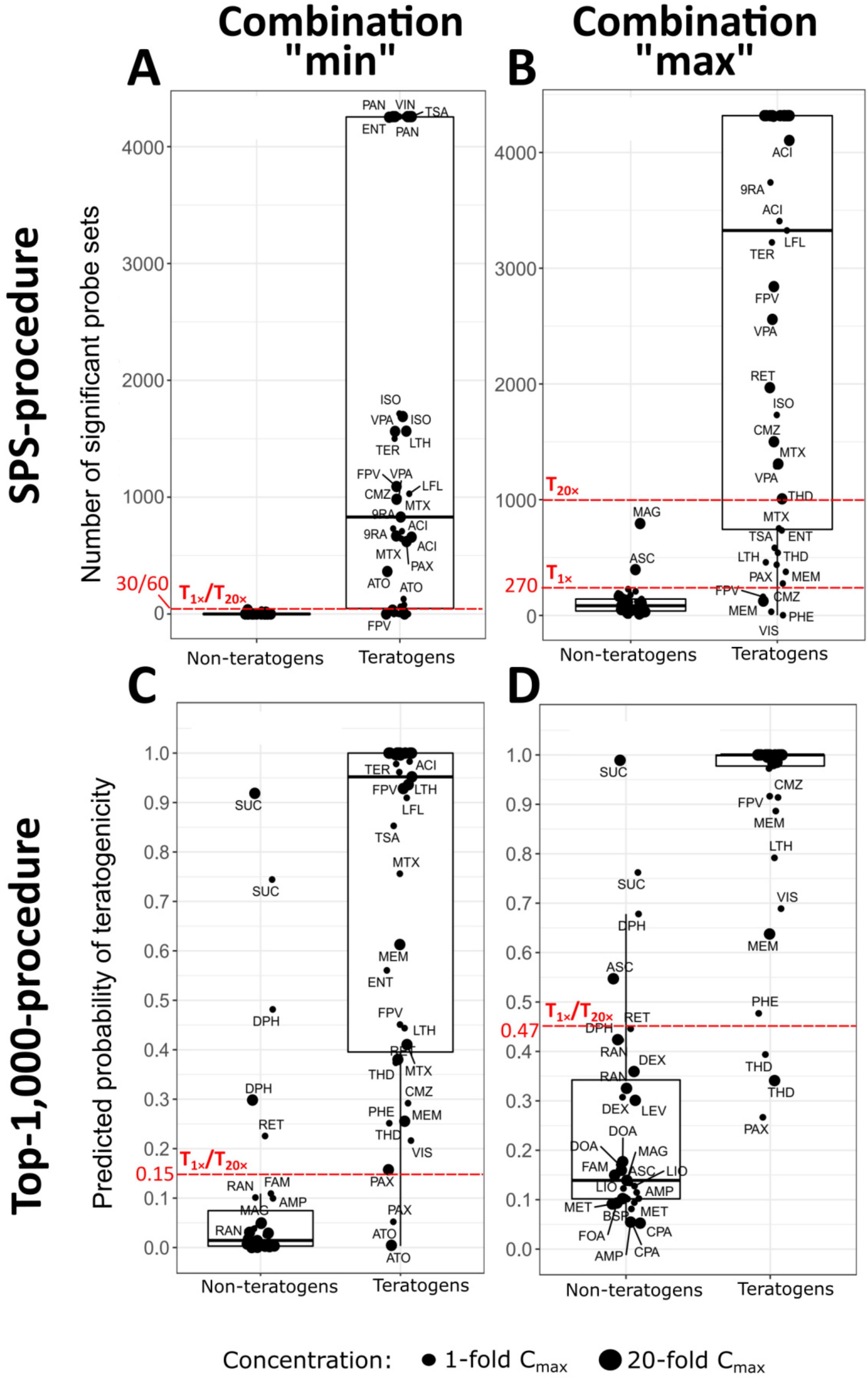
Combination "min"
Combination "max"
A
B
C
D
SPS-procedure
Top-1,000-procedure
Number of significant probe sets
Predicted probability of teratogenicity
30/60
$T_{1\times}/T_{20\times}$
$T_{20\times}$
$T_{1\times}$
270
0.15
0.47
Non-teratogens
Teratogens
Concentration: 1-fold C_{max} 20-fold C_{max}

Fig. S11 Classification of the teratogenic and non-teratogenic compounds by (**A** and **B**) combinations of the SPS-procedures and (**C** and **D**) the top-1,000-procedures of the UKN1 and UKK2 test. The number of significant probe sets (SPS) and the predicted probability for teratogenicity was based on combinations of the UKN1 and UKK2 test results (Fig. 4) and given on the y-axis. The x-axis marks non-teratogens and teratogens (compound abbreviations are explained in Table 1). The thresholds separate negative and positive in vitro test results of 1-fold C_{max} (1×)- and 20-fold-C_{max} (20×)-test conditions for the calculation of accuracy, sensitivity and specificity. Thresholds were set so that at first the accuracy, second the sensitivity and third the specificity was maximized. Thresholds "min" (A): 1-fold-C_{max}: 30 SPS; 20-fold-C_{max}: 60 SPS; "max" (B): 1-fold-C_{max}: 270 SPS; 20-fold-C_{max}: 1000 SPS; "min" (C): 1-fold/20-fold-C_{max}: 0.15; "max" (D): 1-fold/20-fold-C_{max}: 0.47.
Key rules of the combinations:
- "Min": For each compound and concentration, the lowest observed number of SPS / predicted probability in the two test (UKN1 or UKK2) was selected.
- "Max": For each compound and concentration, the highest observed number of SPS / predicted probability in the two test (UKN1 or UKK2) was selected.
- Cytotoxicity (only C and D): In "min", cytotoxic compounds and conditions were predicted with a probability of 1, if they were cytotoxic in both tests (UKN1 **and** UKK2); in "max", cytotoxic compounds and conditions were predicted with a probability of 1, if they were cytotoxic in one of the tests (UKN1 **or** UKK2).

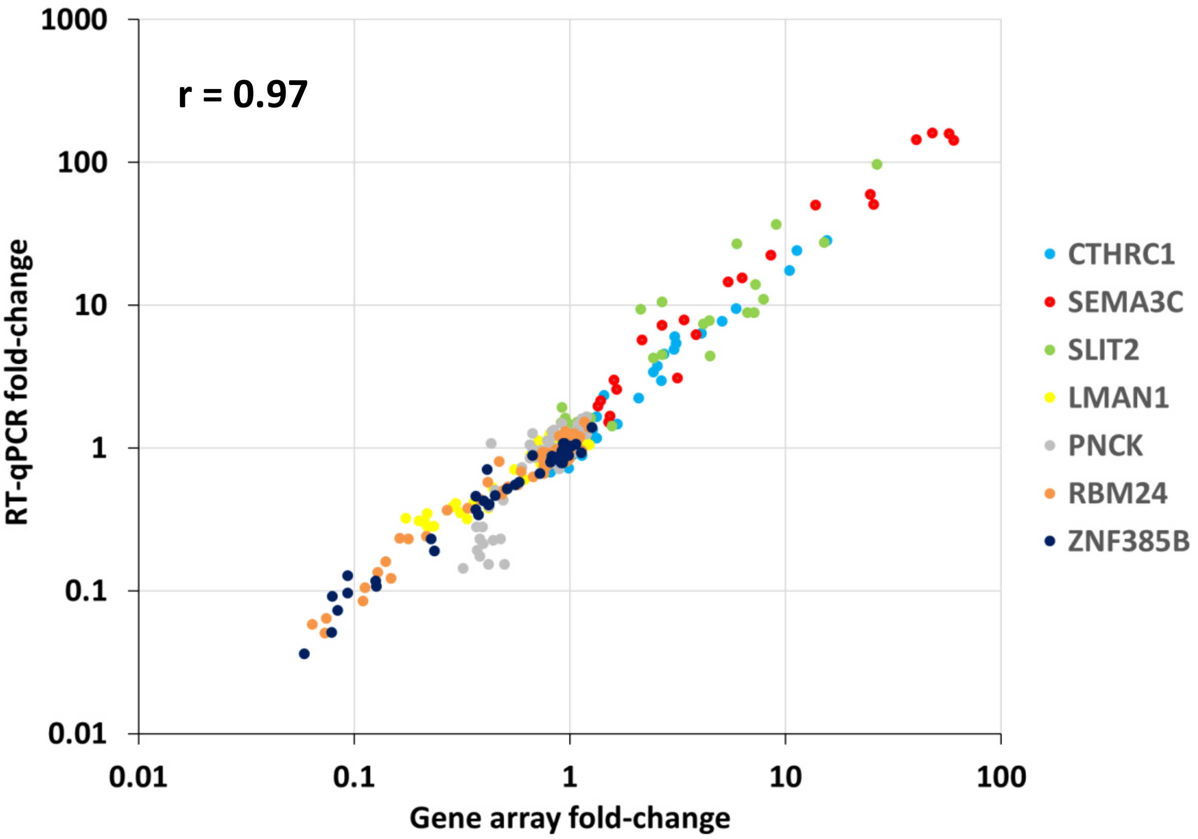

Fig. S12 Correlation plot of substance-induced gene expression changes in UKN1 measured in gene arrays and RT-qPCR. The gene expression fold-change of the seven biomarkers *CTHRC1* (light blue), *SEMA3C* (red), *SLIT2* (green), *LMAN1* (yellow), *PNCK* (grey), *RBM24* (orange) and *ZNF385B* (dark blue) in UKN1 samples that was initially obtained by gene array measurements (x-axis) and reproduced by RT-qPCR analysis (y-axis). The correlation is given with r = 0.97.

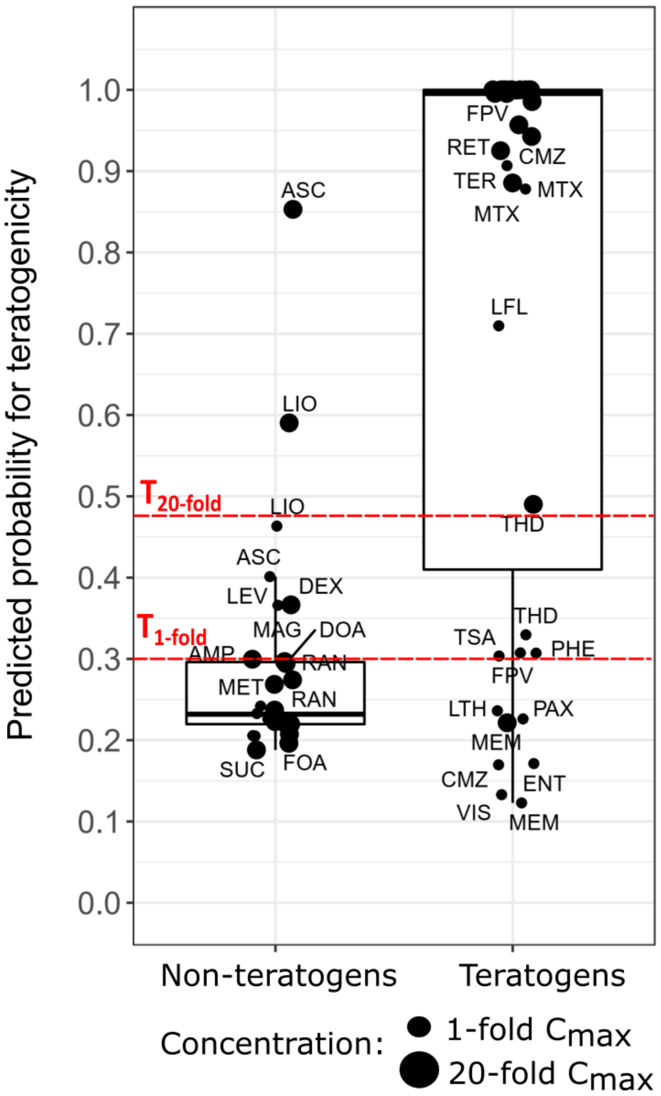

Fig. S13 Classification of the teratogenic and non-teratogenic compounds based on the expression of 7-top-genes of the UKN1 test measured in RT-qPCR. The predicted probability for teratogenicity was calculated by a similar approach as used for the top-1,000-procedure and is given on the y-axis. The x-axis marks non-teratogens and teratogens (compound abbreviations are explained in Table 1). The thresholds separate negative and positive in vitro test results of 1-fold C_{max} (1×)- and 20-fold-C_{max} (20×)-test conditions for the calculation of accuracy, sensitivity and specificity. Thresholds were set so that at first the accuracy, second the sensitivity and third the specificity was maximized. Thresholds: 1-fold-C_{max}: 0.304; 20-fold-C_{max}: 0.490.

Key rules of the top-1,000-like-procedure:

- The calculation of the probability was based on a leave-one-out-cross-validation-algorithm and the 7 top genes measured in RT-qPCR.
- Cytotoxic conditions were considered to be 100% positive (predicted probability of 1.0).

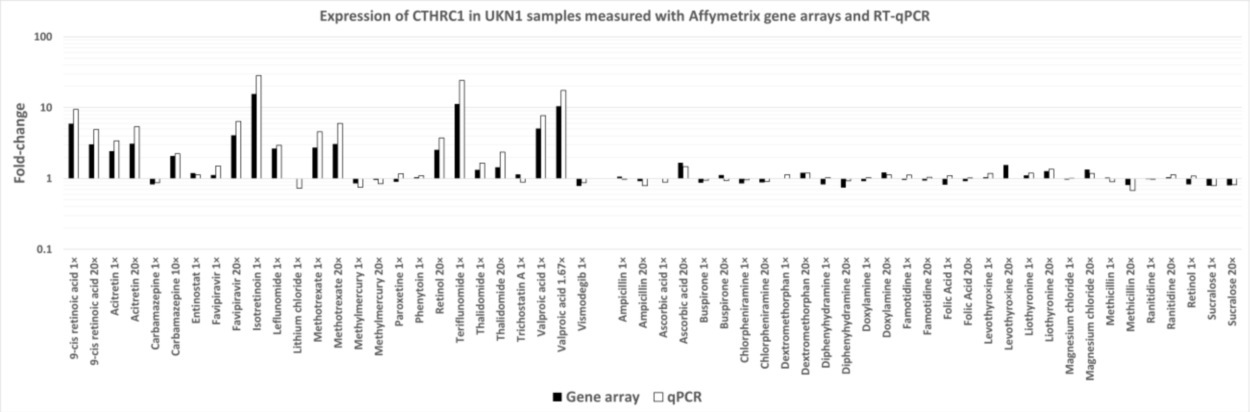

Fig. S14 Expression changes of *CTHRC1* in UKN1 samples relative to controls obtained by gene array (black bars) and RT-qPCR (white bars). Substances and concentrations (i.e., 1-fold, 1.67-fold, 10-fold or 20-fold C_{max}) are given on the x-axis, gene expression fold-changes are given on the y-axis. $\Delta\Delta C_T$-values of the RT-qPCR-analysis are summarized in Suppl. excel-file 7.

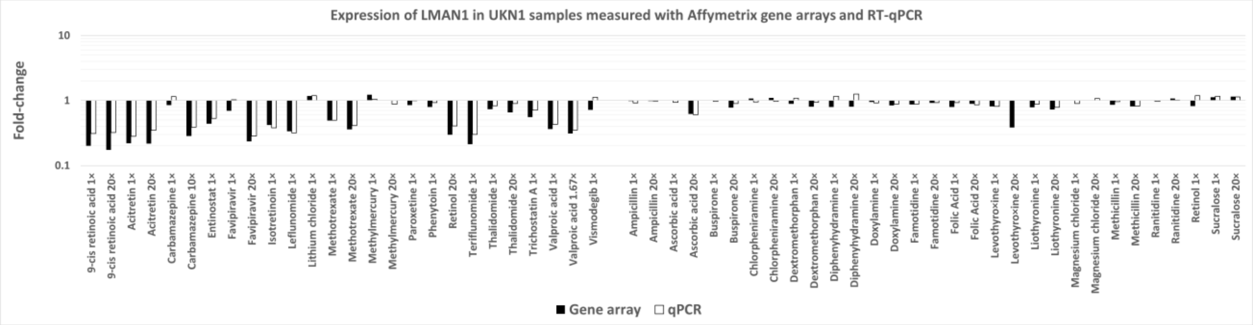

Fig. S15 Expression changes of *LMAN1* in UKN1 samples relative to controls obtained by gene array (black bars) and RT-qPCR (white bars). Substances and concentrations (i.e., 1-fold, 1.67-fold, 10-fold or 20-fold C_{max}) are given on the x-axis, gene expression fold-changes are given on the y-axis. $\Delta\Delta C_T$-values of the RT-qPCR-analysis are summarized in Suppl. excel-file 7.

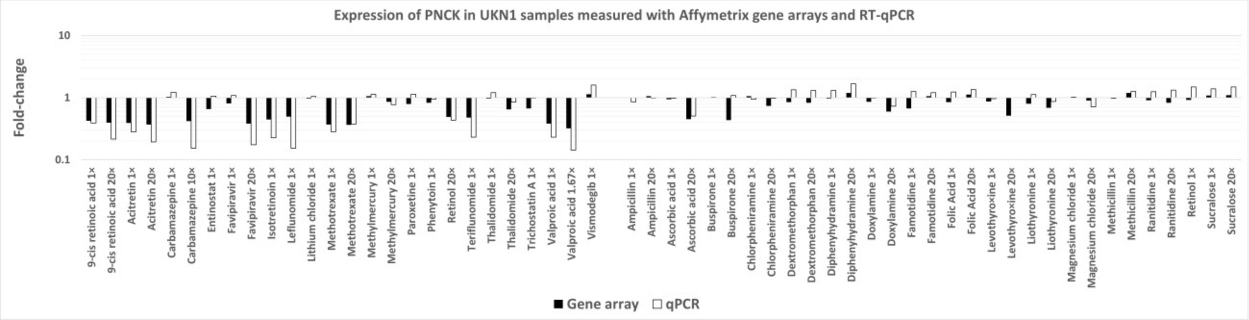

Fig. S16 Expression changes of *PNCK* in UKN1 samples relative to controls obtained by gene array (black bars) and RT-qPCR (white bars). Substances and concentrations (i.e., 1-fold, 1.67-fold, 10-fold or 20-fold C_{max}) are given on the x-axis, gene expression fold-changes are given on the y-axis. $\Delta\Delta C_T$-values of the RT-qPCR-analysis are summarized in Suppl. excel-file 7.

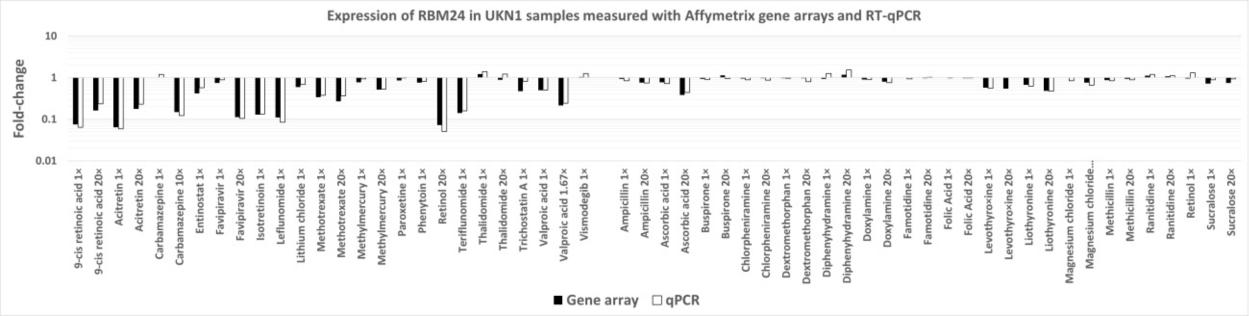

Fig. S17 Expression changes of *RBM24* in UKN1 samples relative to controls obtained by gene array (black bars) and RT-qPCR (white bars). Substances and concentrations (i.e., 1-fold, 1.67-fold, 10-fold or 20-fold C_{max}) are given on the x-axis, gene expression fold-changes are given on the y-axis. $\Delta\Delta C_T$-values of the RT-qPCR-analysis are summarized in Suppl. excel-file 7.

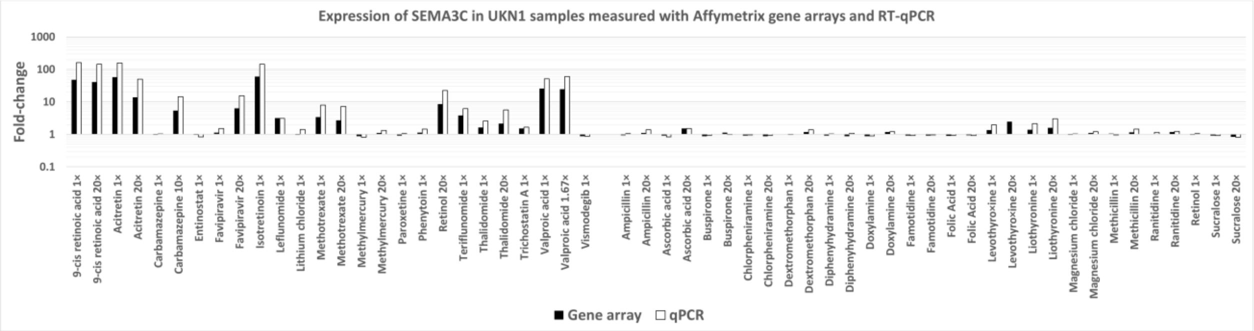

Fig. S18 Expression changes of *SEMA3C* in UKN1 samples relative to controls obtained by gene array (black bars) and RT-qPCR (white bars). Substances and concentrations (i.e., 1-fold, 1.67-fold, 10-fold or 20-fold C_{max}) are given on the x-axis, gene expression fold-changes are given on the y-axis. $\Delta\Delta C_T$-values of the RT-qPCR-analysis are summarized in Suppl. excel-file 7.

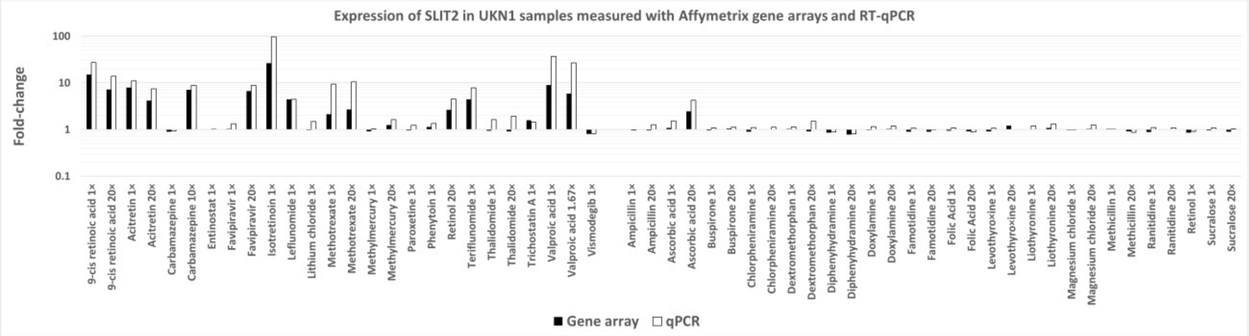

Fig. S19 Expression changes of *SLIT2* in UKN1 samples relative to controls obtained by gene array (black bars) and RT-qPCR (white bars). Substances and concentrations (i.e., 1-fold, 1.67-fold, 10-fold or 20-fold C_{max}) are given on the x-axis, gene expression fold-changes are given on the y-axis. $\Delta\Delta C_T$-values of the RT-qPCR-analysis are summarized in Suppl. excel-file 7.

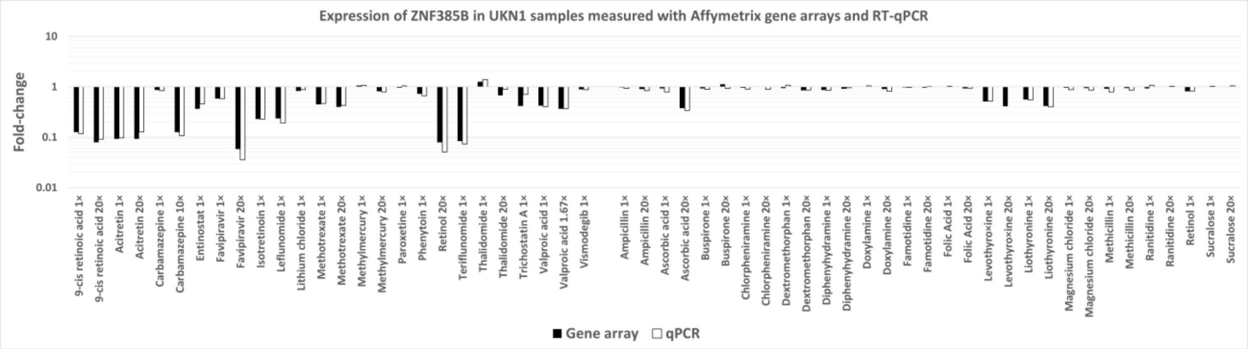

Fig. S20 Expression changes of *ZNF385B* in UKN1 samples relative to controls obtained by gene array (black bars) and RT-qPCR (white bars). Substances and concentrations (i.e., 1-fold, 1.67-fold, 10-fold or 20-fold C_{max}) are given on the x-axis, gene expression fold-changes are given on the y-axis. $\Delta\Delta C_T$-values of the RT-qPCR-analysis are summarized in Suppl. excel-file 7.
