# Supplementary material for: High Accuracy Classification of Developmental Toxicants by In Vitro Tests of Human Neuroepithelial and Cardiomyoblast Differentiation
Source: Cells. 2022 Oct 27;11(21):3404. doi: 10.3390/cells11213404 (PMC9653768; doi:10.3390/cells11213404)
Supplement: Supplementary file 1 [file cells-11-03404-s001.zip › Supporting information 2.pdf]

SUPPORTING INFORMATION 2

High accuracy classification of developmental toxicants by in vitro tests of human neuroepithelial and cardiomyoblast differentiation

Florian Seidel [1,*,‡], Anna Cherianidou [2,‡], Franziska Kappenberg [3,‡], Miriam Marta [1], Nadine Dreser [4], Jonathan Blum [4], Tanja Waldmann [5], Nils Blüthgen [6,7], Johannes Meisig [6,7], Katrin Madjar [3], Margit Henry [2,8], Tamara Rotshteyn [2,8], Andreas Scholtz-Illigens [1], Rosemarie Marchan [1], Karolina Edlund [1], Marcel Leist [4,†], Jörg Rahnenführer [3,†], Agapios Sachinidis [2,8,†] and Jan G. Hengstler [1,*,†]

1 Leibniz Research Centre for Working Environment and Human Factors (IfADo), Technical University of Dortmund, Ardeystrasse 67, 44139 Dortmund, Germany
2 Working Group Sachinidis, Center for Physiology, Faculty of Medicine and University Hospital Cologne, University of Cologne, Robert-Koch-Str. 39, 50931 Cologne, Germany
3 Department of Statistics, TU Dortmund University, Vogelpothsweg 87, 44227 Dortmund, Germany
4 In Vitro Toxicology and Biomedicine, Department of Biology, University of Konstanz, Universitätsstr. 10, 78454 Konstanz, Germany
5 Department of Advanced Cell Systems, trenzyme GmbH, Byk-Gulden-Str. 2, 78467 Konstanz, Germany
6 Institute of Pathology, Charité-Universitätsmedizin Berlin, Chariteplatz 1, 10117 Berlin, Germany
7 IRI Life Sciences, Humboldt Universität zu Berlin, Philippstraße 13, Haus 18, 10115 Berlin, Germany
8 Center for Molecular Medicine Cologne (CMMC), University of Cologne, 50931 Cologne, Germany
* Correspondence: seidelf@ifado.de (F.S.); hengstler@ifado.de (J.G.H.)
† These authors contributed equally to this work.
‡ These authors contributed equally to this work.

Table of Contents

Volcano plots 3

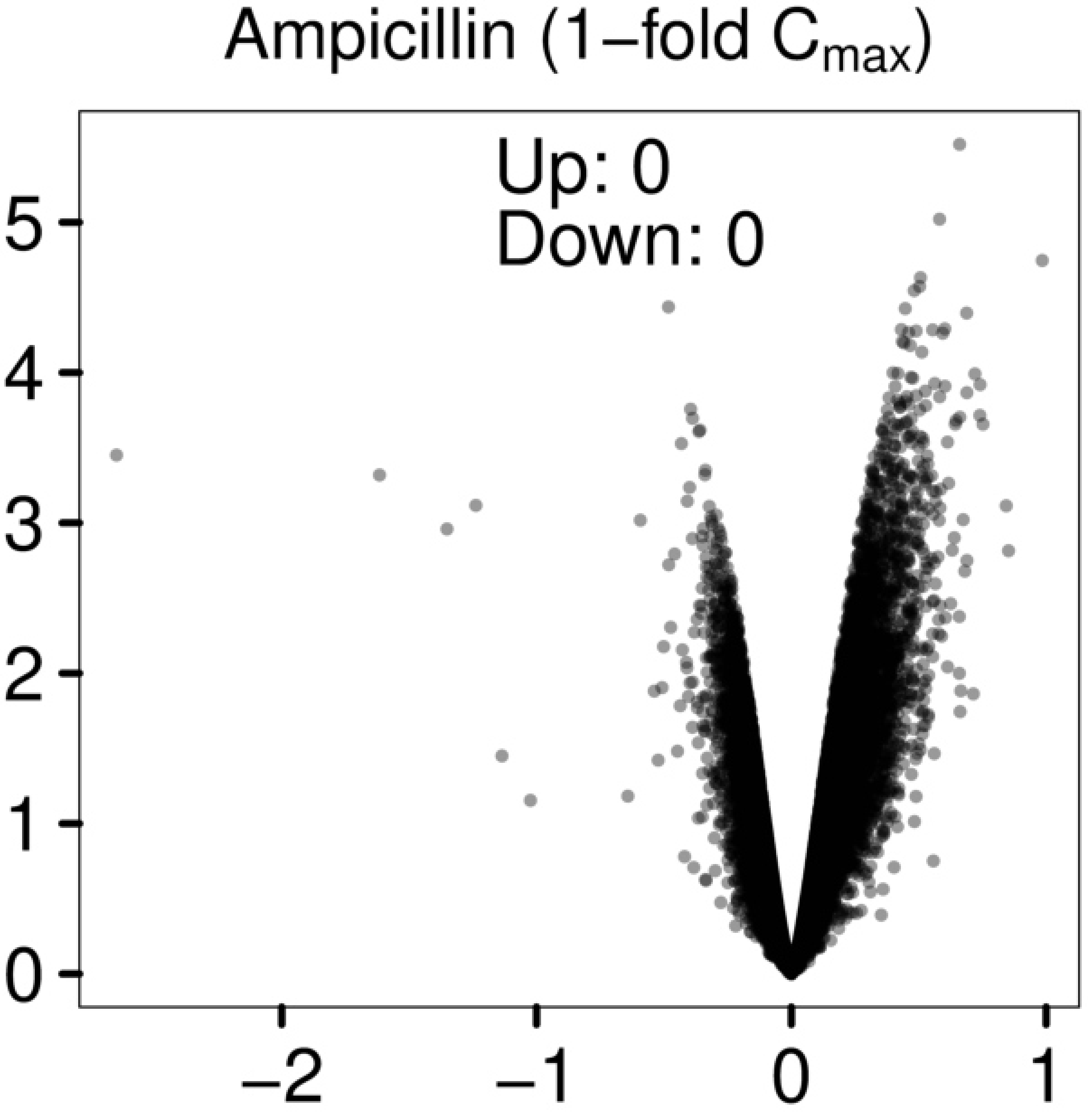
Ampicillin (1–fold C_{max})
Up: 0
Down: 0
5
4
3
2
1
0
–2
–1
0
1

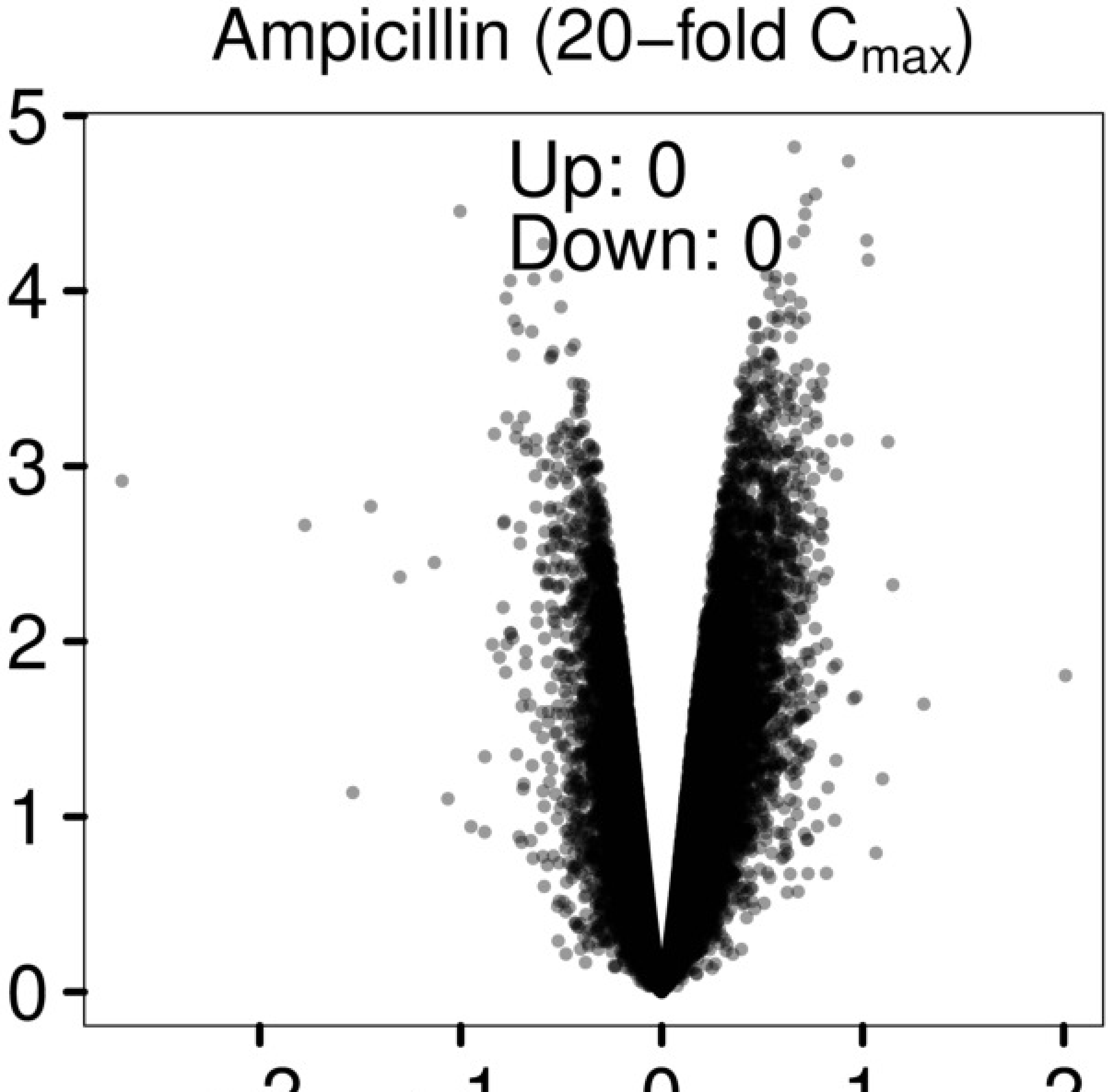
Ampicillin (20–fold C$_{max}$)
Up: 0
Down: 0
5
4
3
2
1
0
–2
–1
0
1
2

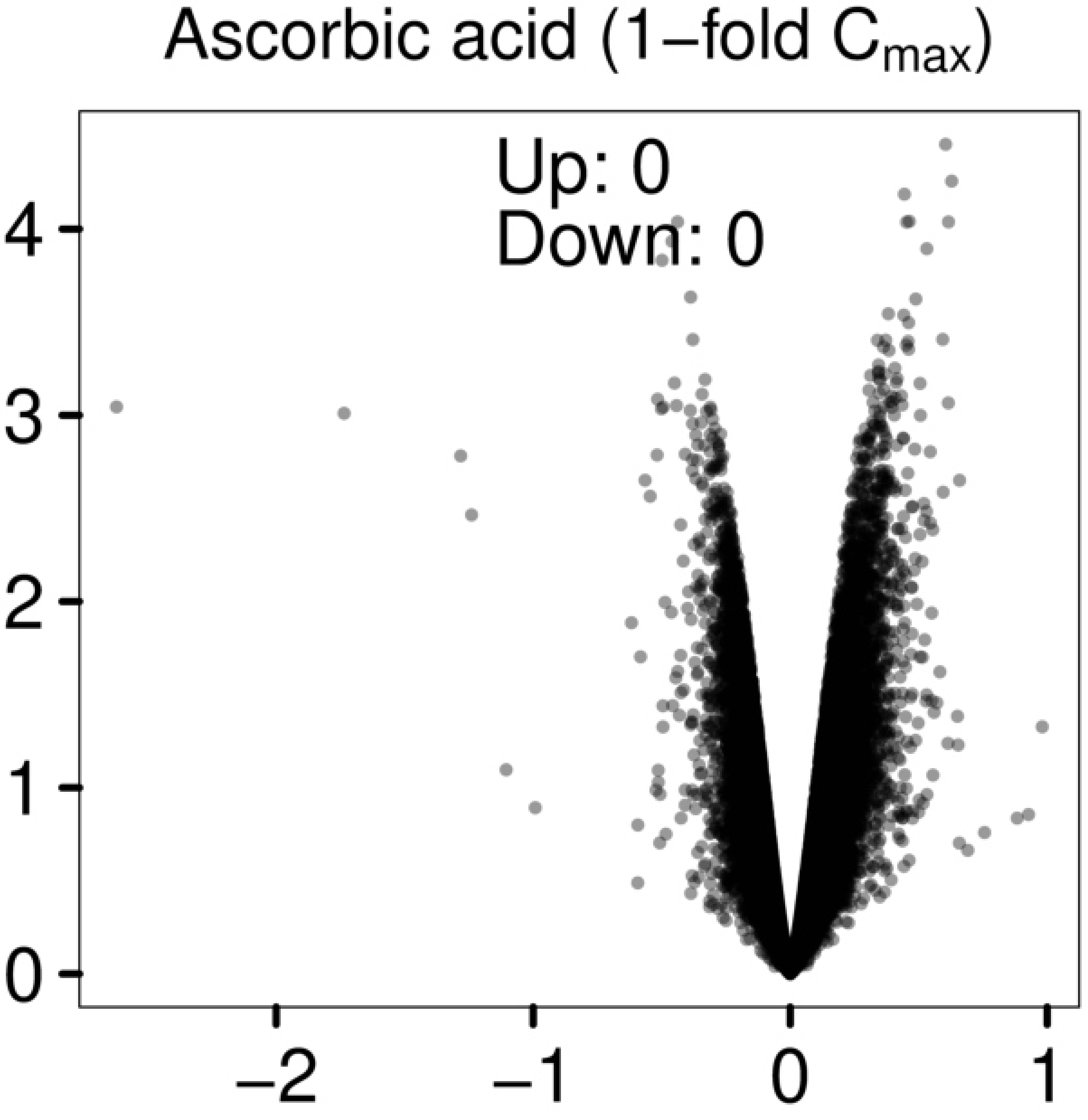
Ascorbic acid (1−fold C_{max})
Up: 0
Down: 0
4
3
2
1
0
−2
−1
0
1

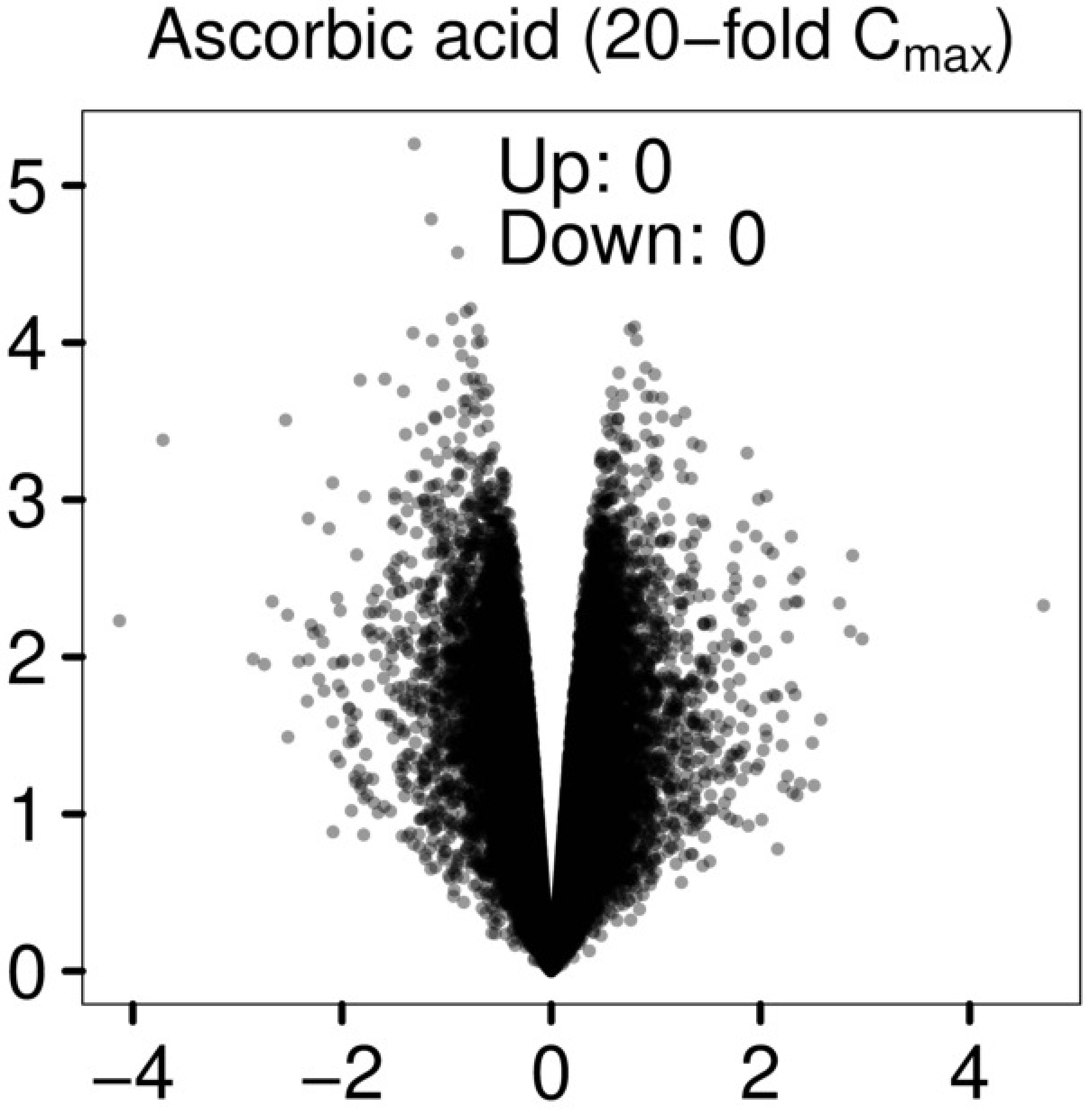

Ascorbic acid (20–fold C$_{max}$)
Up: 0
Down: 0
5
4
3
2
1
0
–4
–2
0
2
4

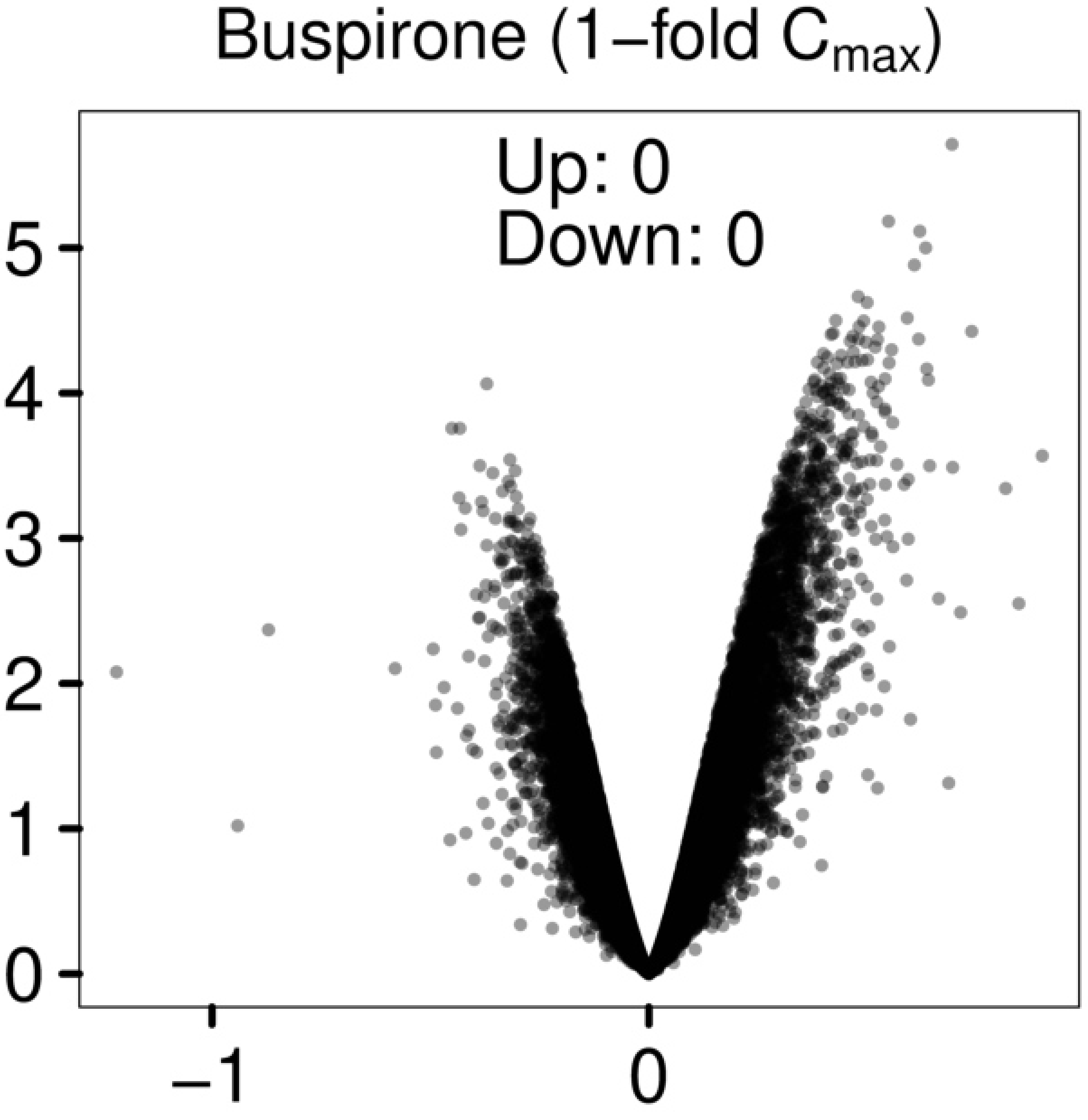

Buspirone (1–fold C_{max})
Up: 0
Down: 0
5
4
3
2
1
0
–1
0

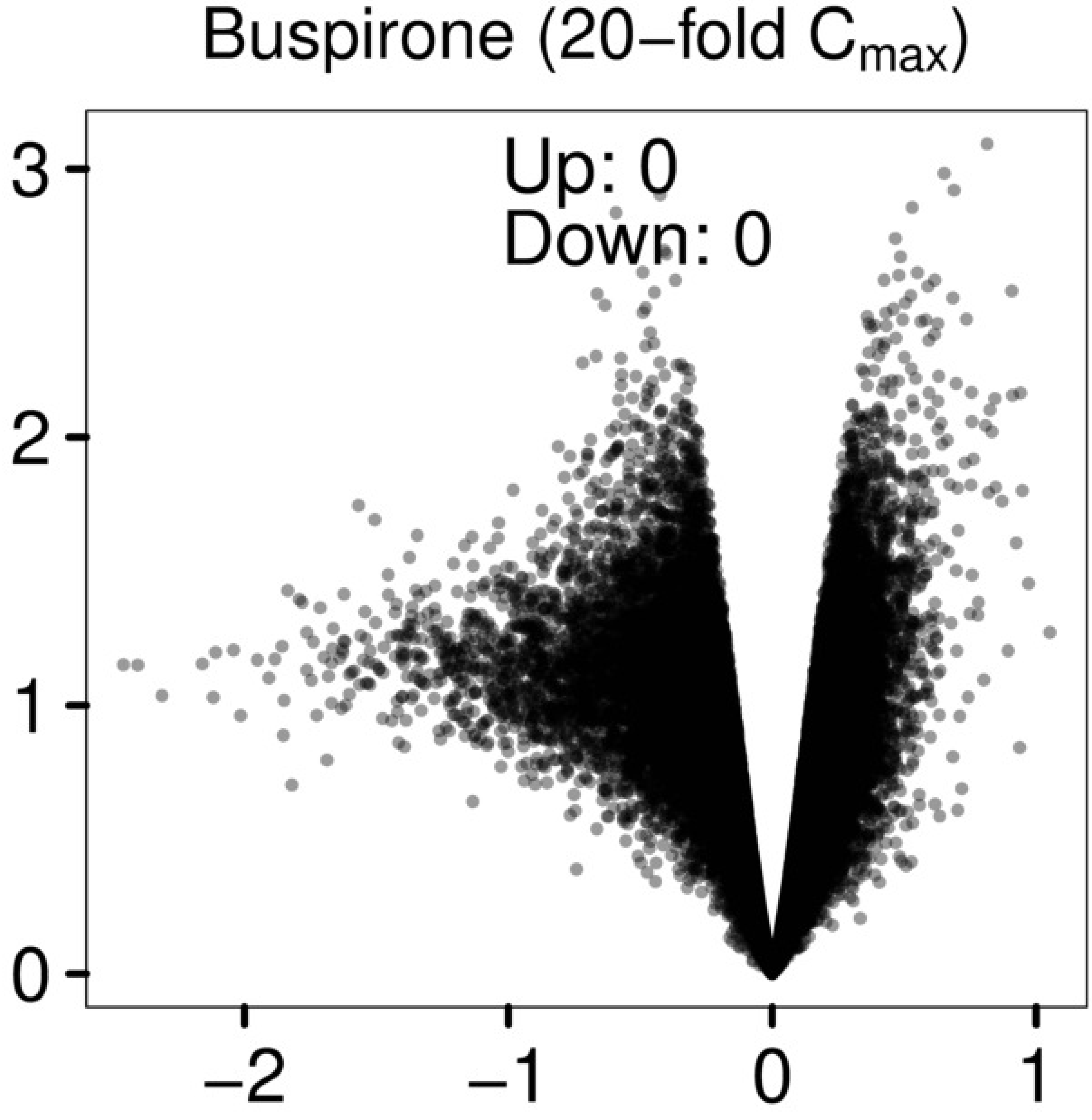
Buspirone (20−fold C_{max})
Up: 0
Down: 0
3
2
1
0
−2
−1
0
1

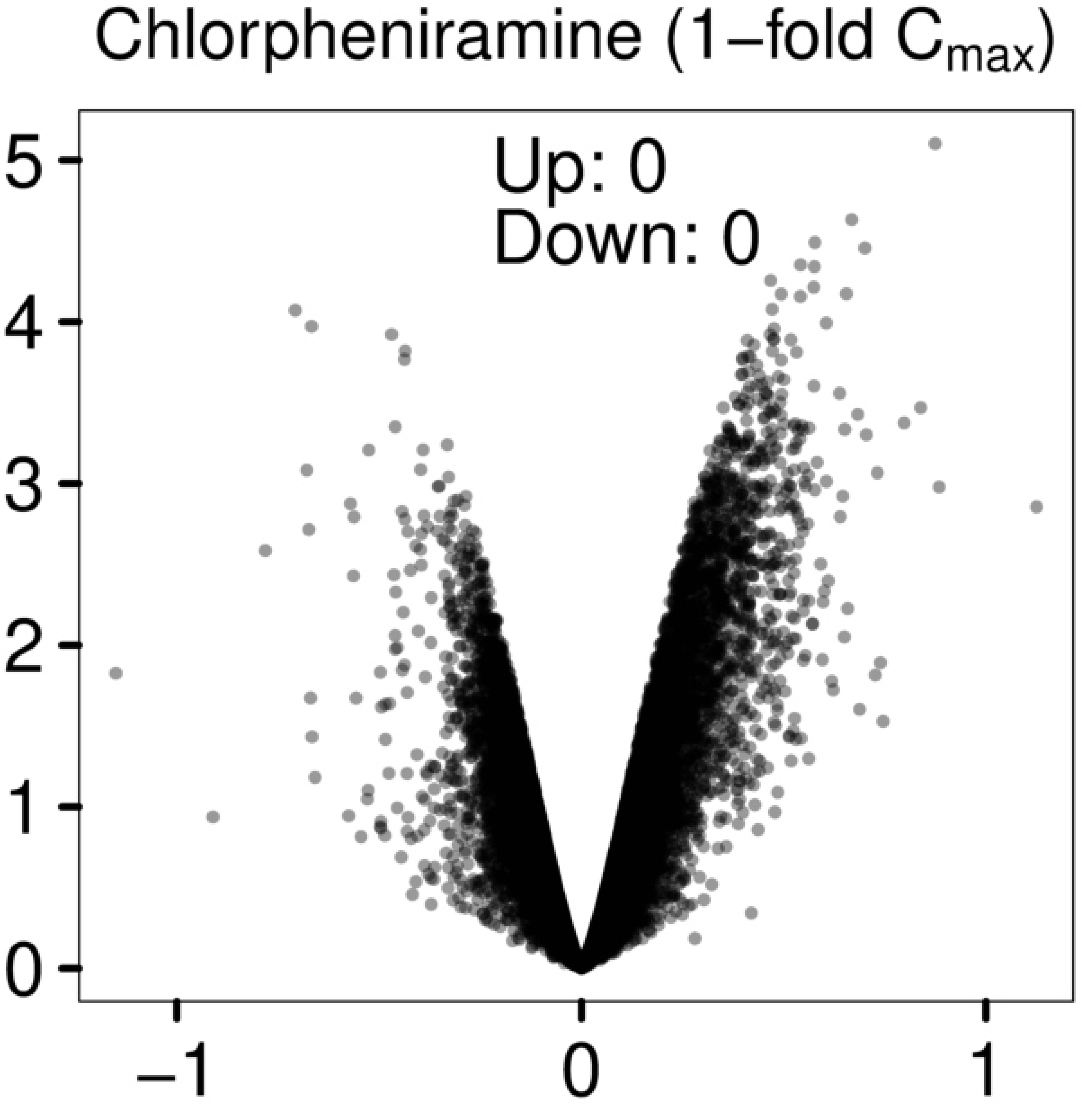
Chlorpheniramine (1–fold C_{max})
Up: 0
Down: 0
5
4
3
2
1
0
–1
0
1

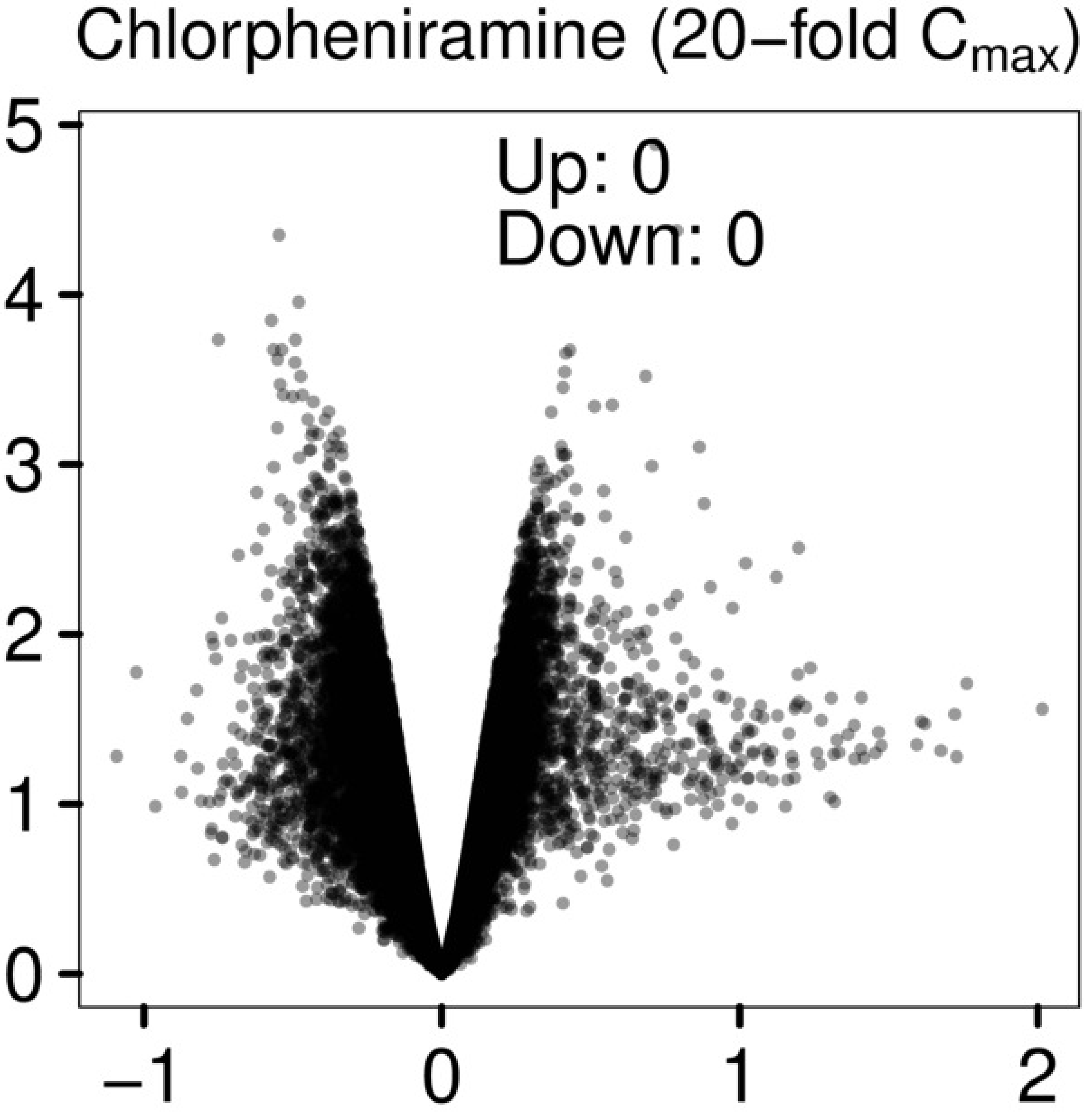
Chlorpheniramine (20–fold C_{max})
Up: 0
Down: 0
5
4
3
2
1
0
–1
0
1
2

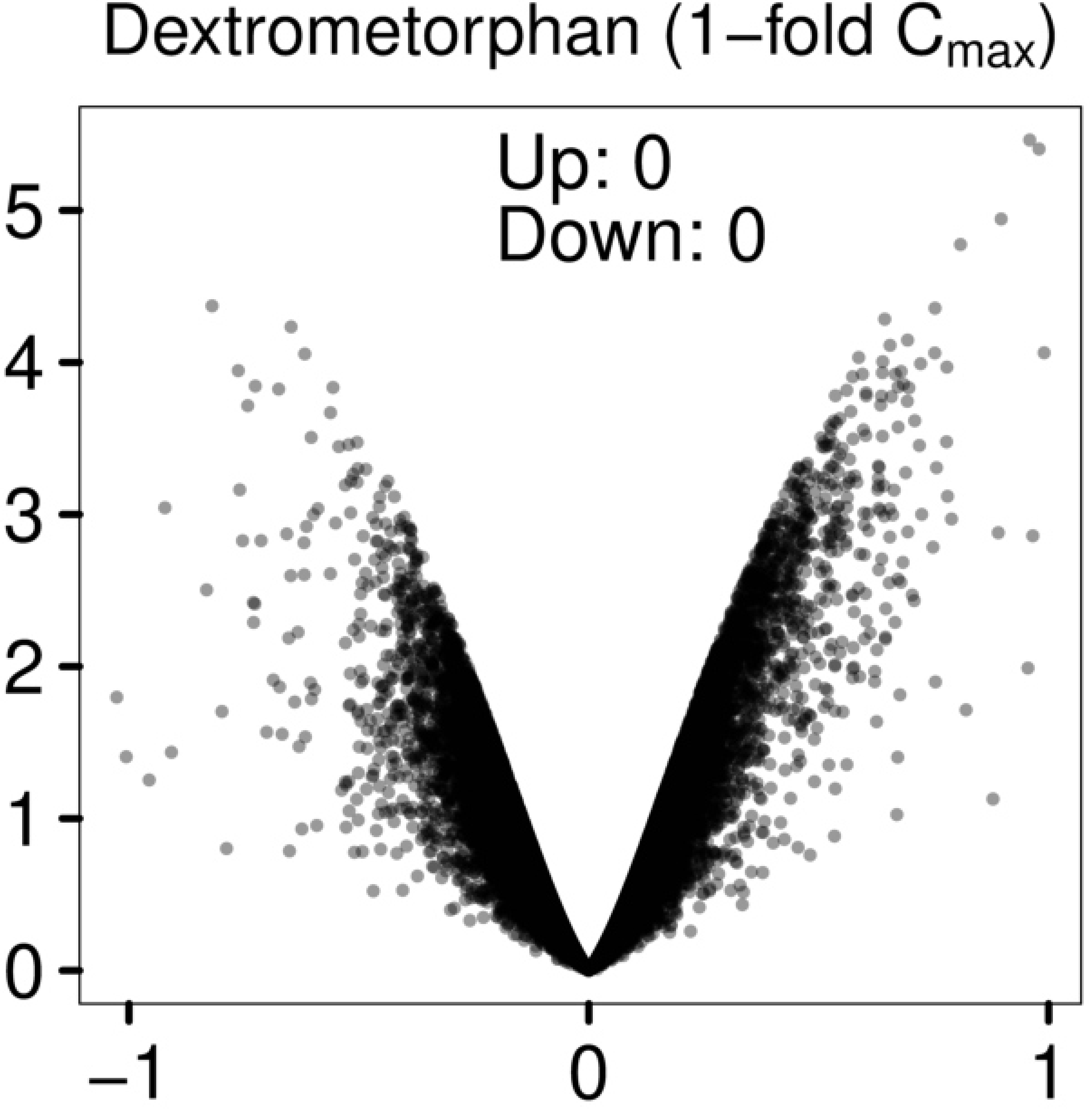

Dextrometorphan (1−fold C_{max})
Up: 0
Down: 0
5
4
3
2
1
0
−1
0
1

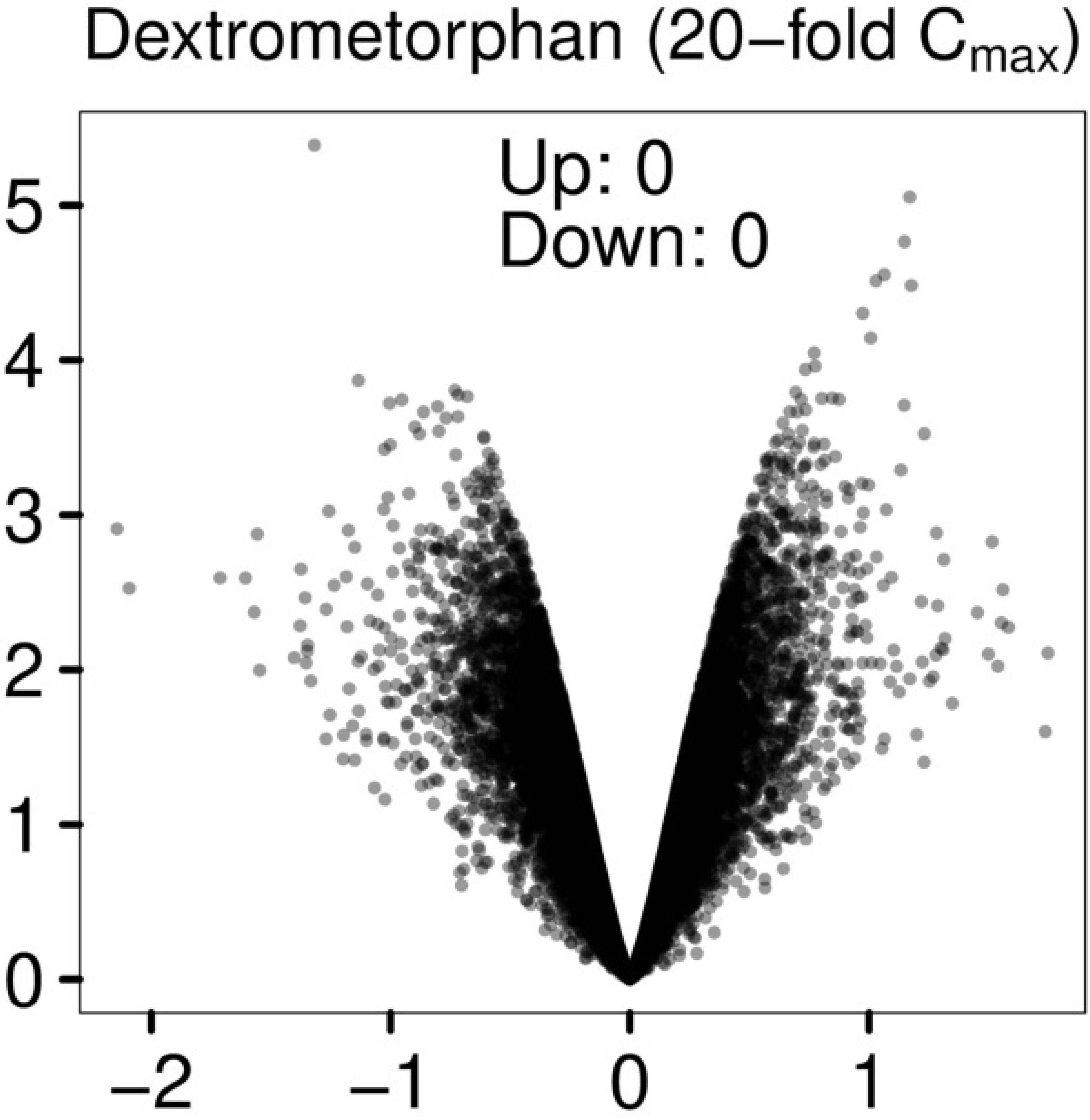
Dextrometorphan (20–fold C_{max})
Up: 0
Down: 0
5
4
3
2
1
0
–2
–1
0
1

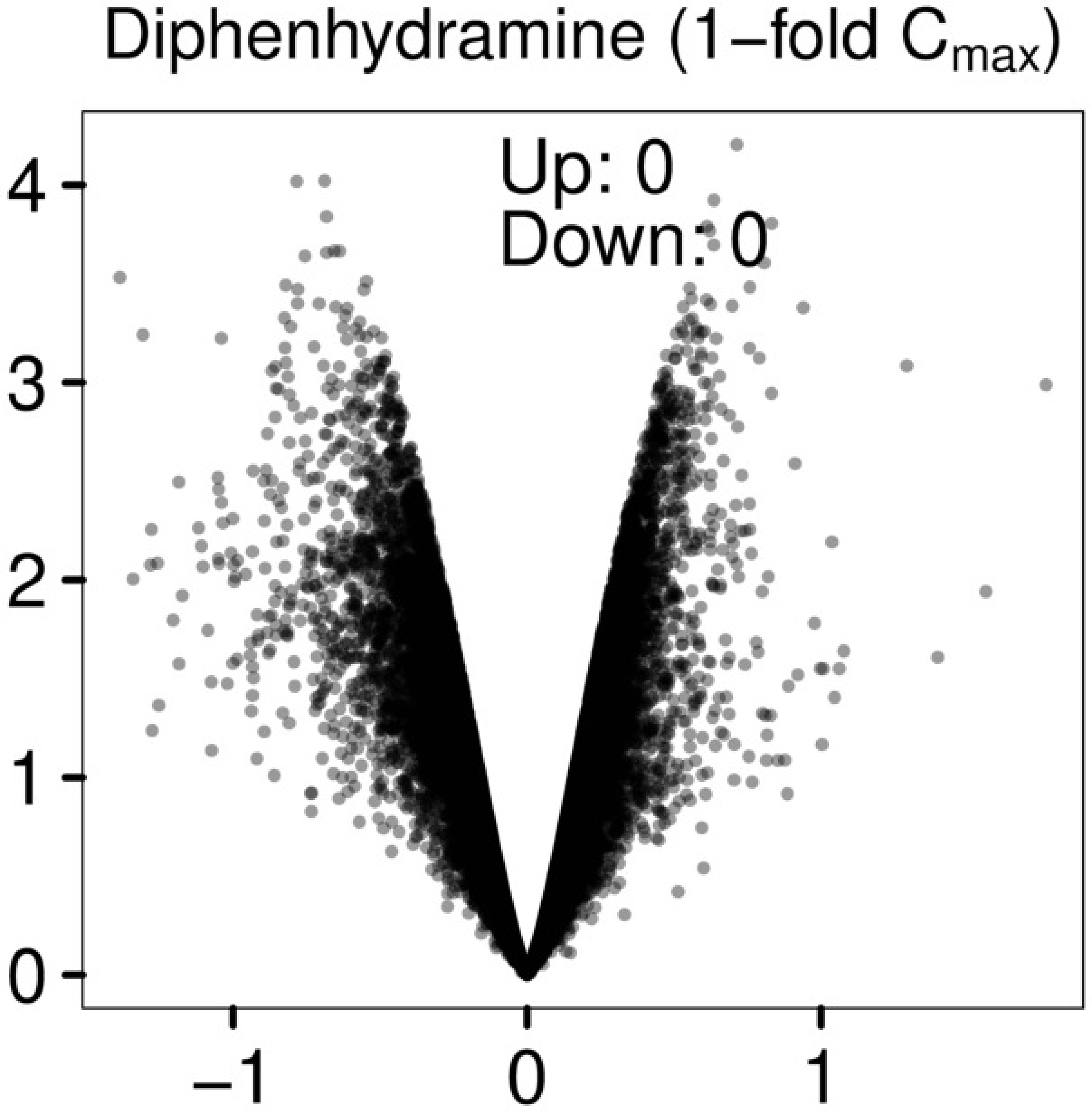
Diphenhydramine (1–fold C_{max})
Up: 0
Down: 0
4
3
2
1
0
–1
0
1

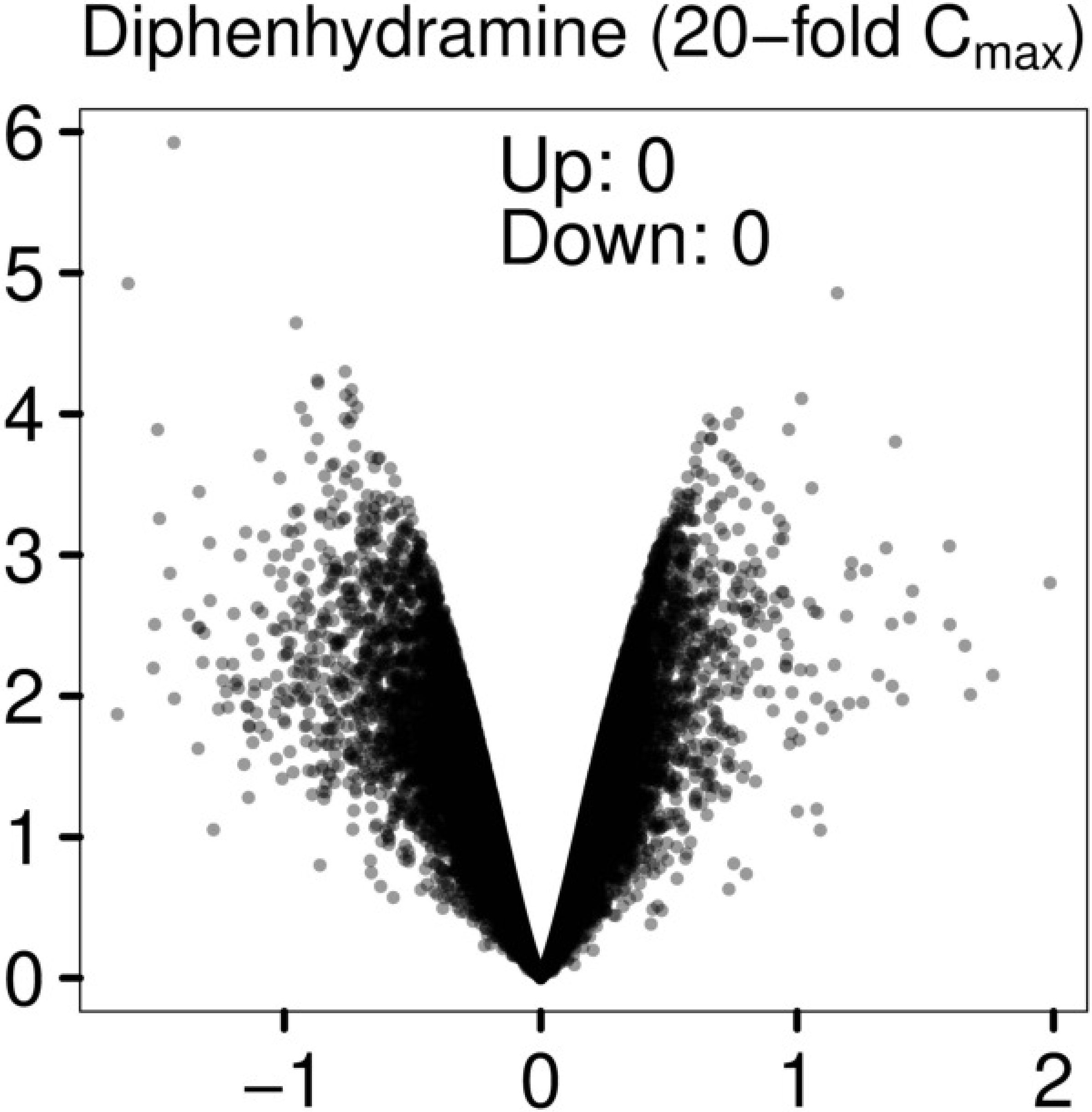

Diphenhydramine (20–fold C$_{max}$)
Up: 0
Down: 0
6
5
4
3
2
1
0
–1
0
1
2

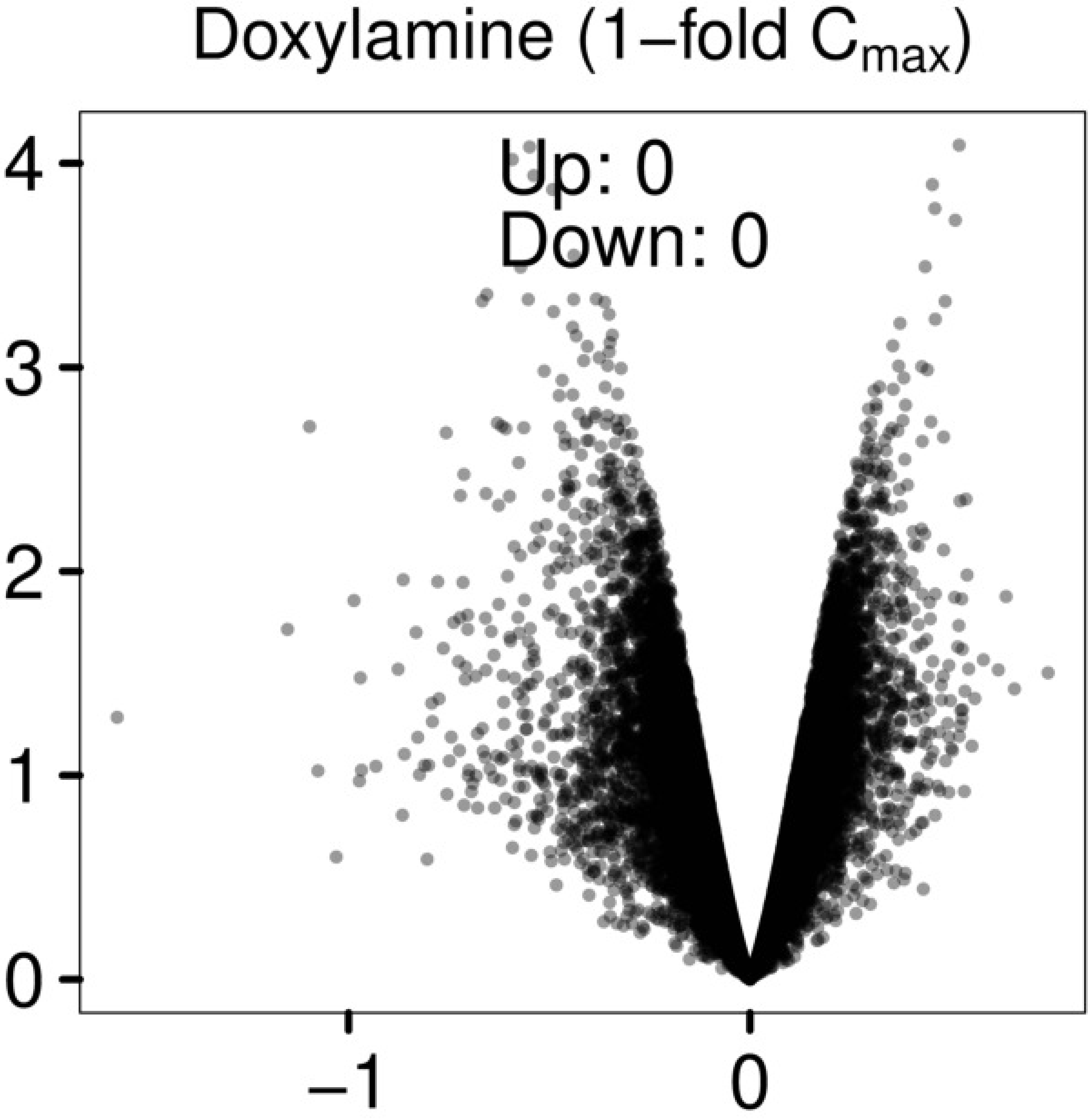
Doxylamine (1–fold Cmax)
Up: 0
Down: 0
4
3
2
1
0
–1
0

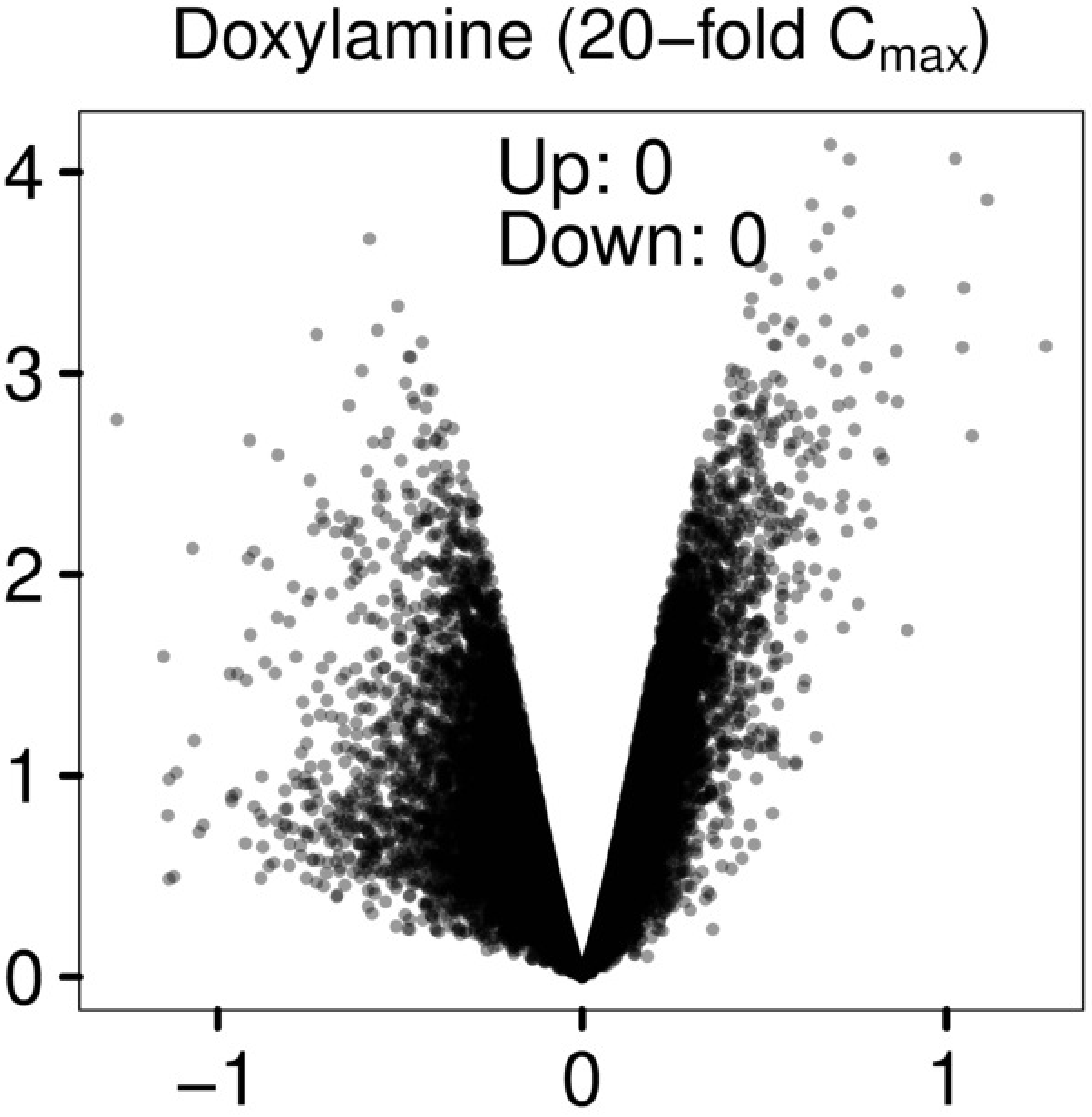

Doxylamine (20–fold Cmax)
Up: 0
Down: 0
4
3
2
1
0
–1
0
1

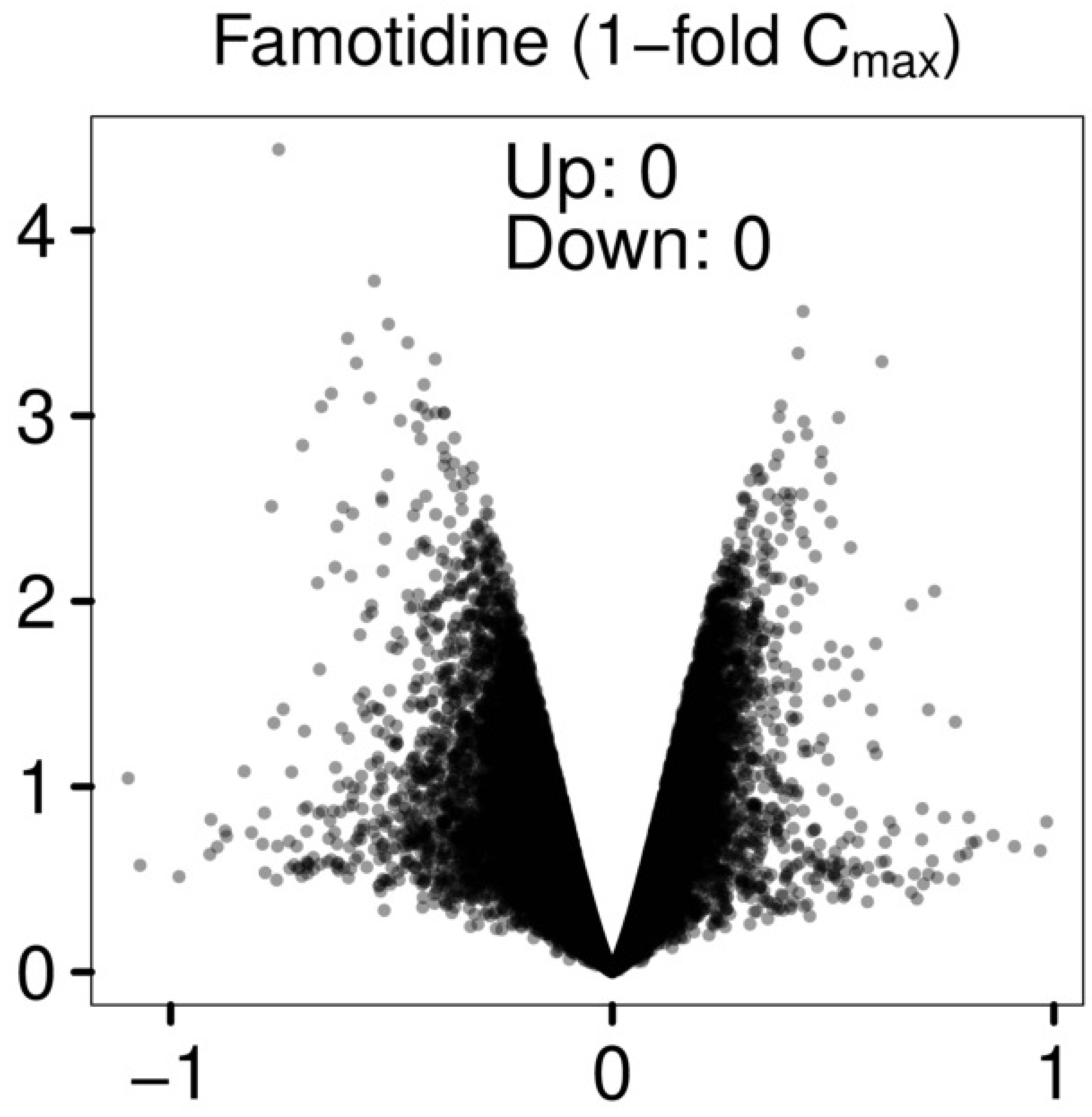
Famotidine (1−fold C_{max})
Up: 0
Down: 0
4
3
2
1
0
−1
0
1

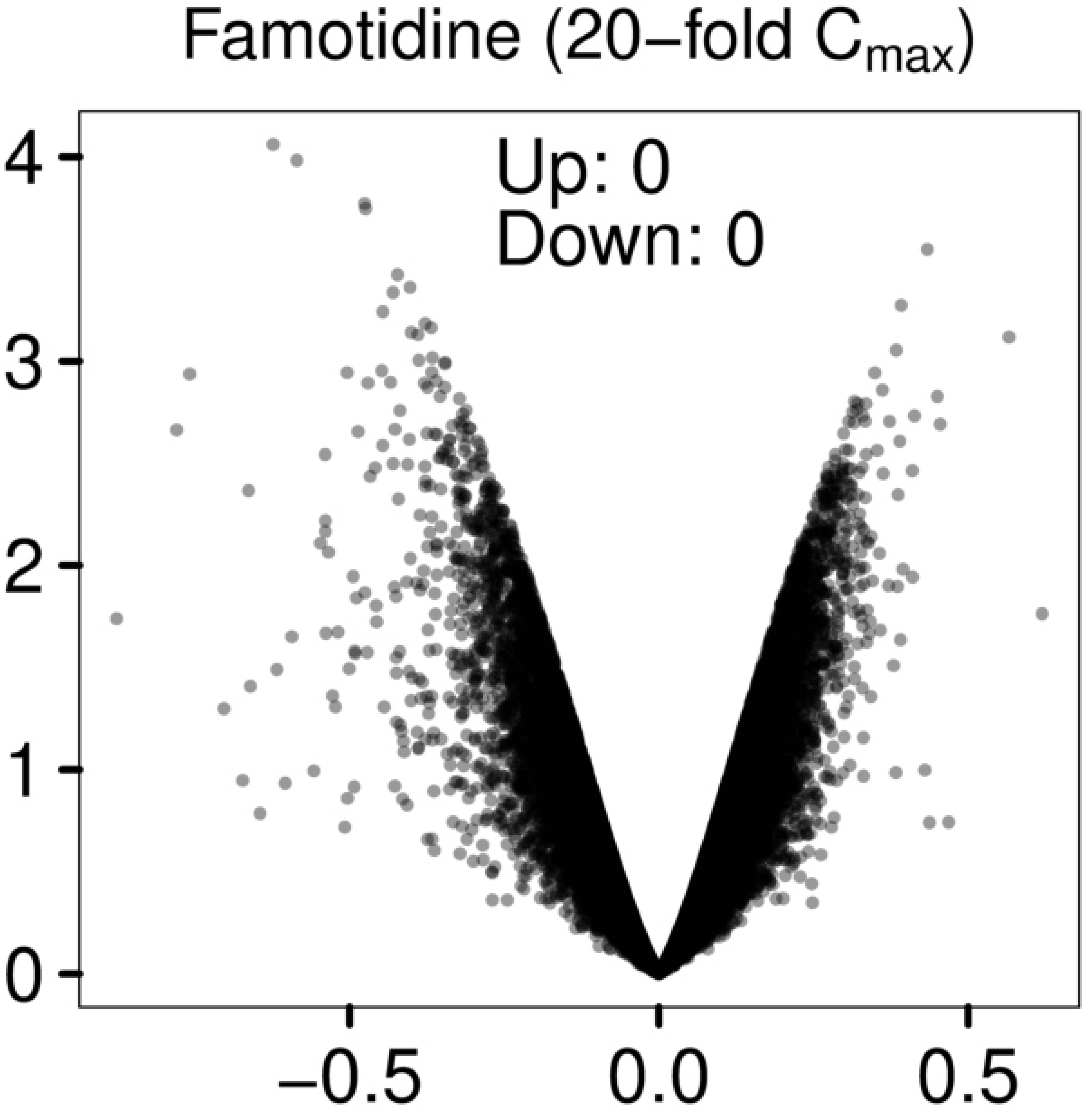
Famotidine (20−fold C_{max})
Up: 0
Down: 0
4
3
2
1
0
−0.5
0.0
0.5

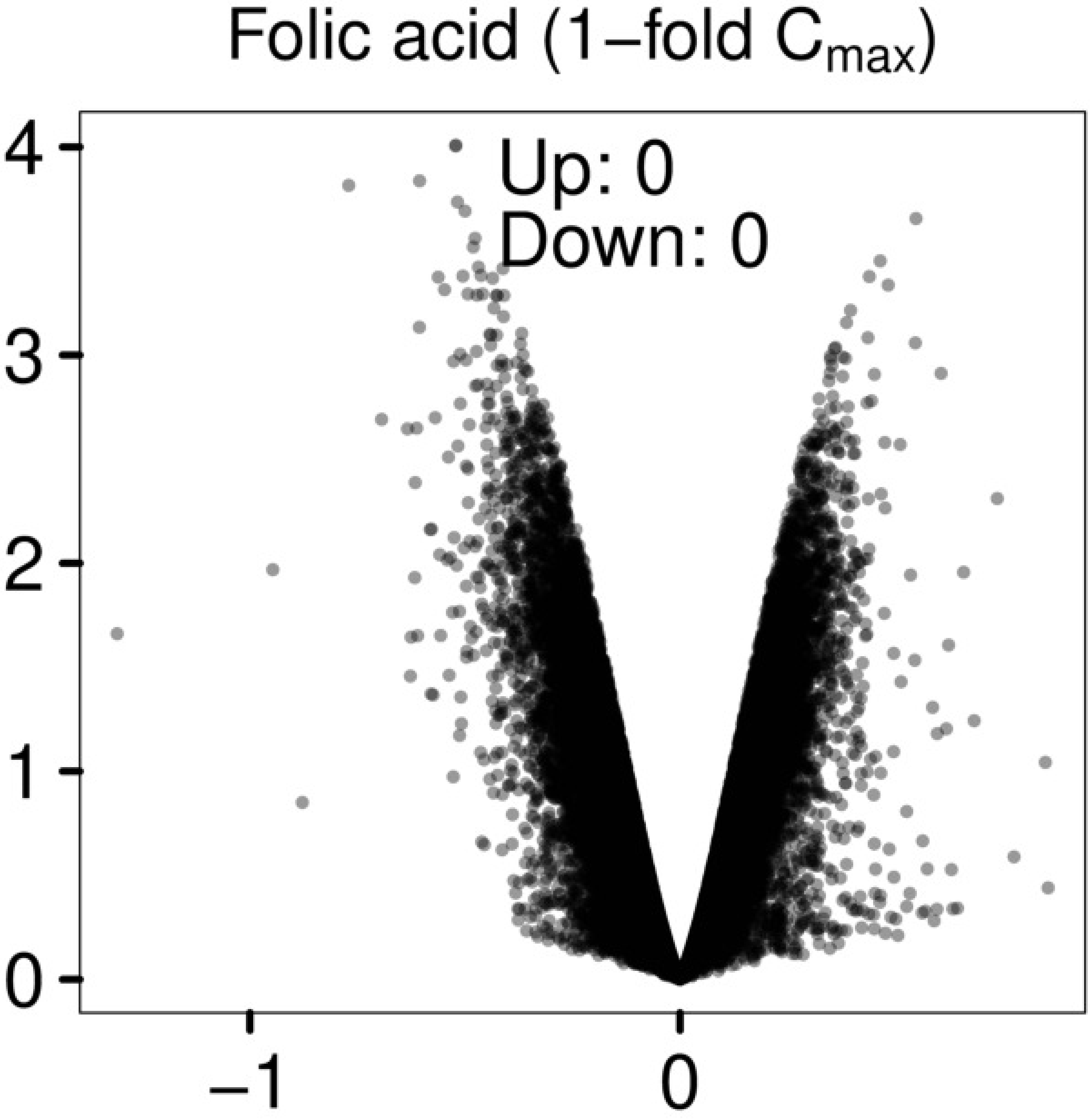
Folic acid (1–fold C_{max})
Up: 0
Down: 0
4
3
2
1
0
–1
0

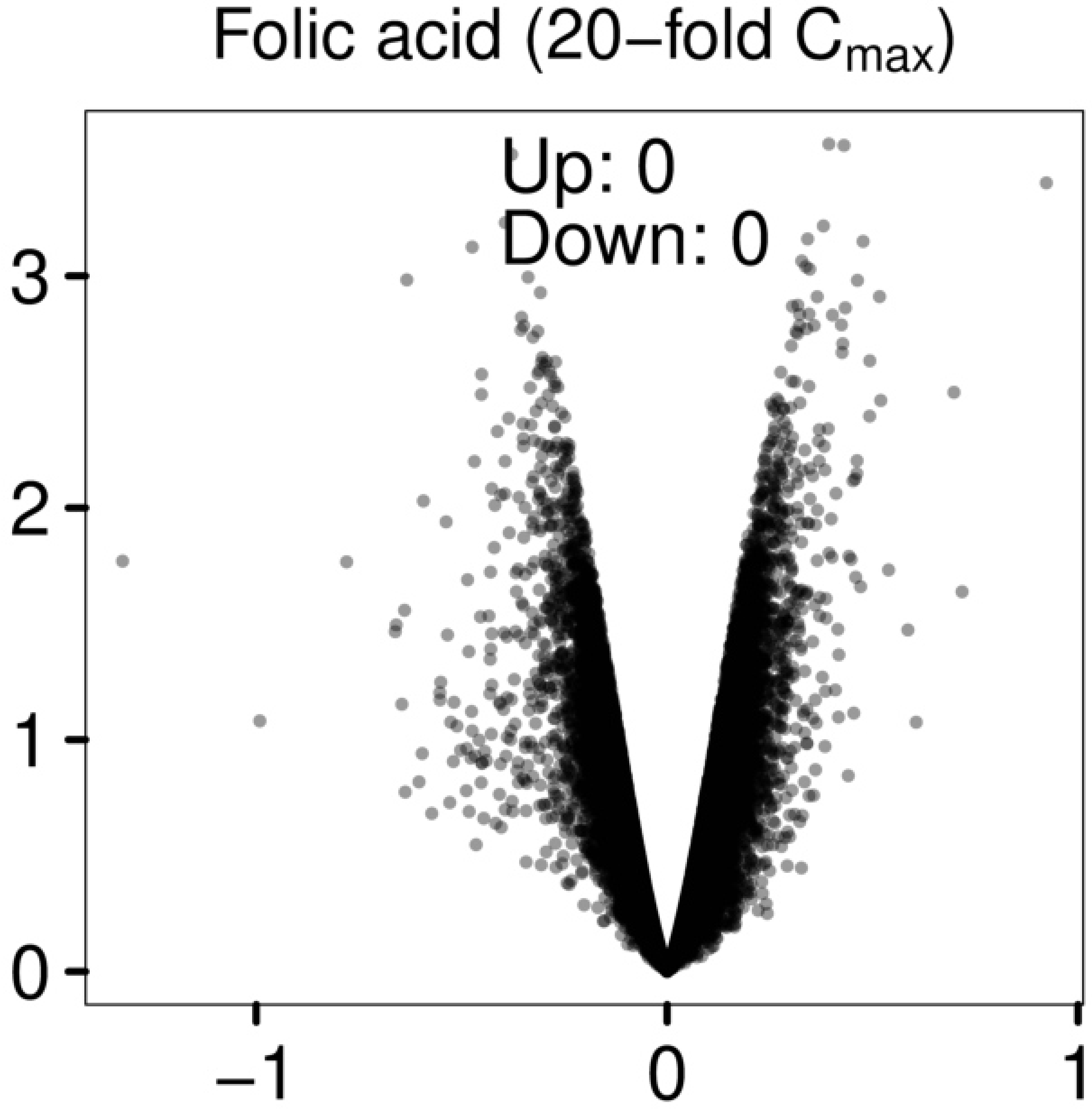
Folic acid (20−fold C_{max})
Up: 0
Down: 0
3
2
1
0
−1
0
1

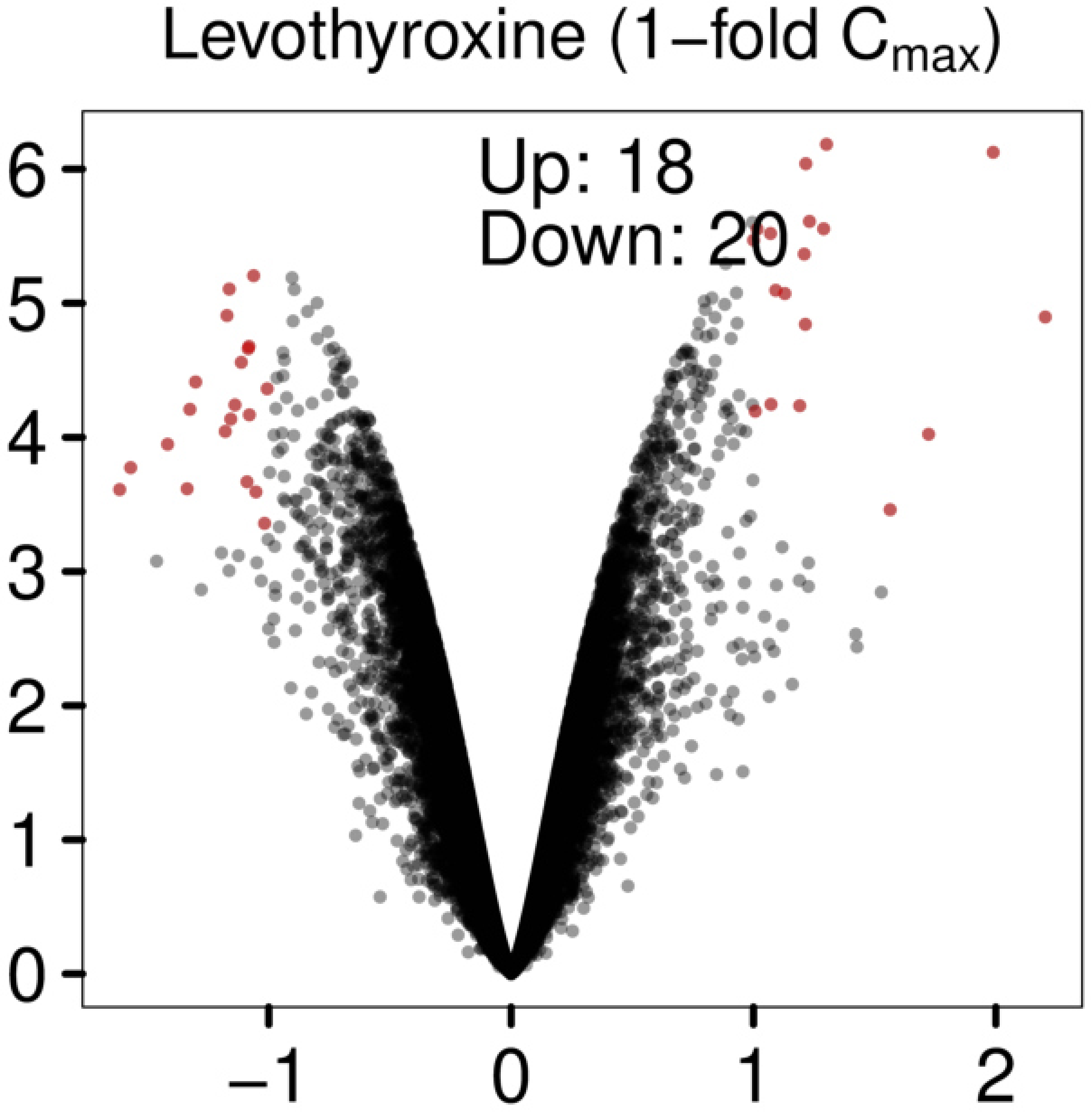
Levothyroxine (1–fold Cmax)
Up: 18
Down: 20
6
5
4
3
2
1
0
–1
0
1
2

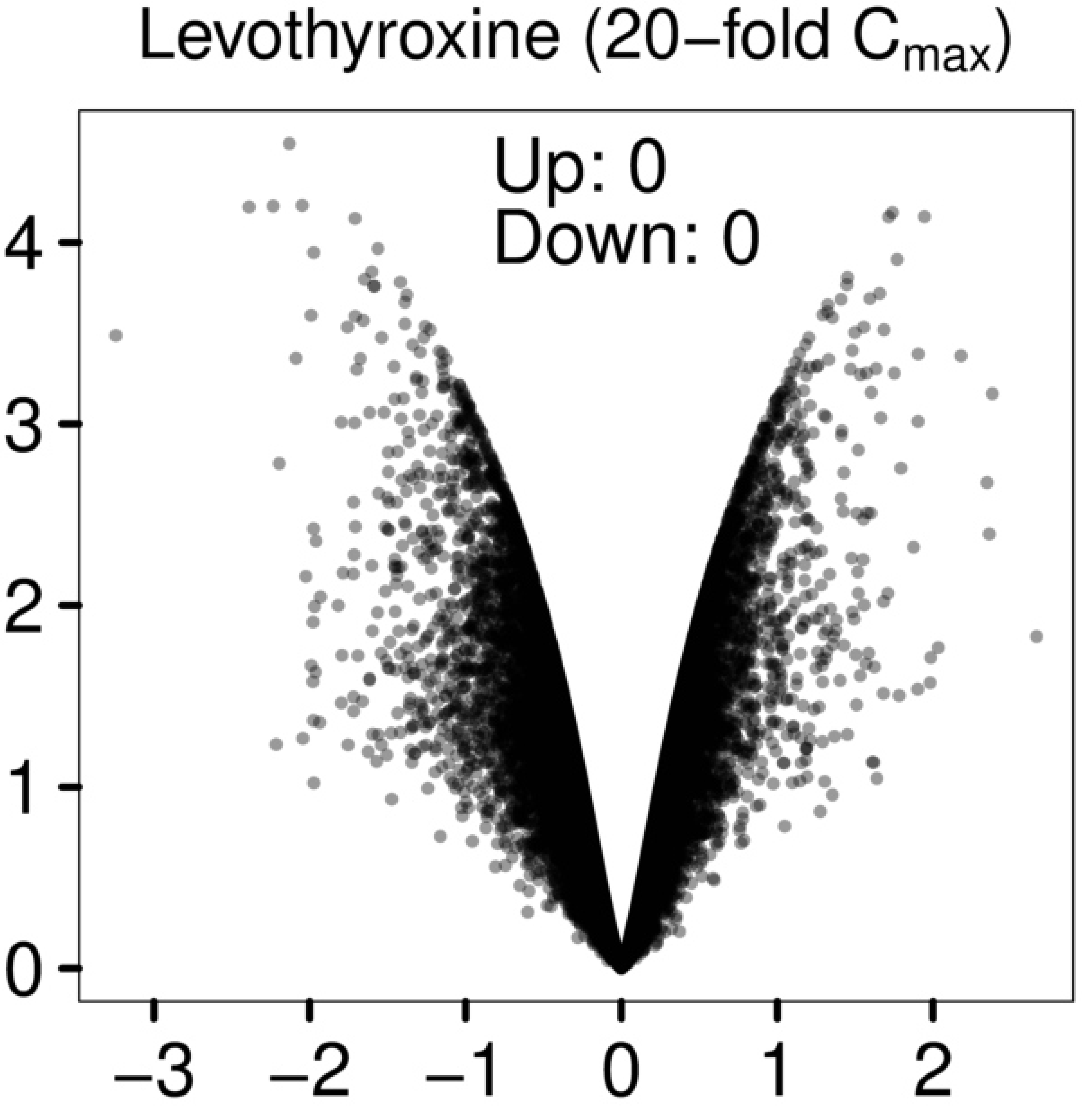
Levothyroxine (20–fold C_{max})
Up: 0
Down: 0
4
3
2
1
0
–3
–2
–1
0
1
2

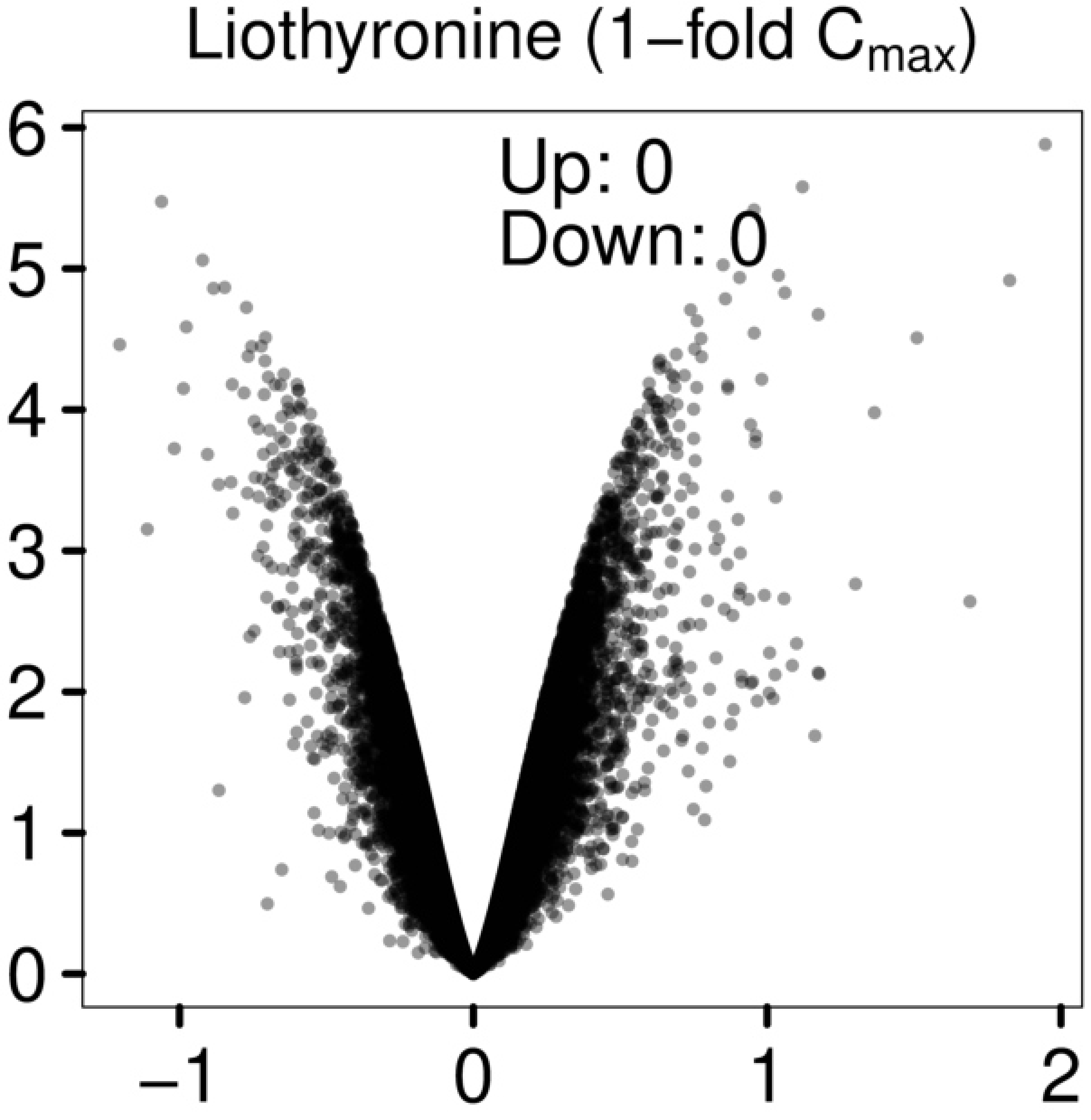

Liothyronine (1−fold C_{max})
Up: 0
Down: 0
6
5
4
3
2
1
0
−1
0
1
2

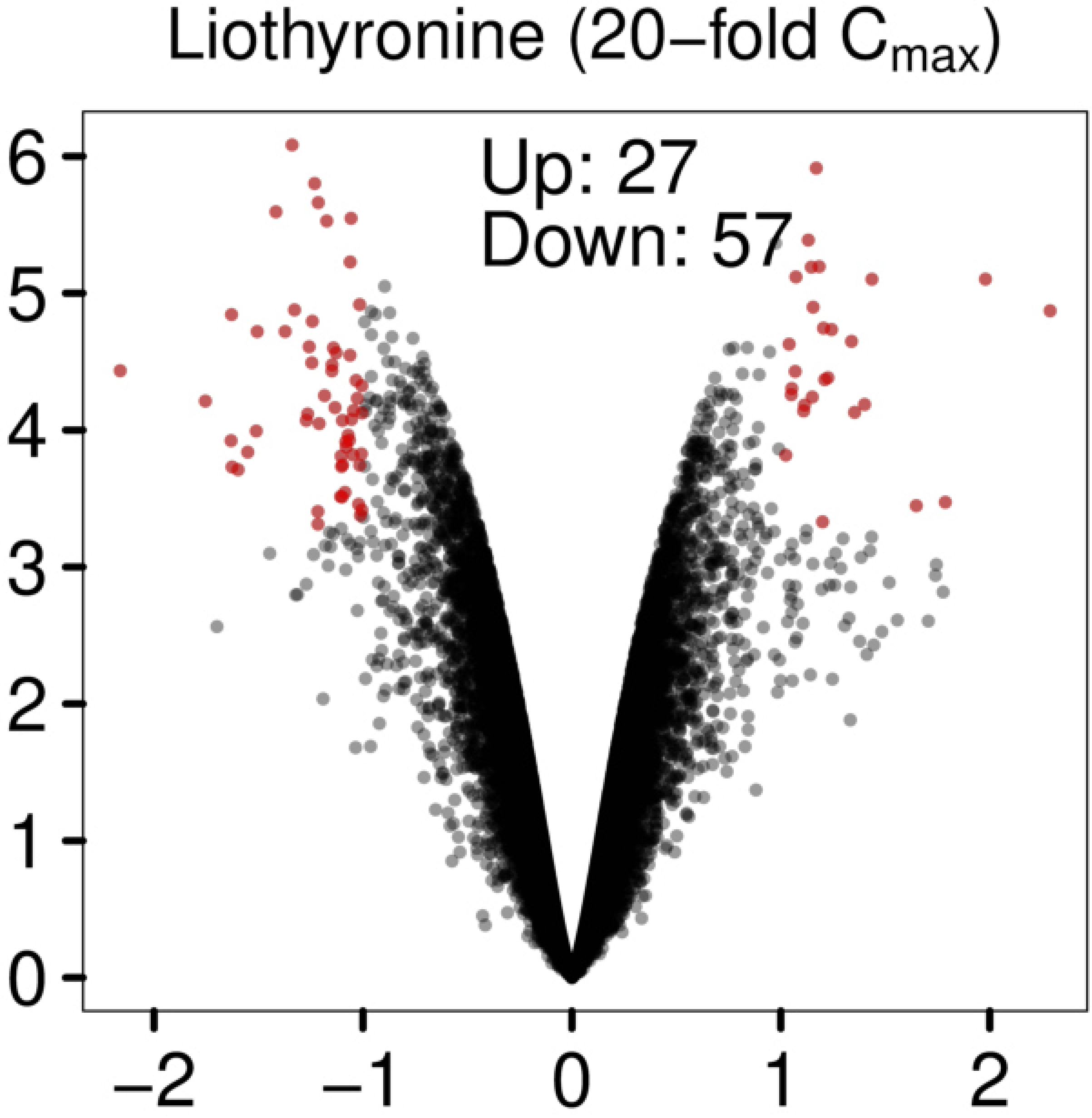
Liothyronine (20–fold C$_{max}$)
Up: 27
Down: 57
6
5
4
3
2
1
0
−2
−1
0
1
2

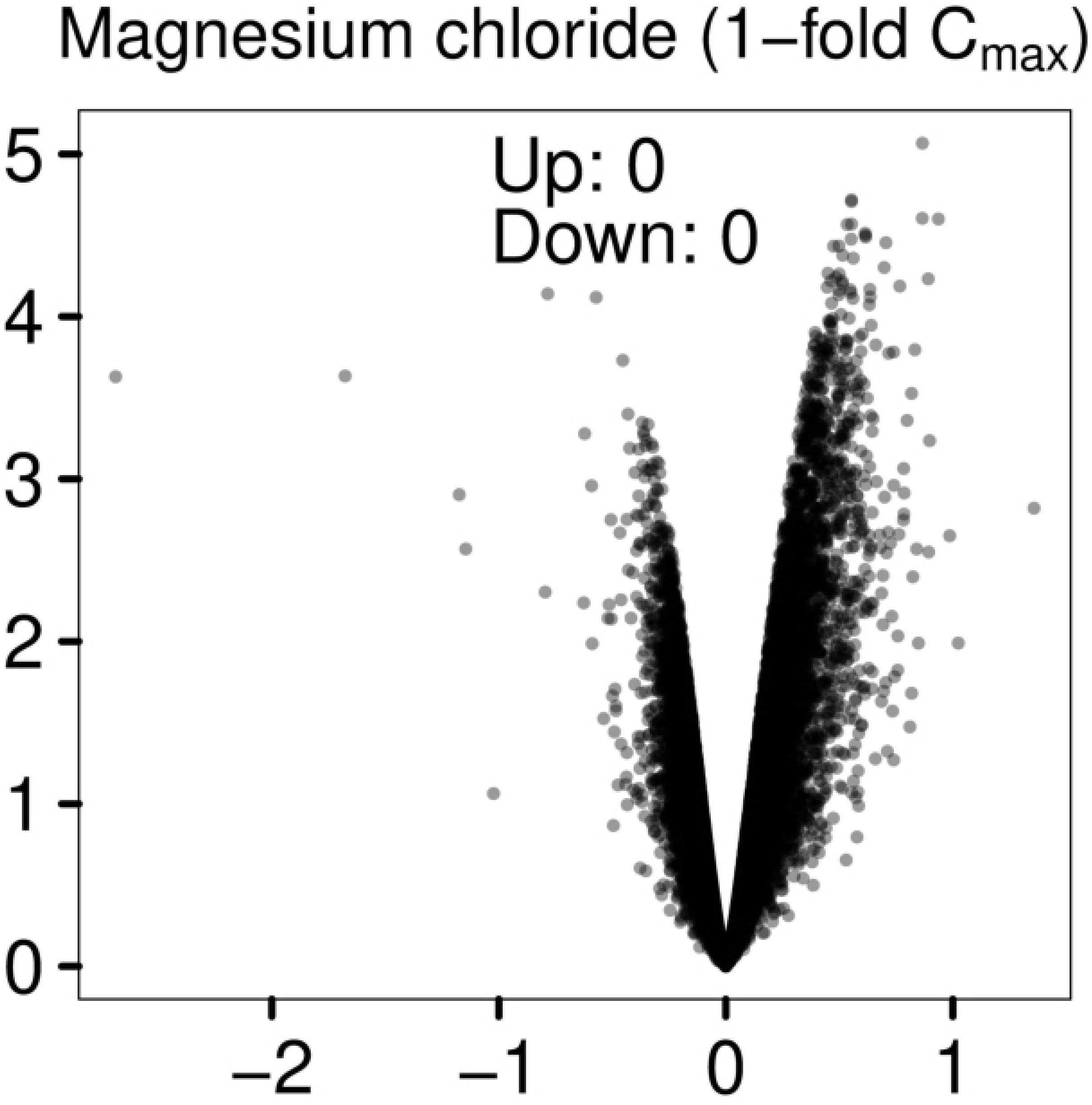
Magnesium chloride (1−fold C_{max})
Up: 0
Down: 0
5
4
3
2
1
0
−2
−1
0
1

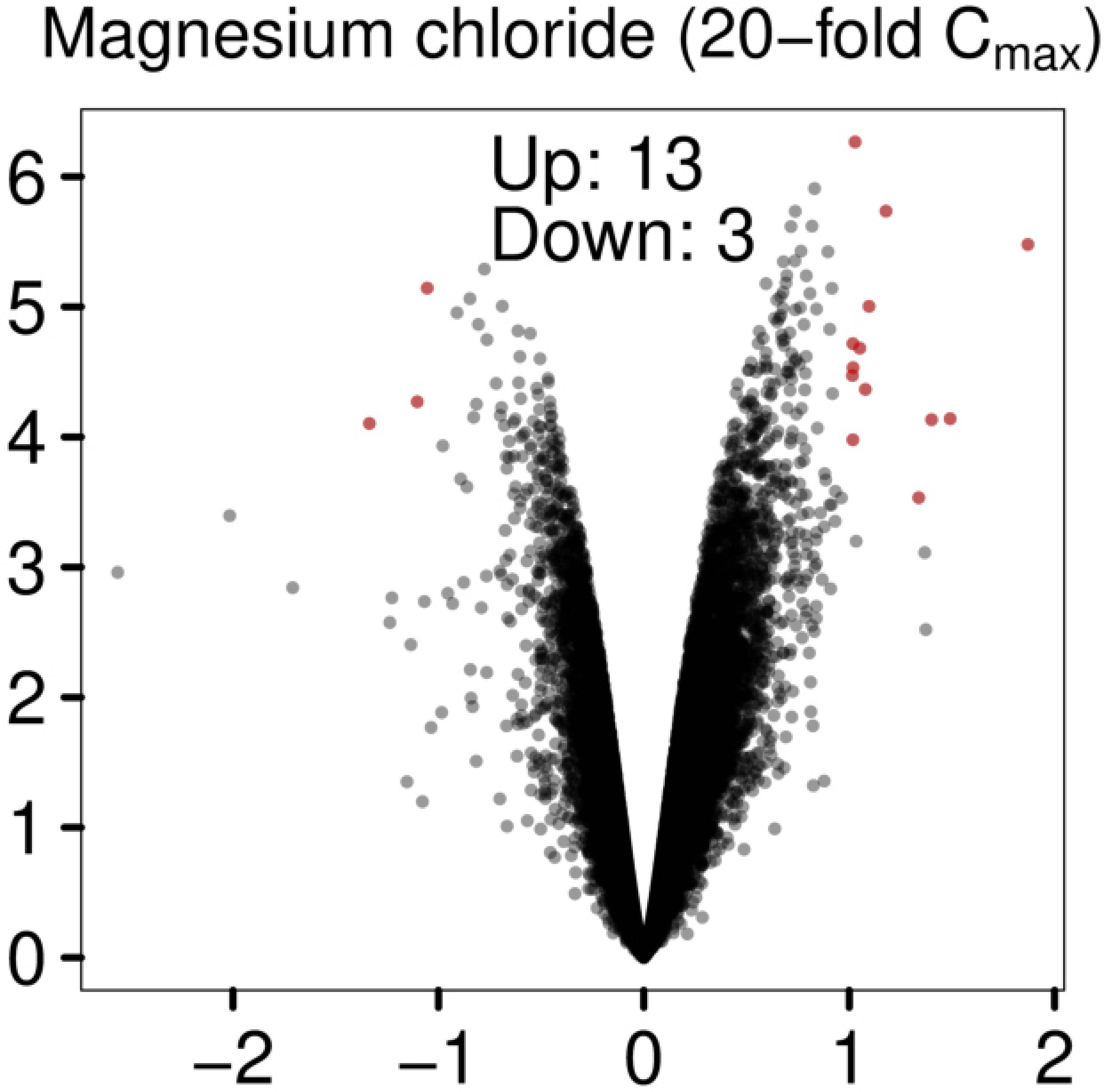
Magnesium chloride (20–fold C_{max})
Up: 13
Down: 3
6
5
4
3
2
1
0
–2
–1
0
1
2

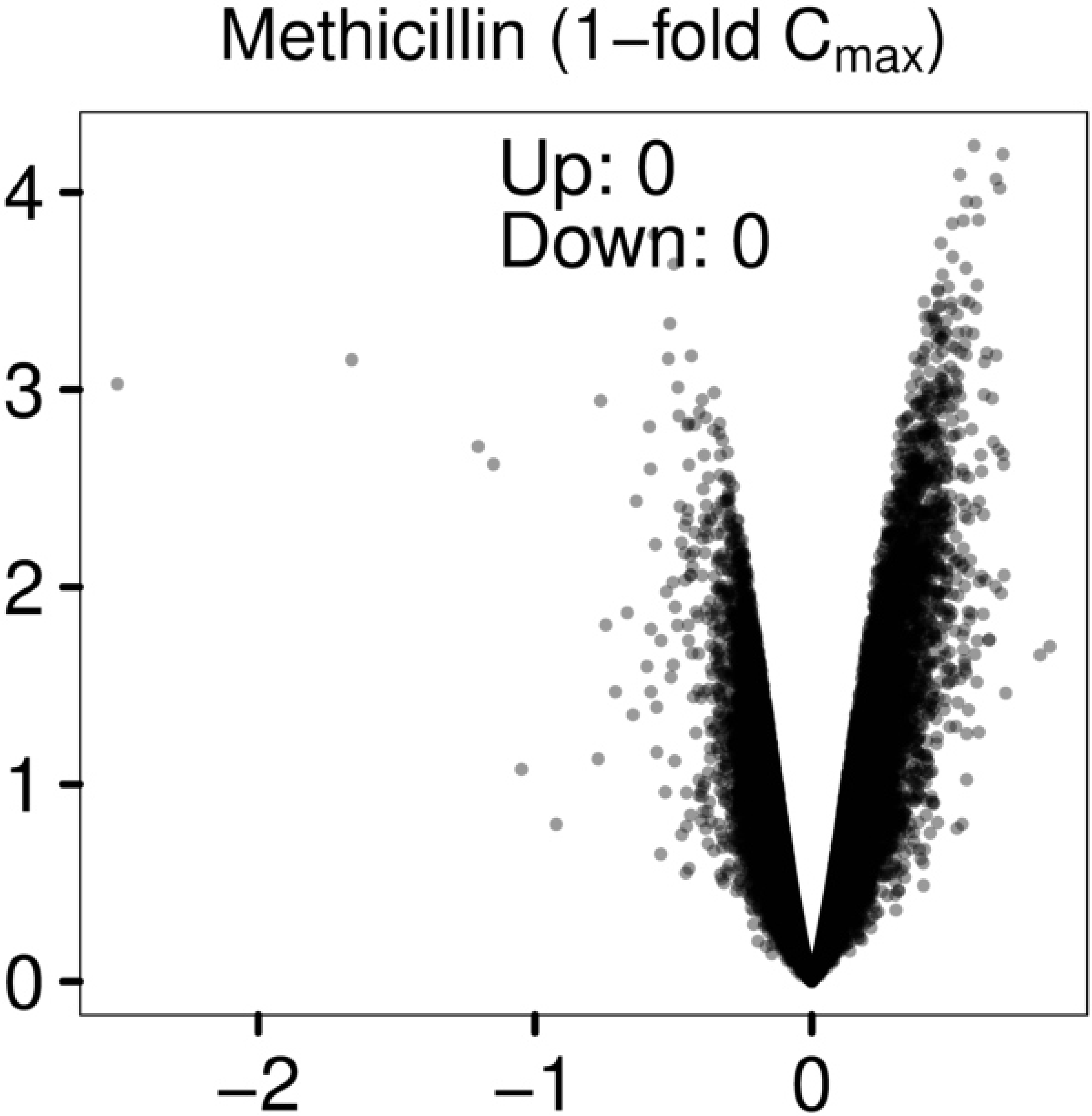
Methicillin (1−fold C_{max})
Up: 0
Down: 0
4
3
2
1
0
−2
−1
0

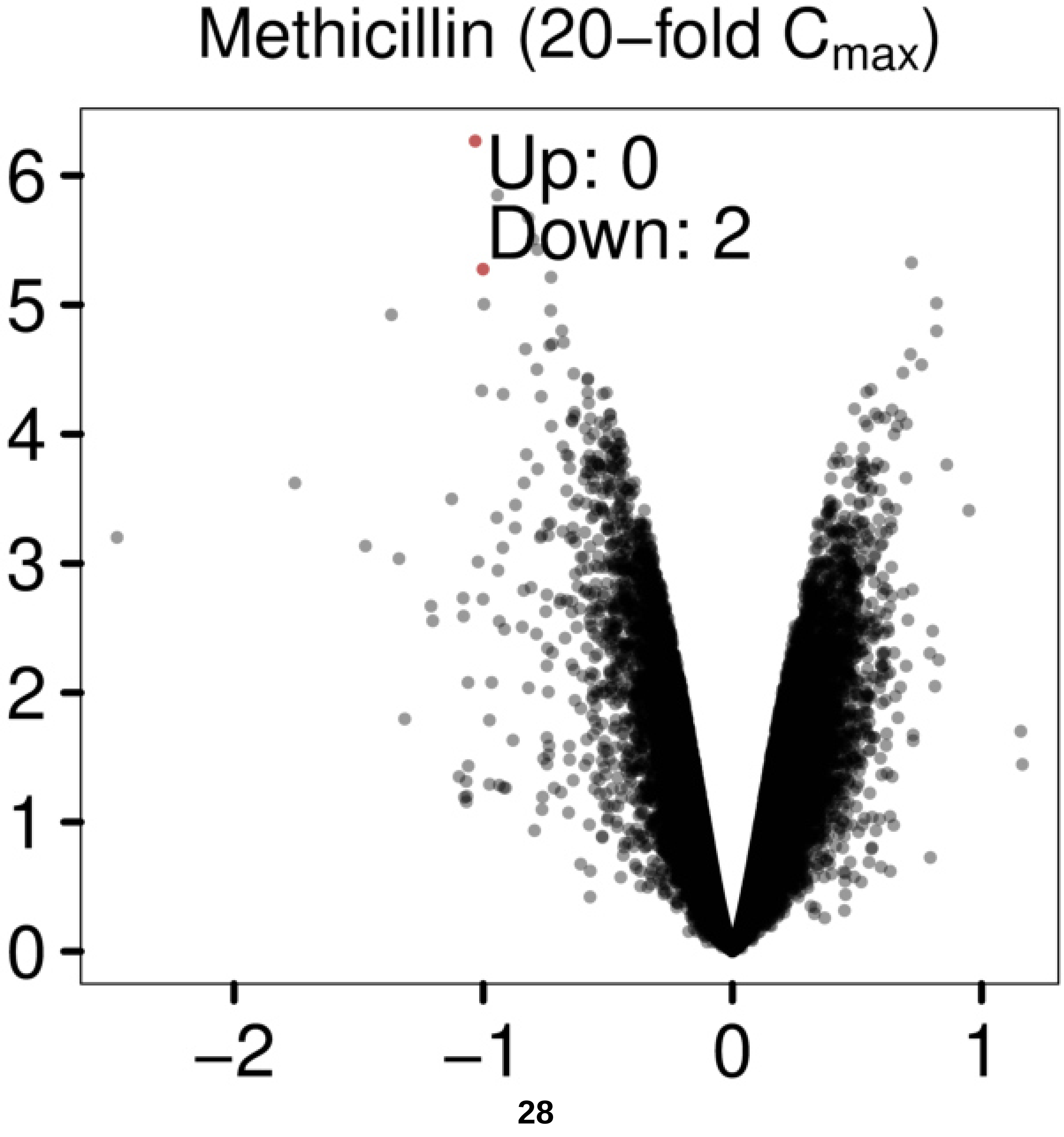

Methicillin (20–fold C_{max})
Up: 0
Down: 2
6
5
4
3
2
1
0
–2
–1
0
1

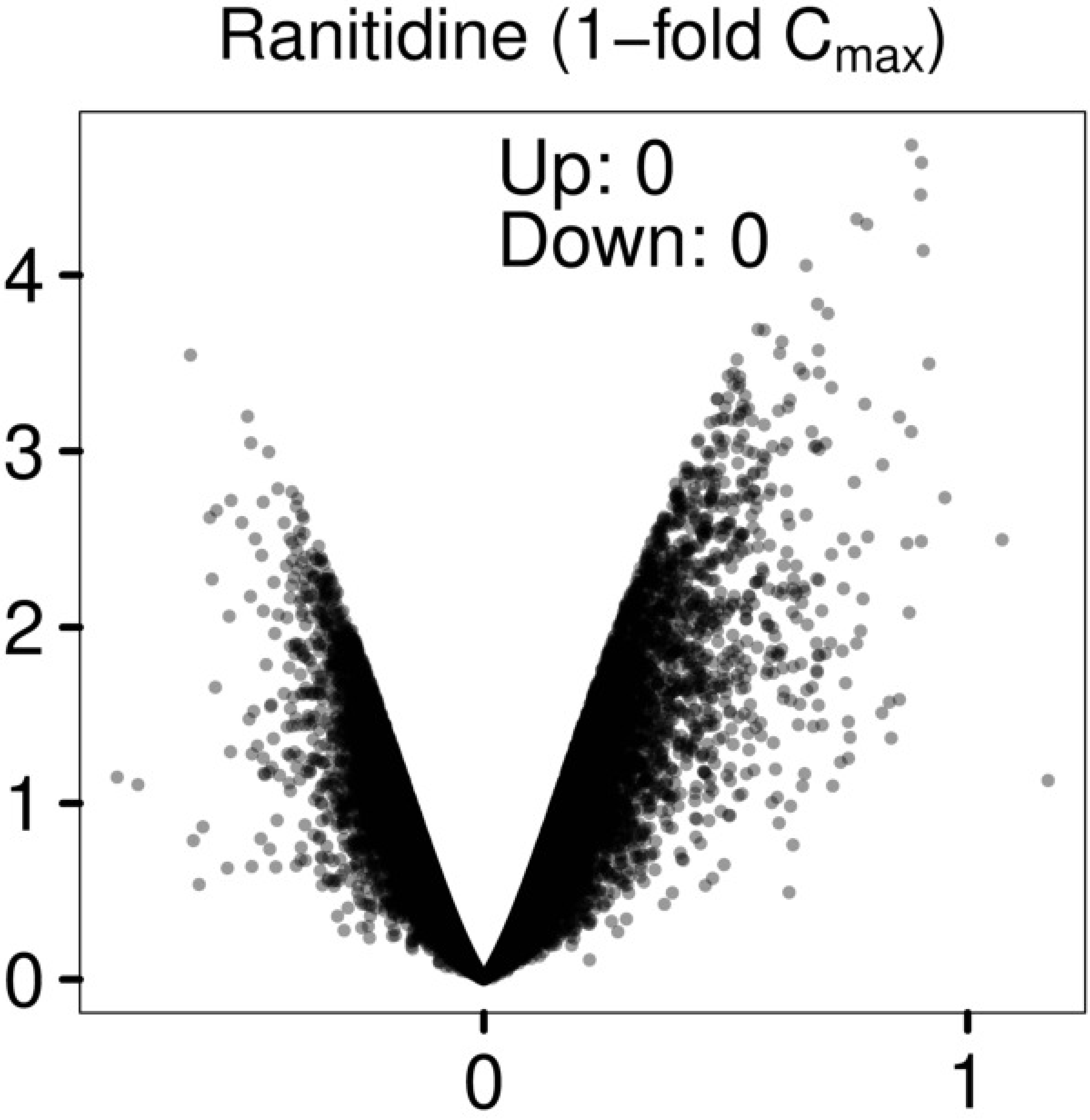

Ranitidine (1–fold Cmax)
Up: 0
Down: 0
4
3
2
1
0
0
1

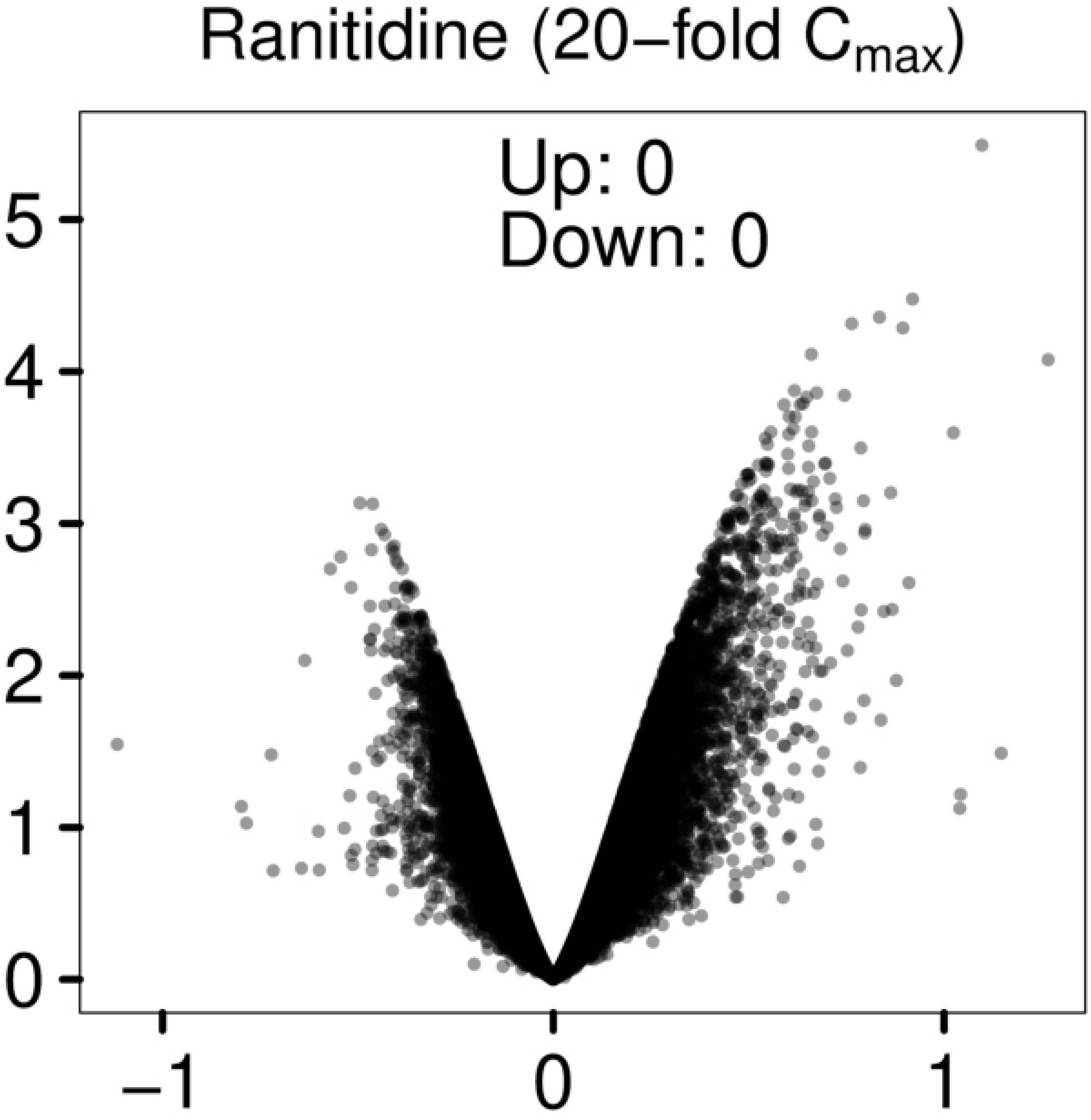
Ranitidine (20−fold C_{max})
Up: 0
Down: 0
5
4
3
2
1
0
−1
0
1

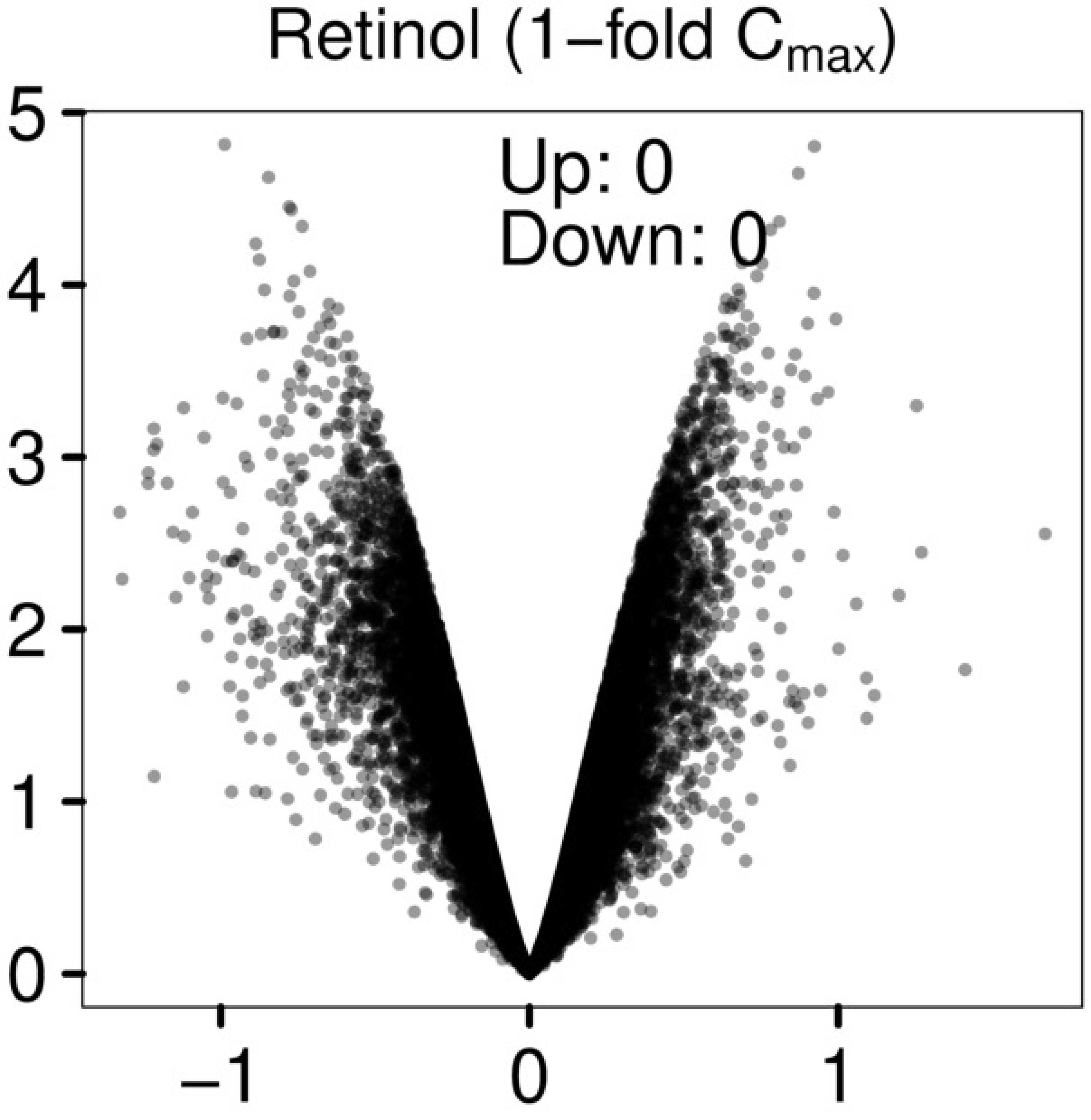
Retinol (1–fold C_{max})
Up: 0
Down: 0
5
4
3
2
1
0
–1
0
1

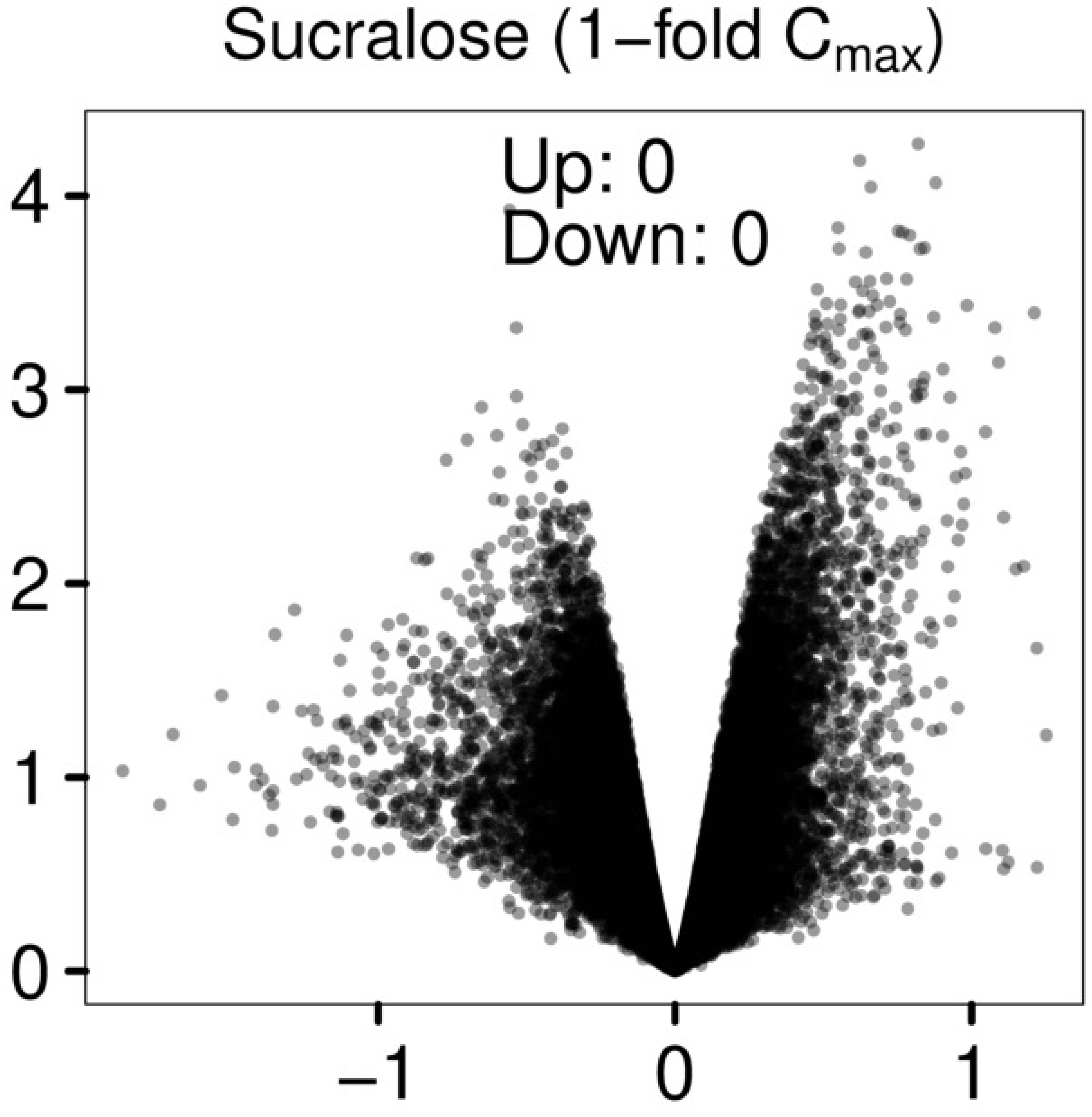
Sucralose (1–fold C max)
Up: 0
Down: 0
4
3
2
1
0
–1
0
1

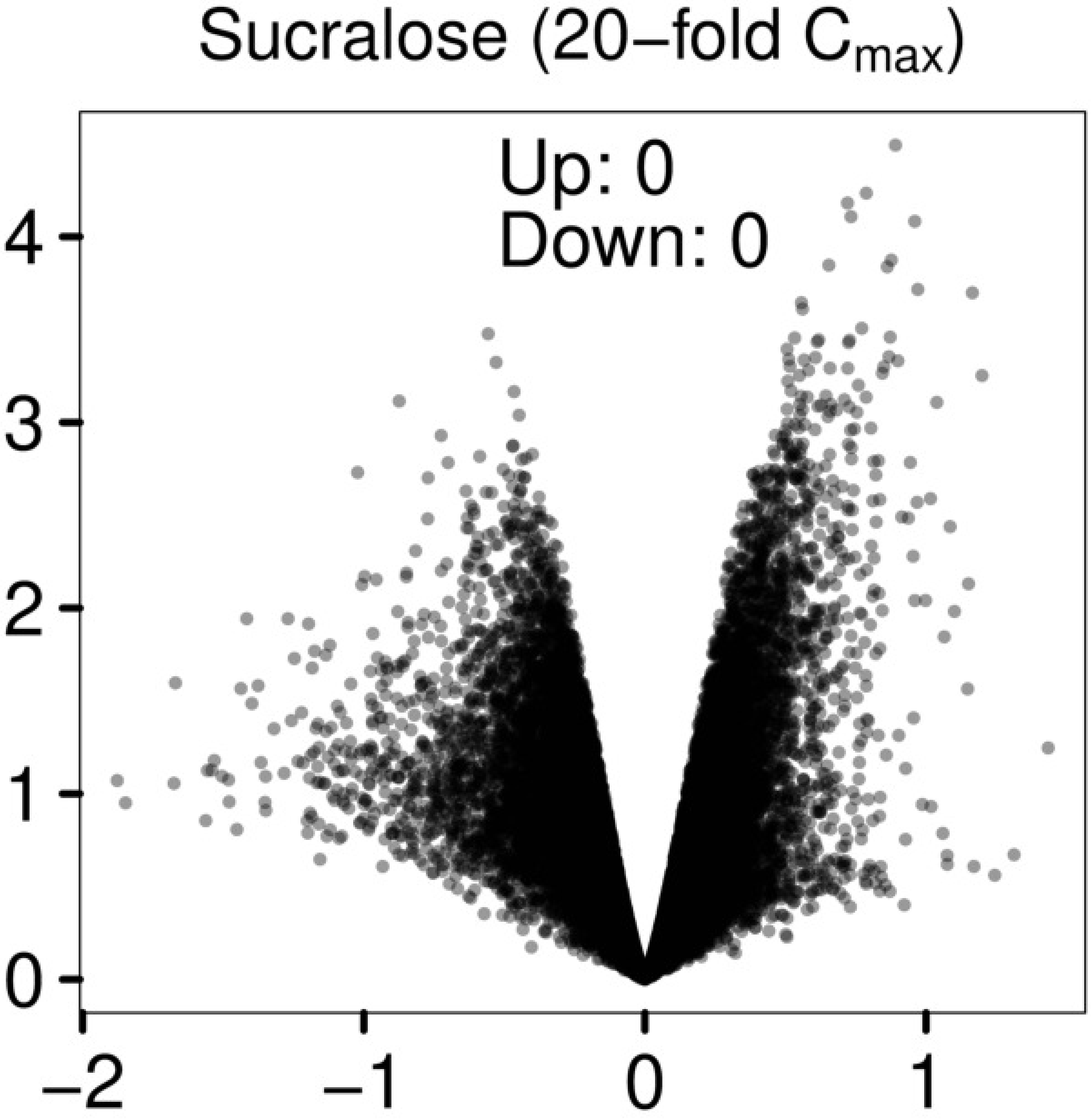
Sucralose (20–fold C_{max})
Up: 0
Down: 0
4
3
2
1
0
−2
−1
0
1

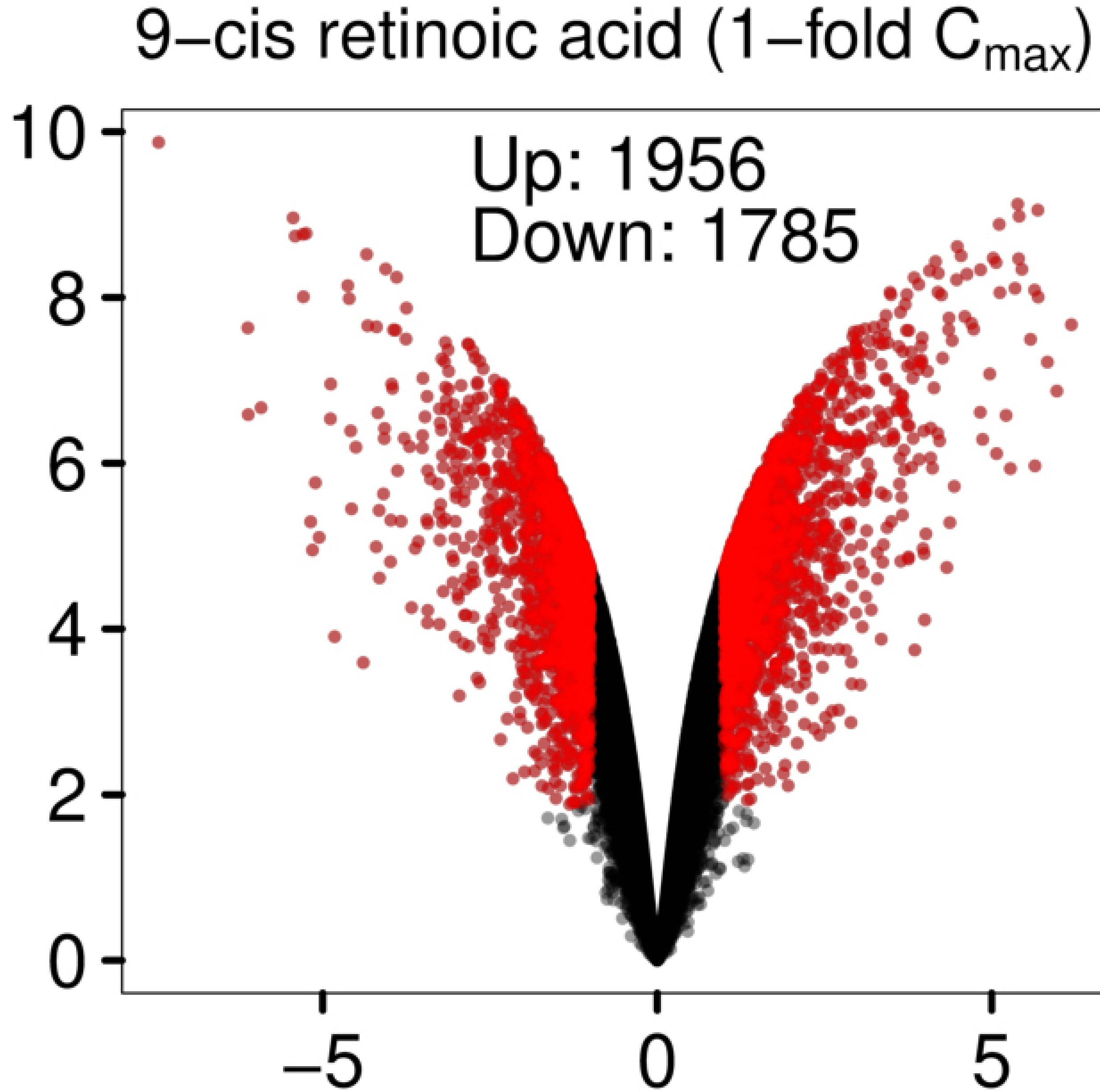

9−cis retinoic acid (1−fold C_{max})
Up: 1956
Down: 1785
10
8
6
4
2
0
−5
0
5

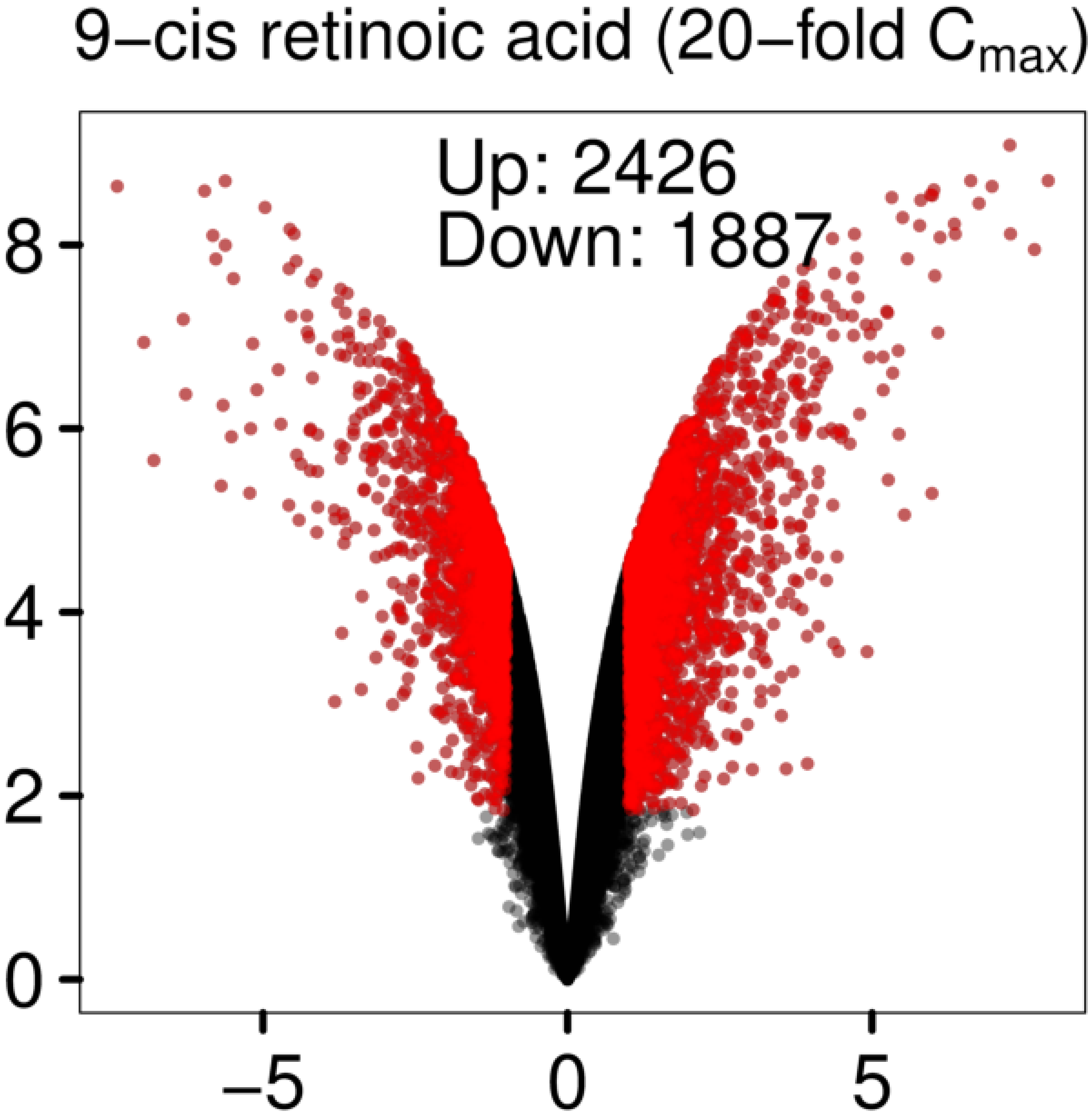

9−cis retinoic acid (20−fold Cmax)
Up: 2426
Down: 1887
8
6
4
2
0
−5
0
5

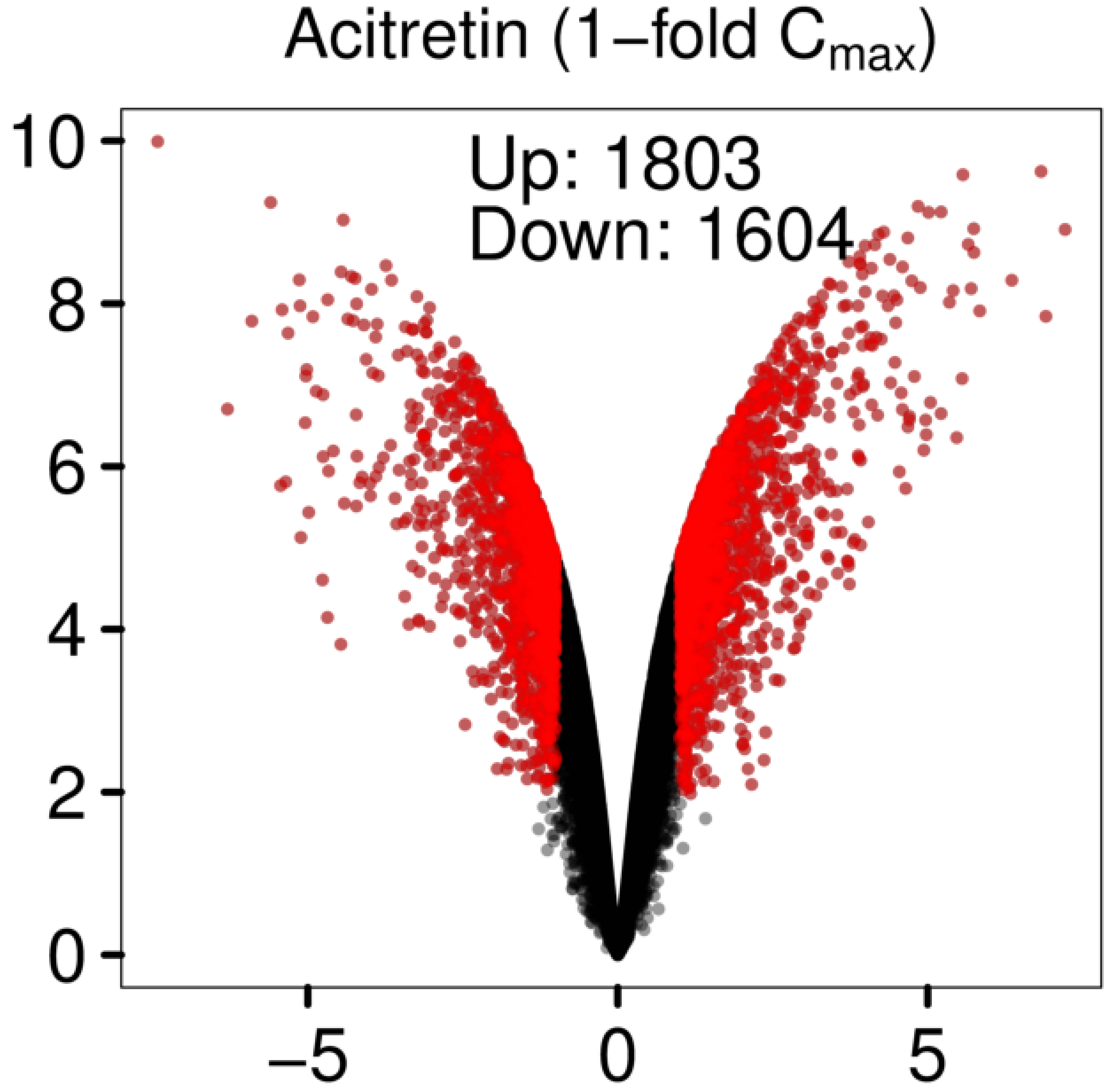
Acitretin (1–fold C_{max})
Up: 1803
Down: 1604
10
8
6
4
2
0
–5
0
5

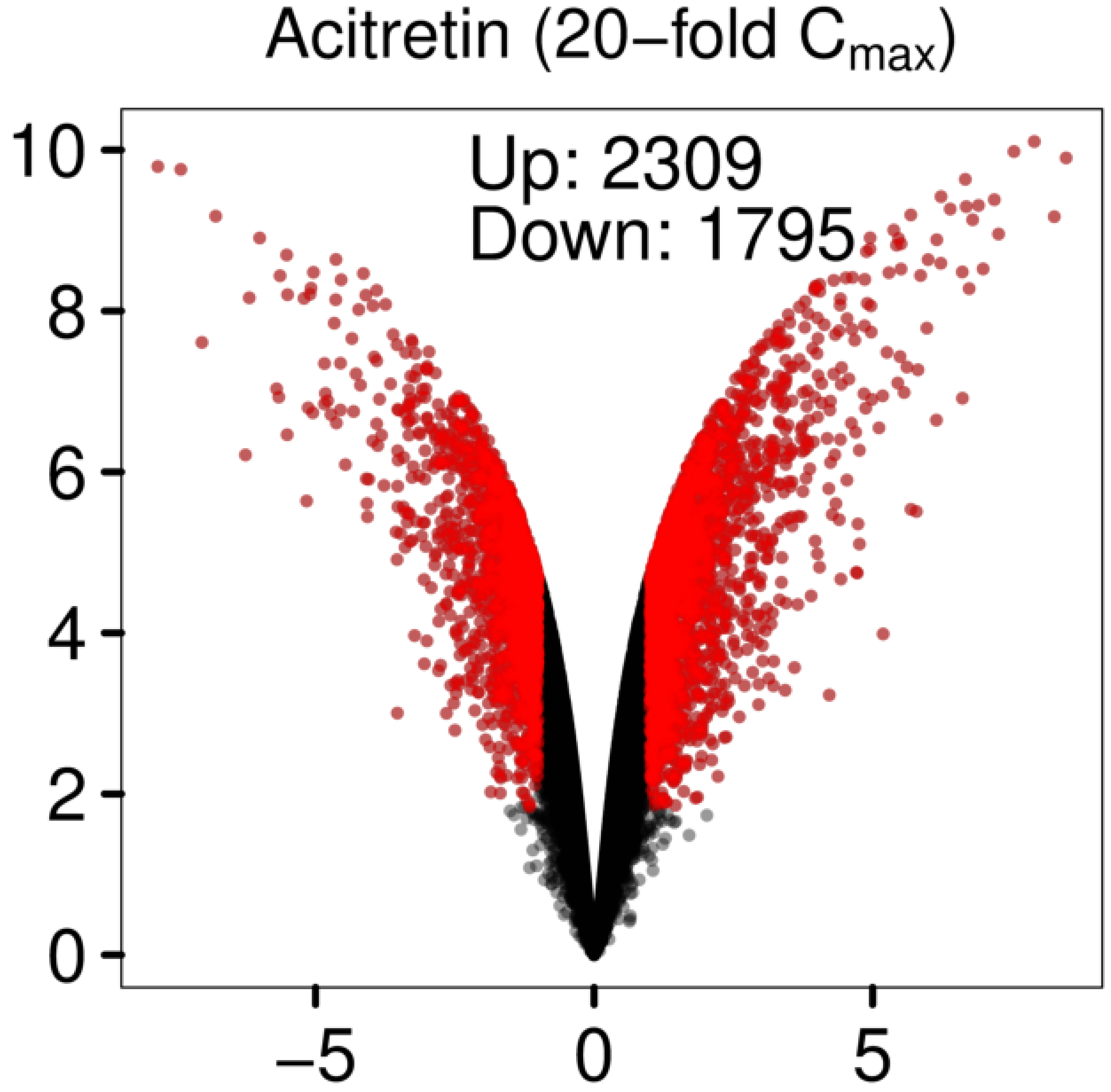

Acitretin (20−fold C_{max})
Up: 2309
Down: 1795
10
8
6
4
2
0
−5
0
5

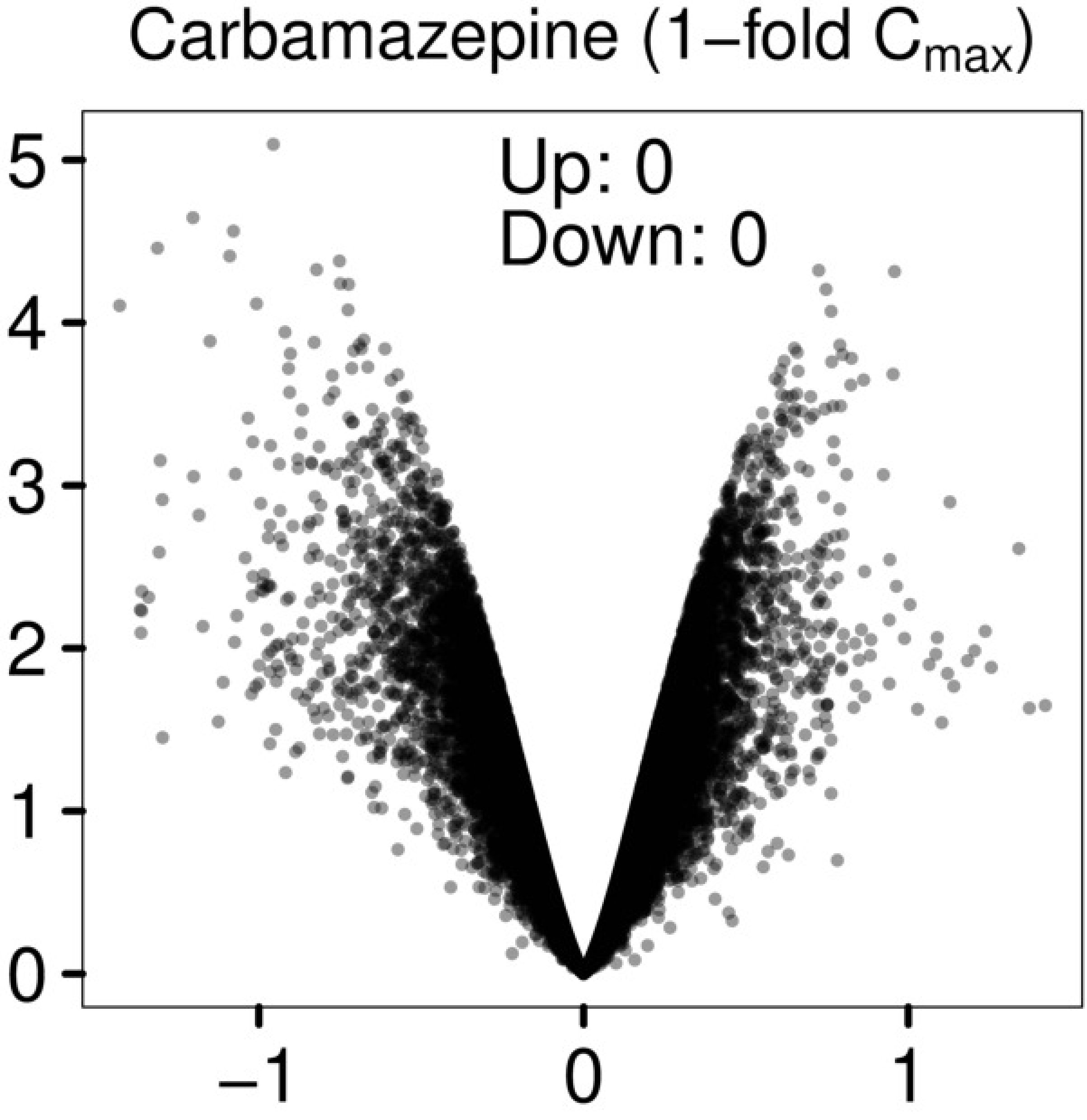

Carbamazepine (1−fold C max)
Up: 0
Down: 0
5
4
3
2
1
0
−1
0
1

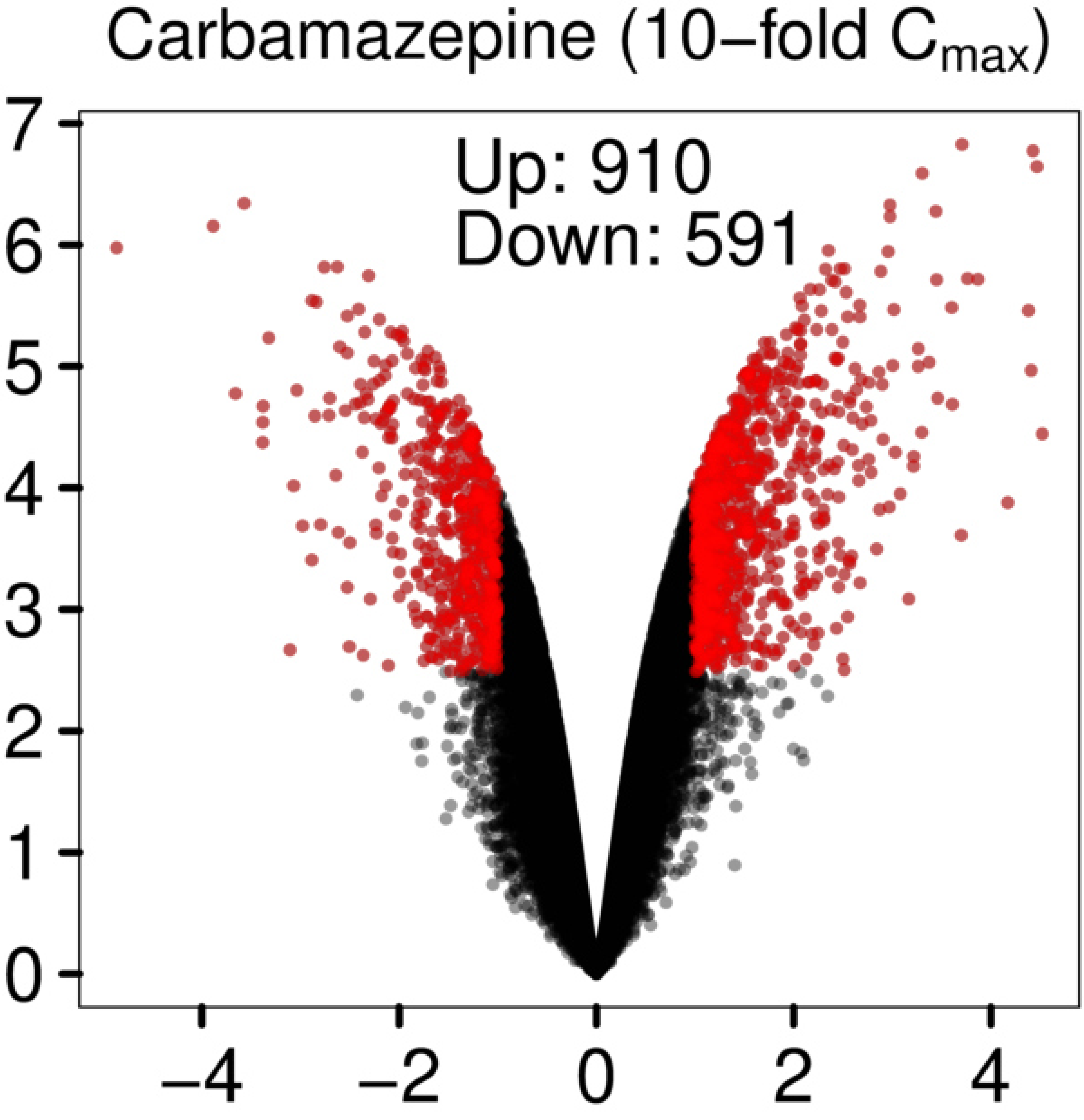

Carbamazepine (10−fold C_{max})
Up: 910
Down: 591
7
6
5
4
3
2
1
0
−4
−2
0
2
4

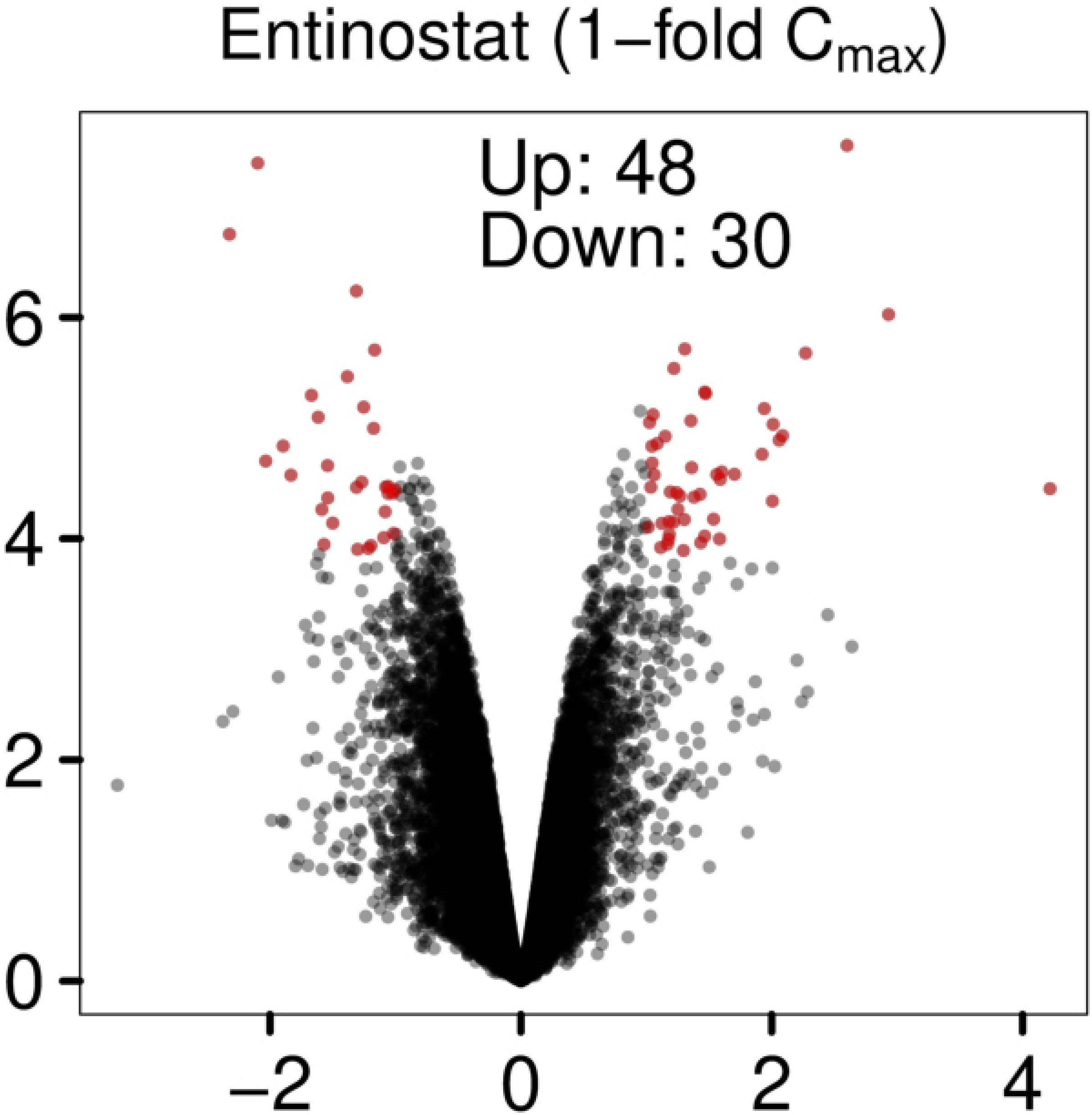

Entinostat (1−fold C$_{max}$)
Up: 48
Down: 30
6
4
2
0
−2
0
2
4

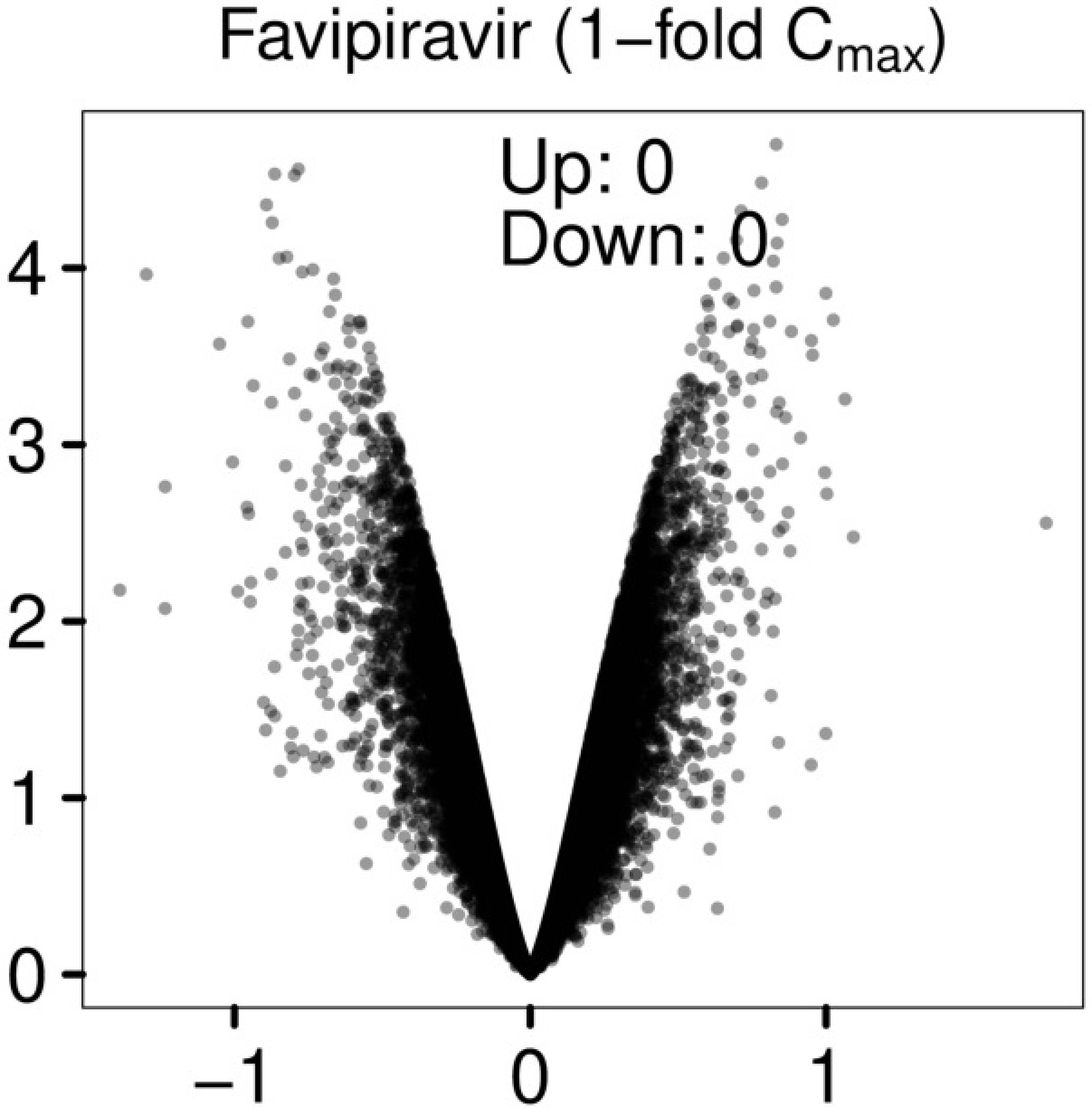

Favipiravir (1–fold C_{max})
Up: 0
Down: 0
4
3
2
1
0
–1
0
1

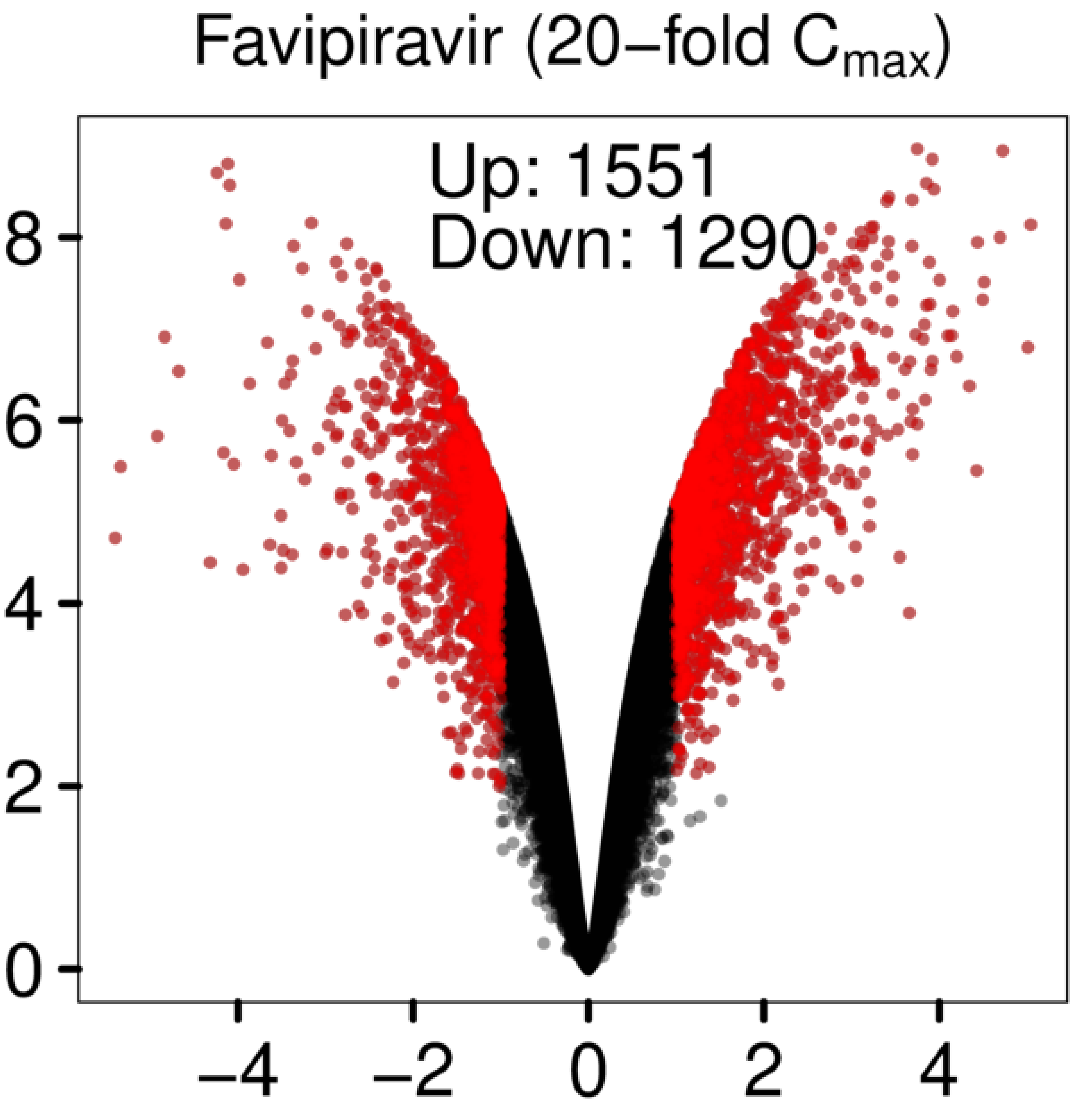

Favipiravir (20–fold C max)
Up: 1551
Down: 1290
8
6
4
2
0
–4
–2
0
2
4

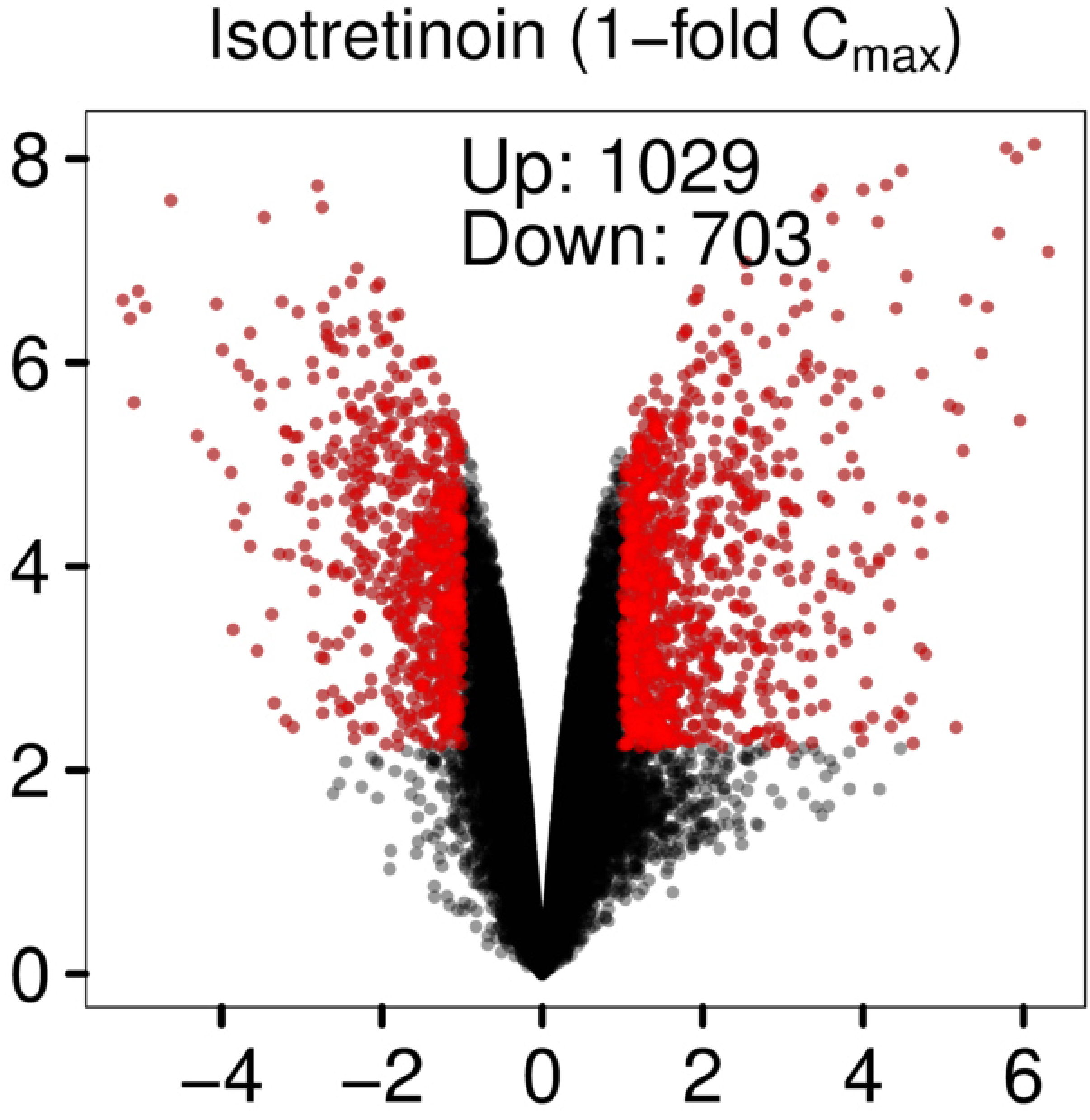
Isotretinoin (1–fold C max)
Up: 1029
Down: 703
8
6
4
2
0
–4
–2
0
2
4
6

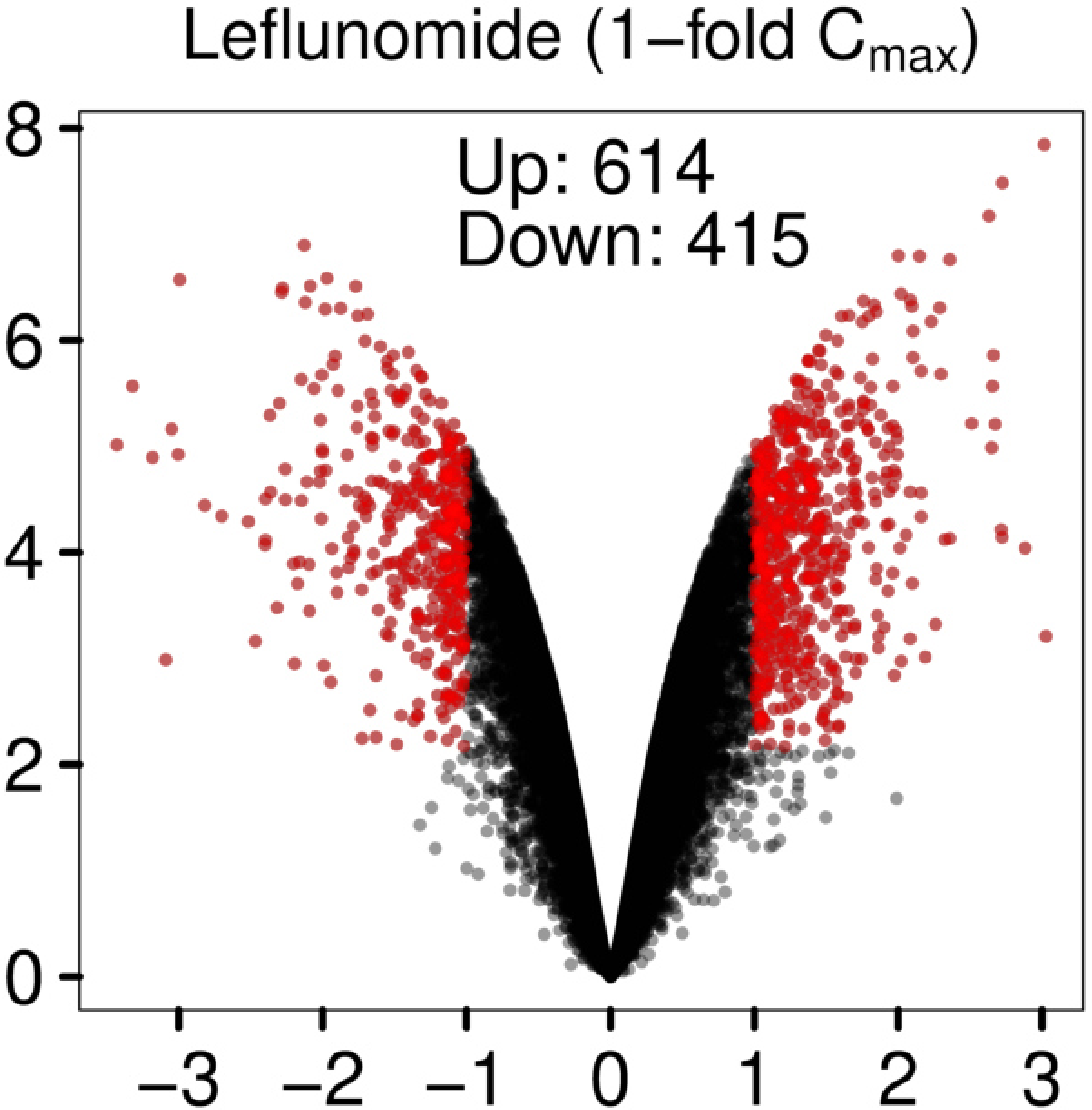
Leflunomide (1−fold C_{max})
Up: 614
Down: 415
8
6
4
2
0
−3
−2
−1
0
1
2
3

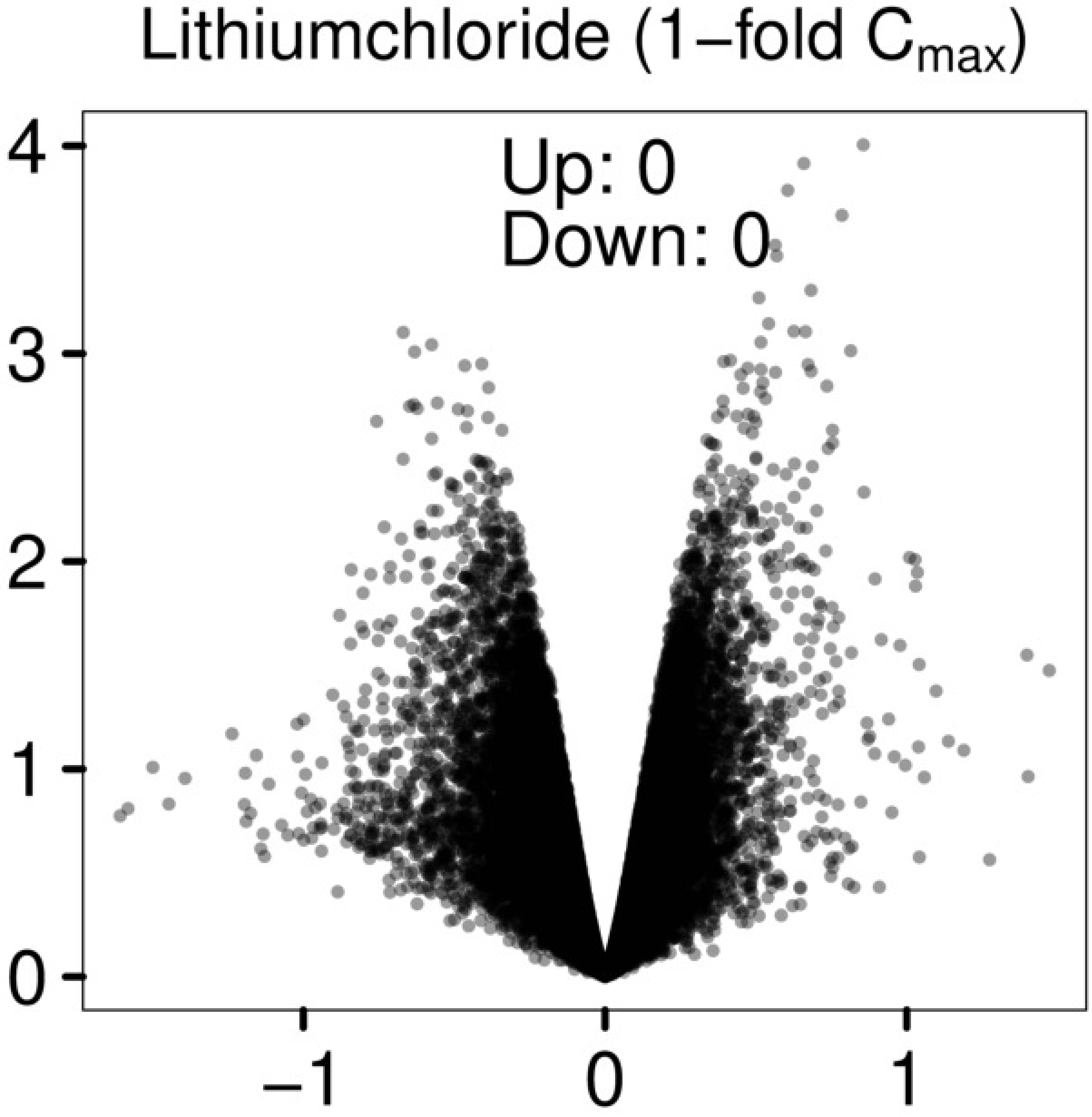

Lithiumchloride (1−fold C_{max})
Up: 0
Down: 0
4
3
2
1
0
−1
0
1

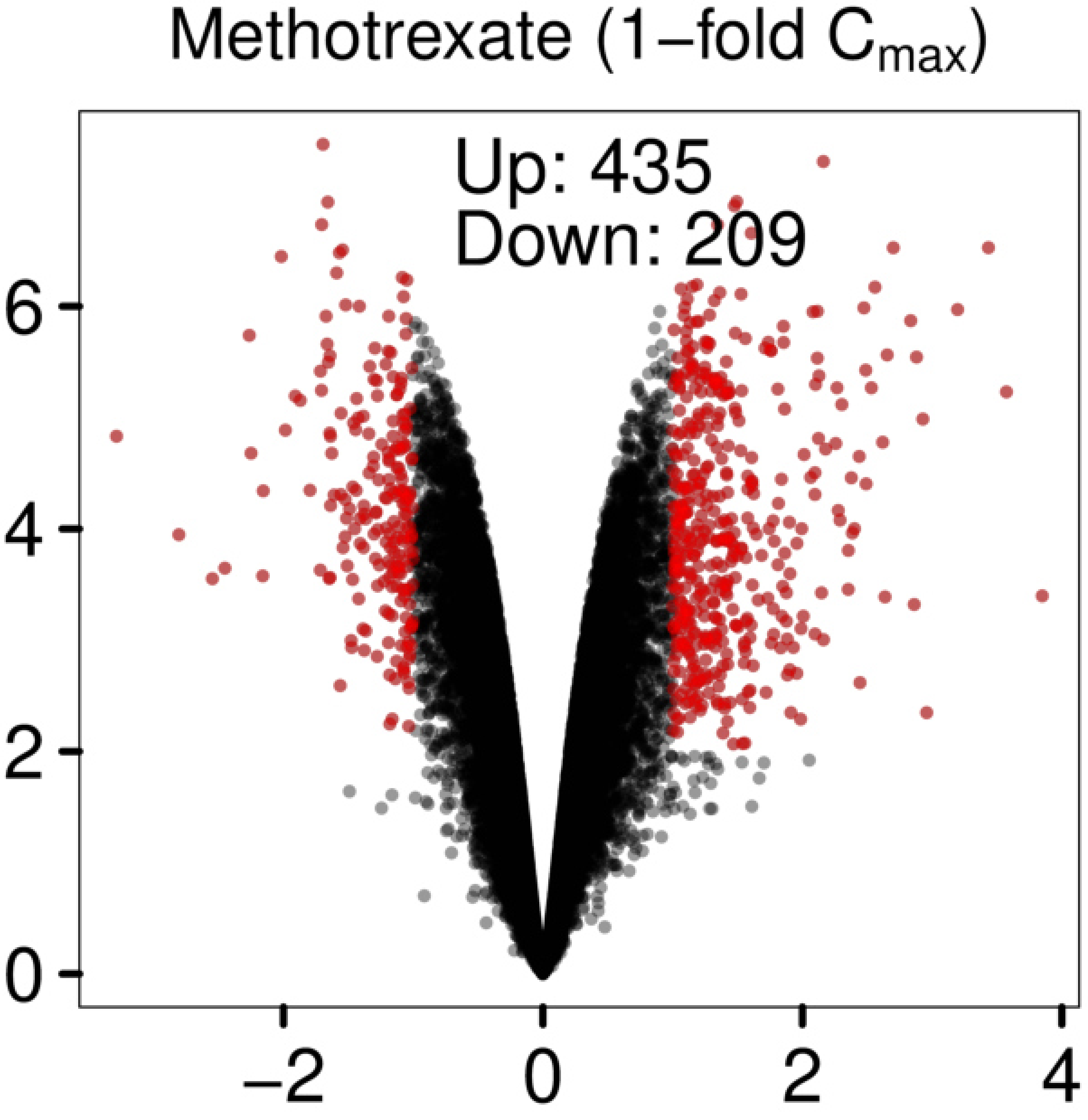
Methotrexate (1–fold C max)
Up: 435
Down: 209
6
4
2
0
−2
0
2
4

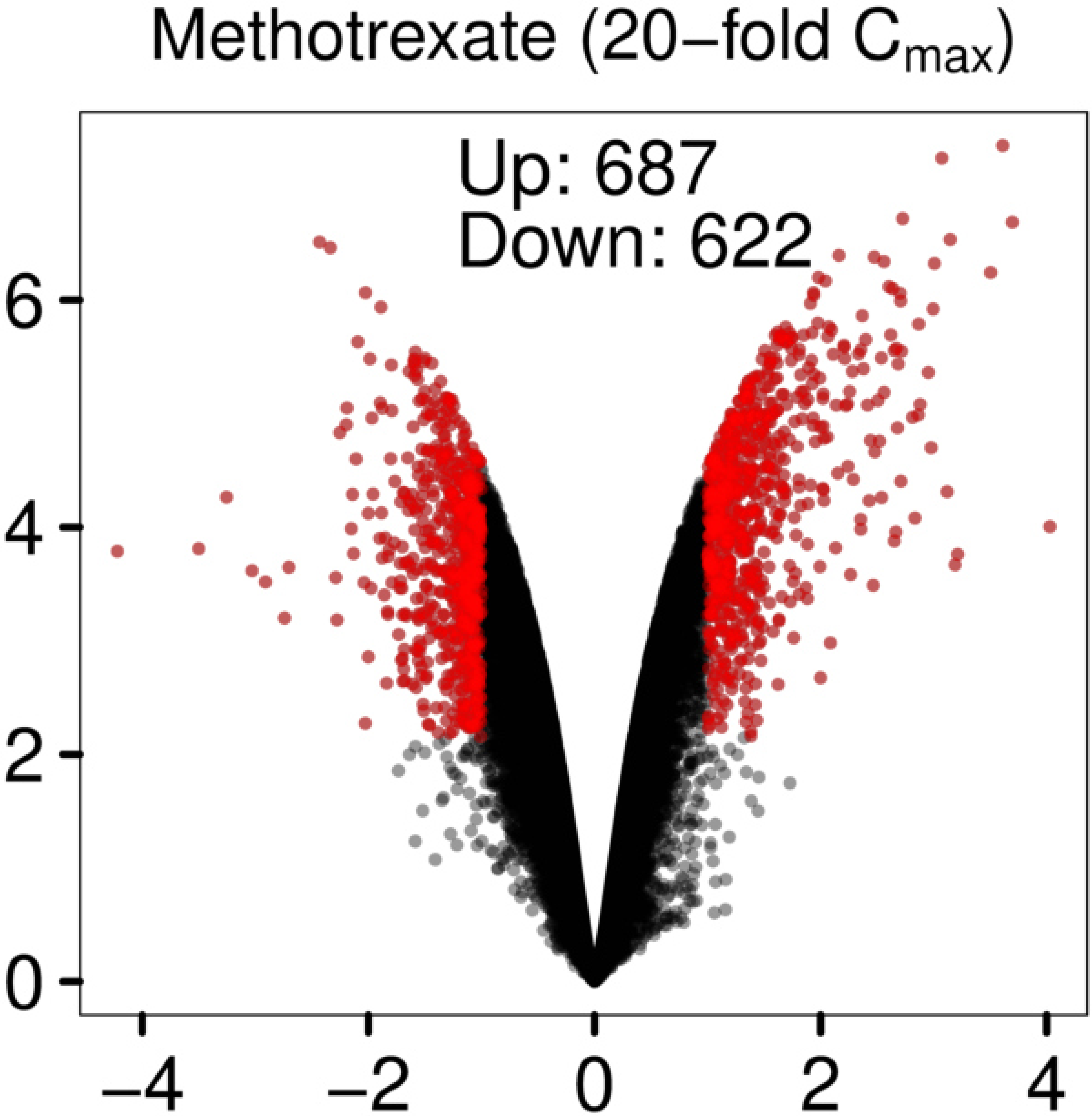

Methotrexate (20−fold C$_{max}$)
Up: 687
Down: 622
6
4
2
0
−4
−2
0
2
4

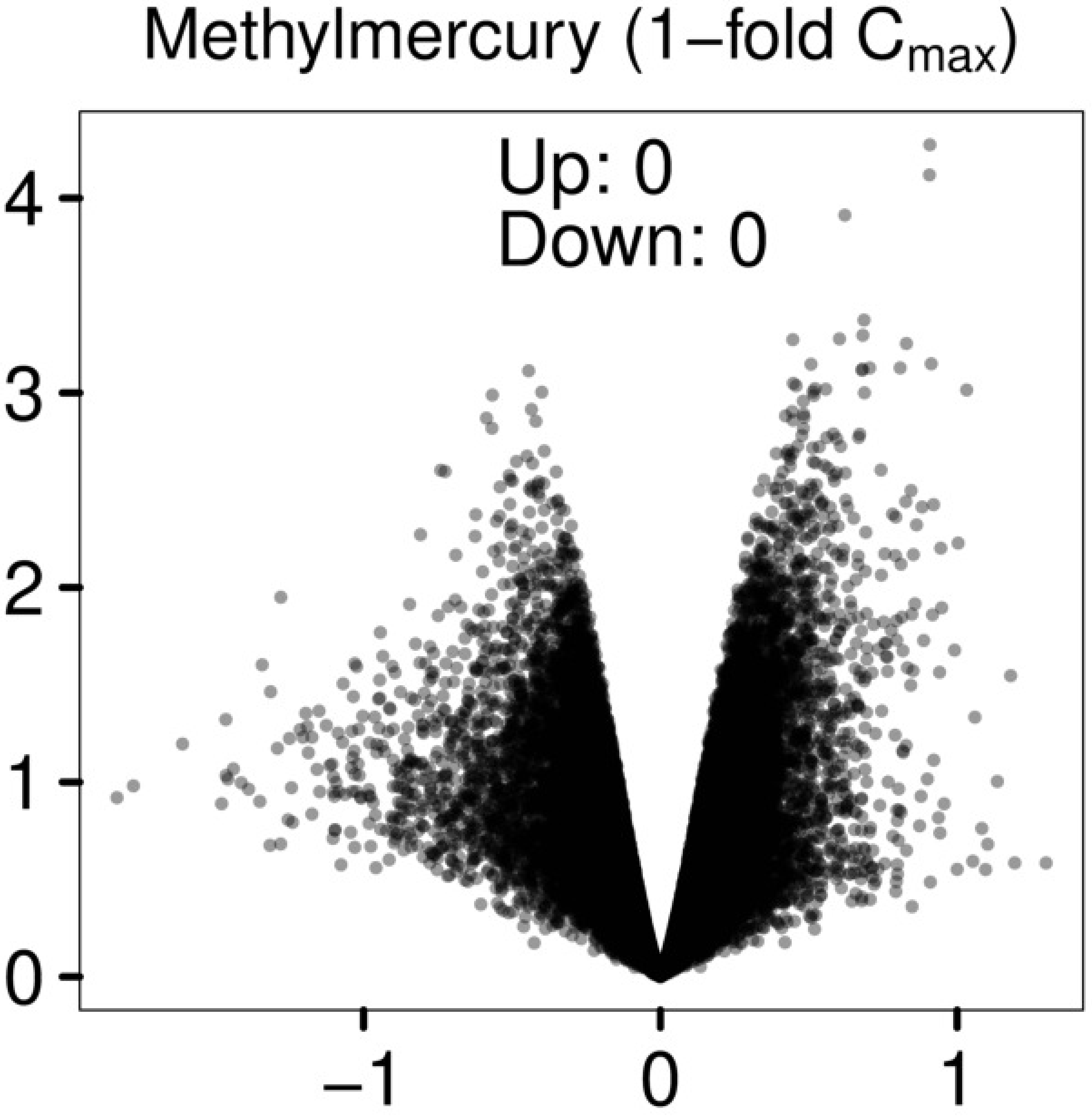
Methylmercury (1–fold C_{max})
Up: 0
Down: 0
4
3
2
1
0
–1
0
1

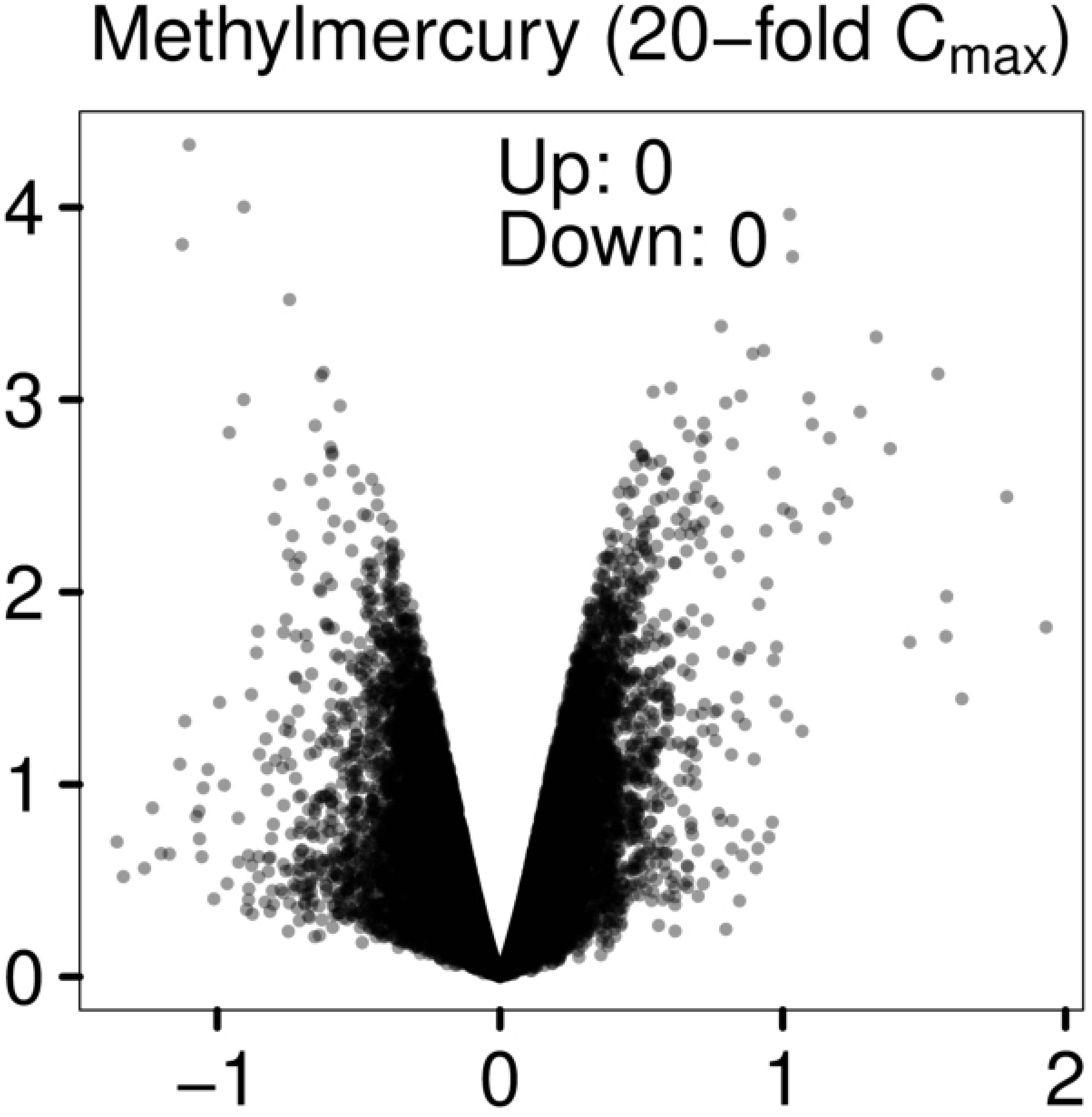
Methylmercury (20–fold C_{max})
Up: 0
Down: 0
4
3
2
1
0
–1
0
1
2

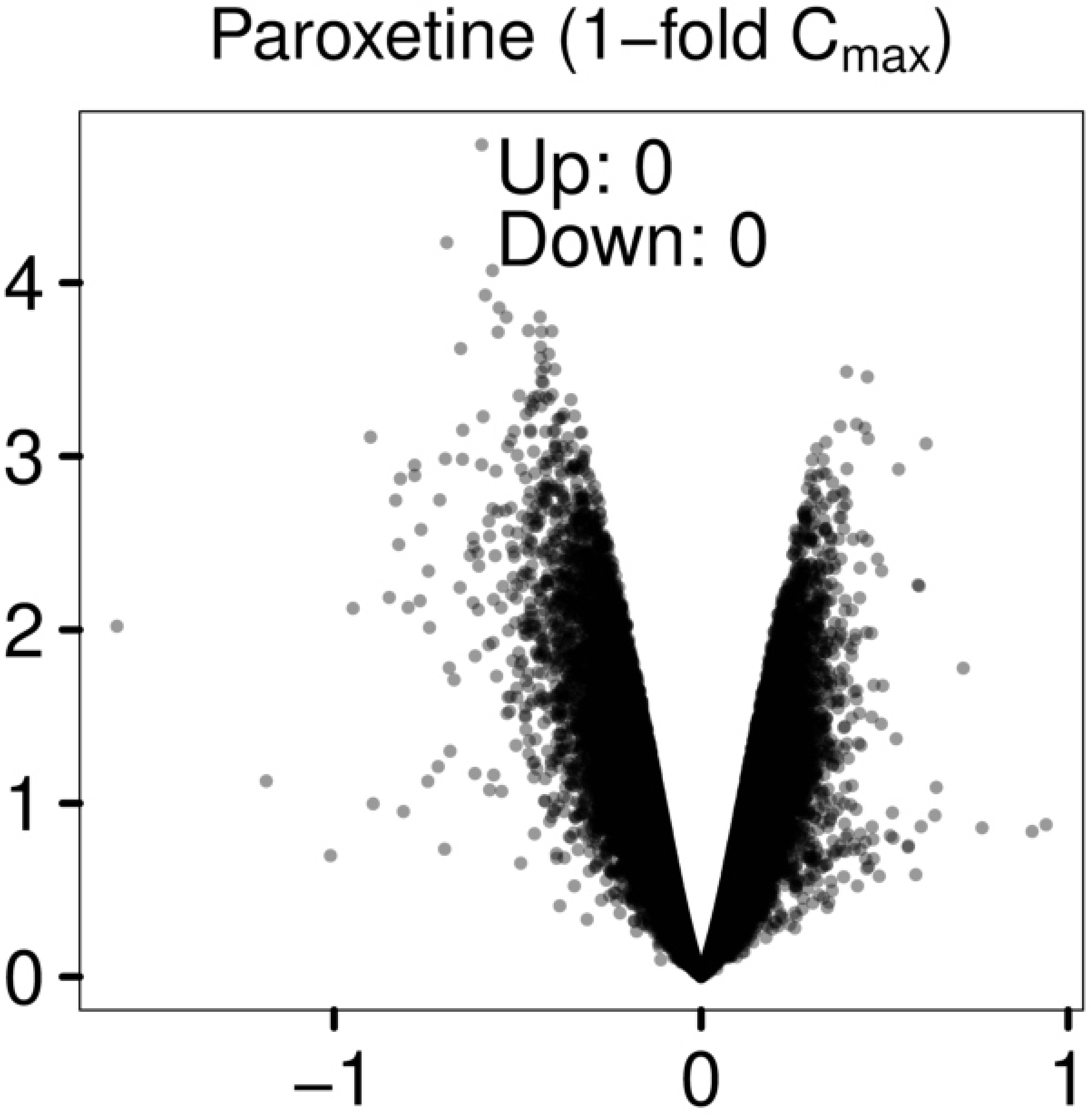
Paroxetine (1−fold C_{max})
Up: 0
Down: 0
4
3
2
1
0
−1
0
1

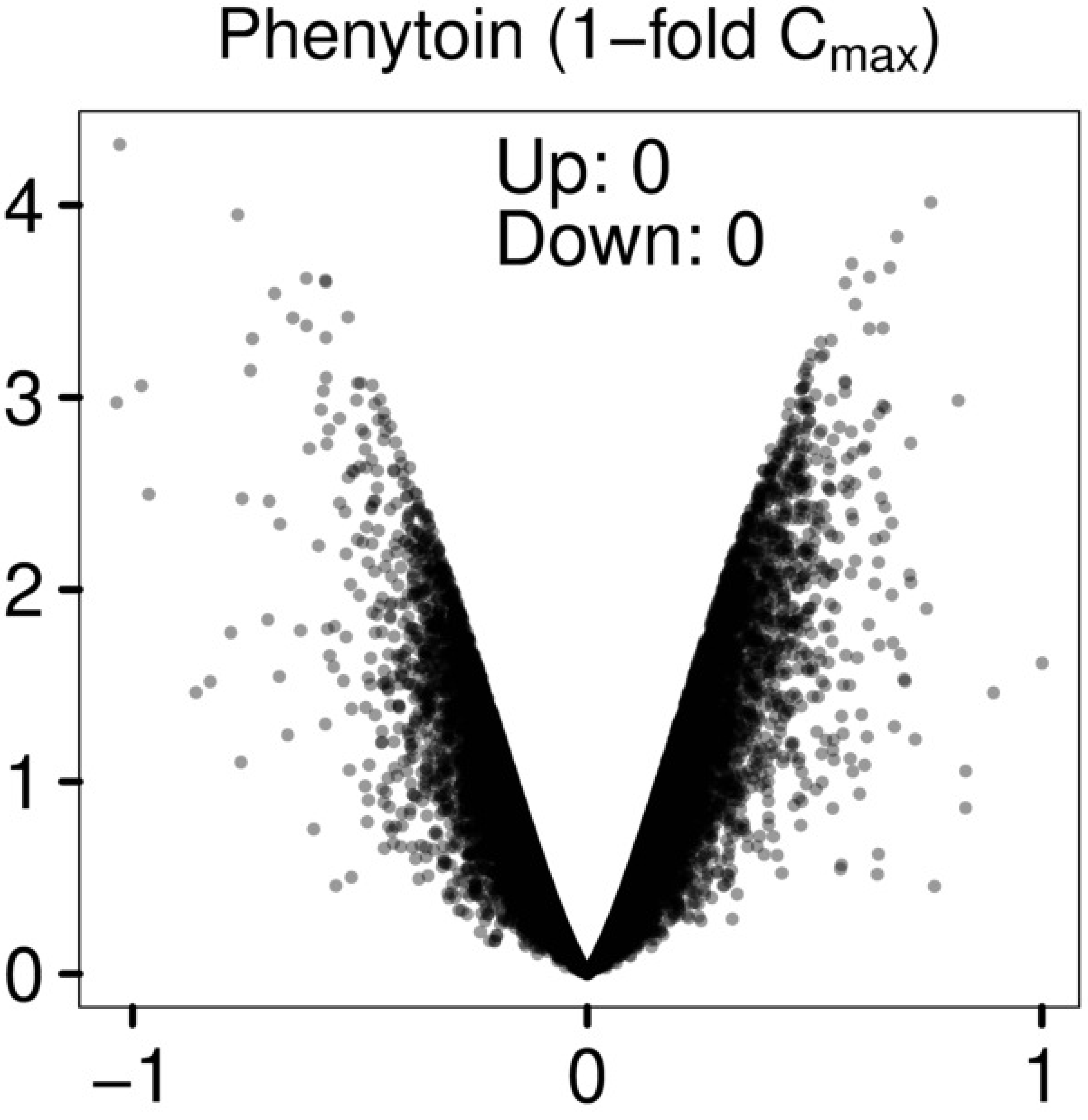
Phenytoin (1–fold Cmax)
Up: 0
Down: 0
4
3
2
1
0
–1
0
1

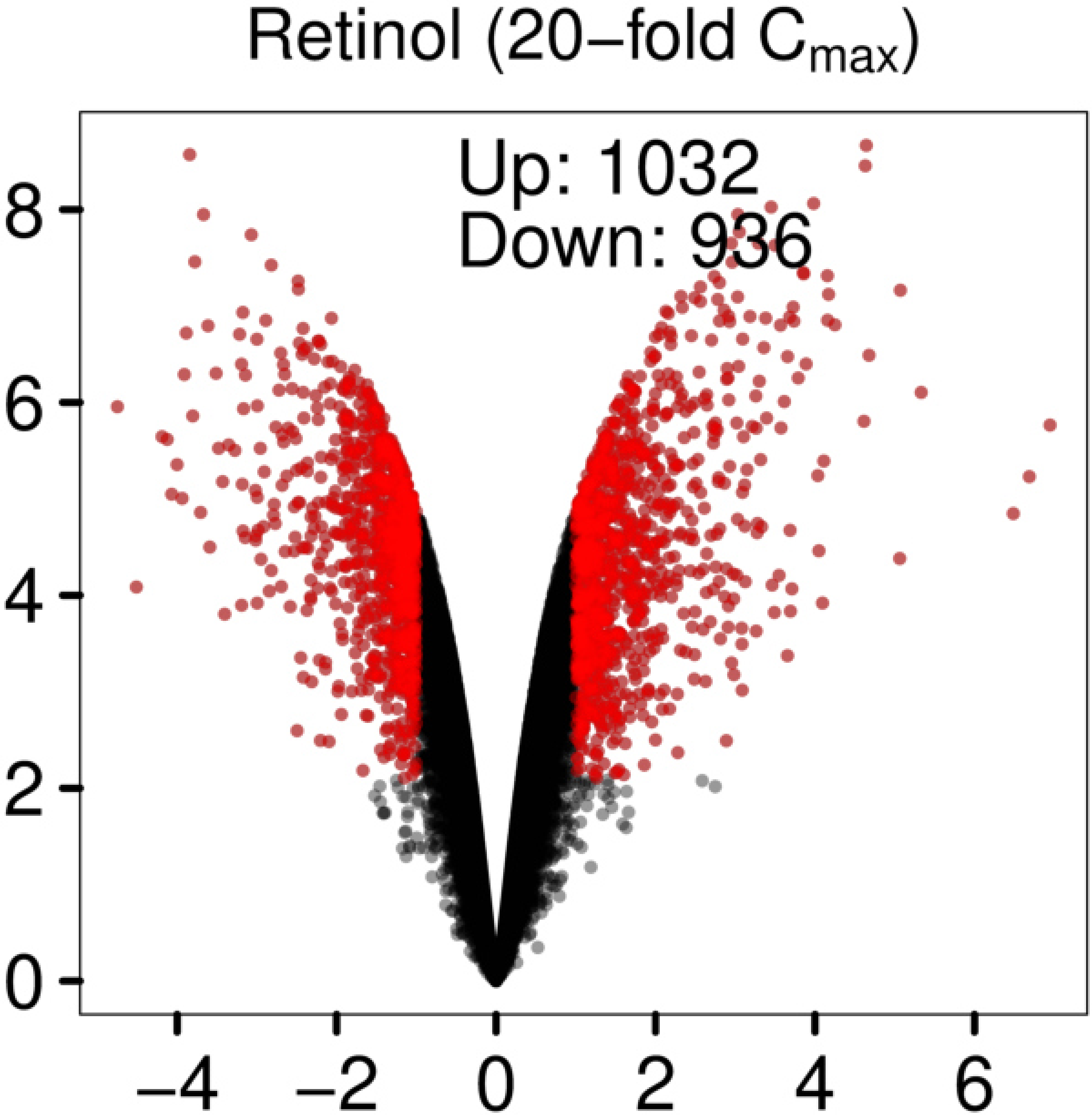

Retinol (20−fold C_{max})
Up: 1032
Down: 936
8
6
4
2
0
−4
−2
0
2
4
6

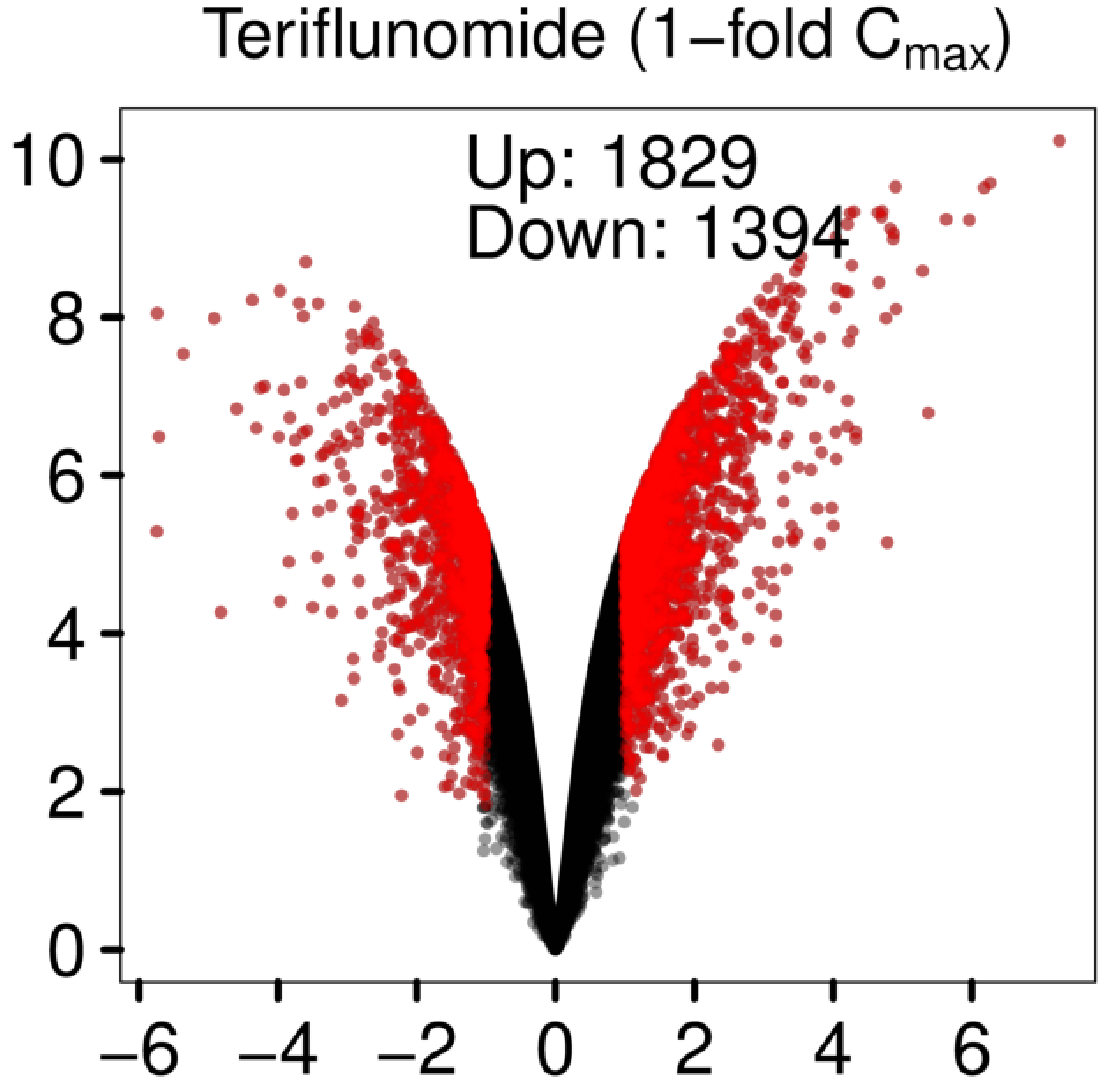
Teriflunomide (1−fold C$_{max}$)
Up: 1829
Down: 1394
10
8
6
4
2
0
−6
−4
−2
0
2
4
6

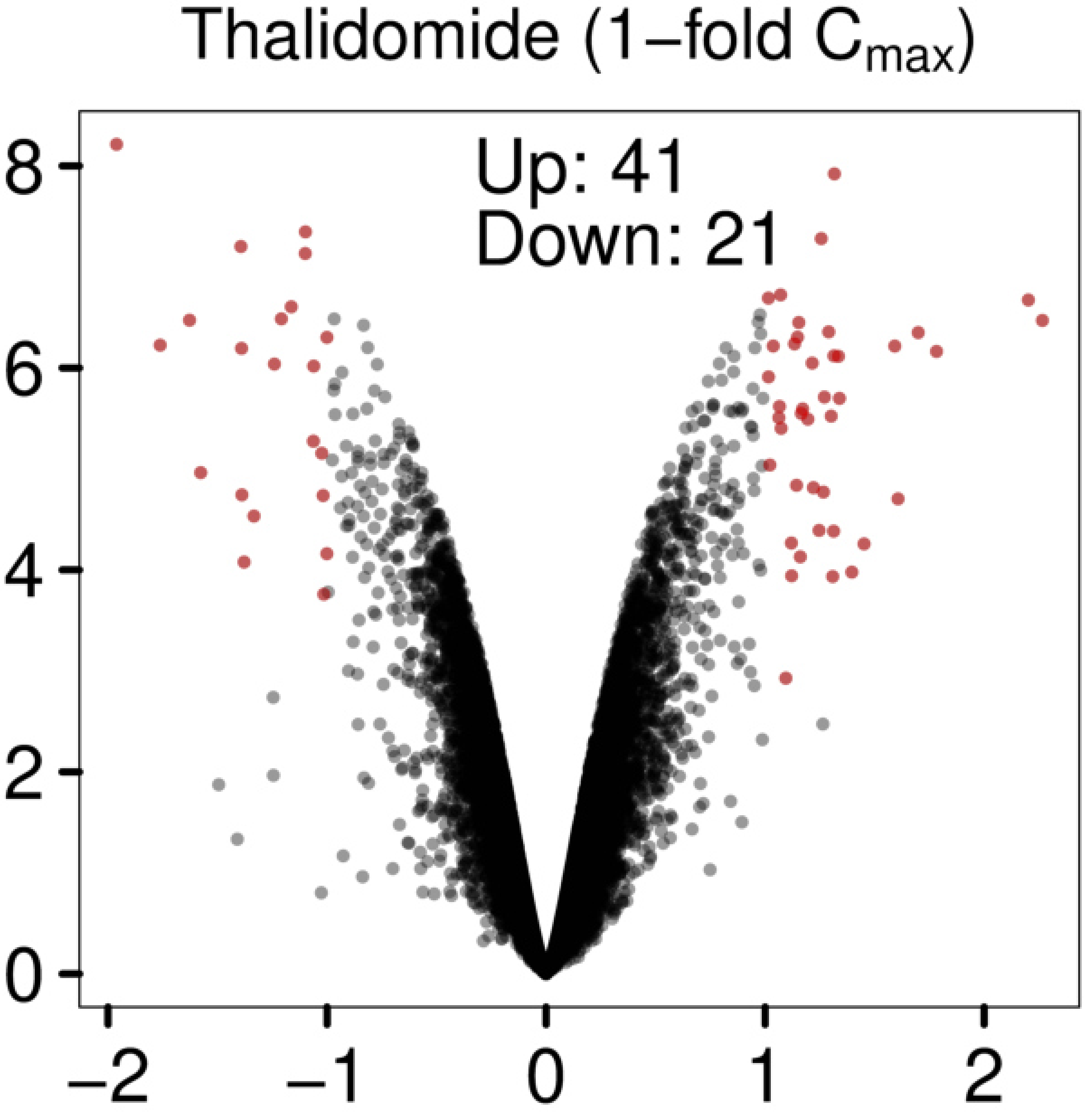
Thalidomide (1−fold C_{max})
Up: 41
Down: 21
8
6
4
2
0
−2
−1
0
1
2

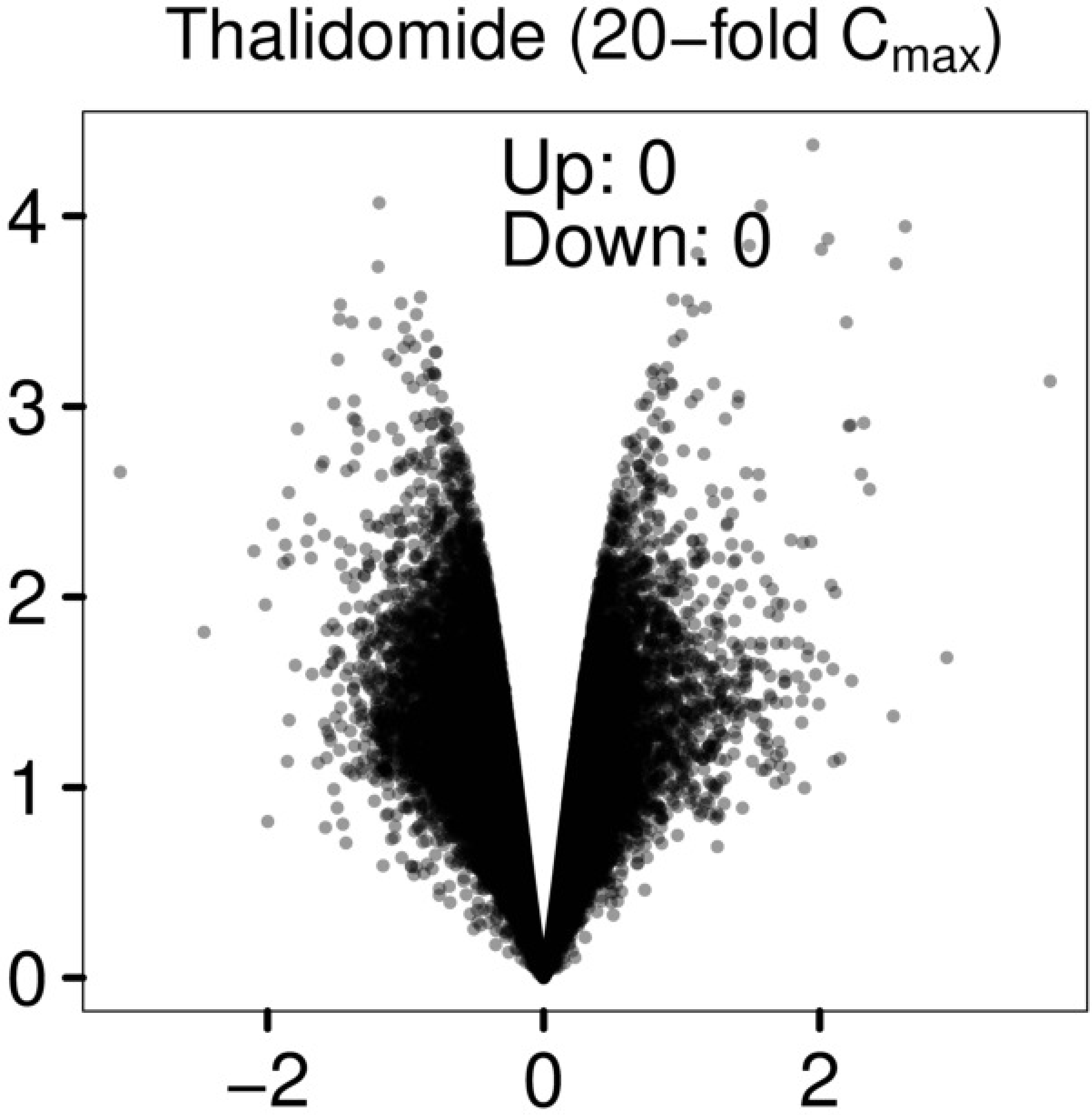
Thalidomide (20–fold C$_{max}$)
Up: 0
Down: 0
4
3
2
1
0
–2
0
2

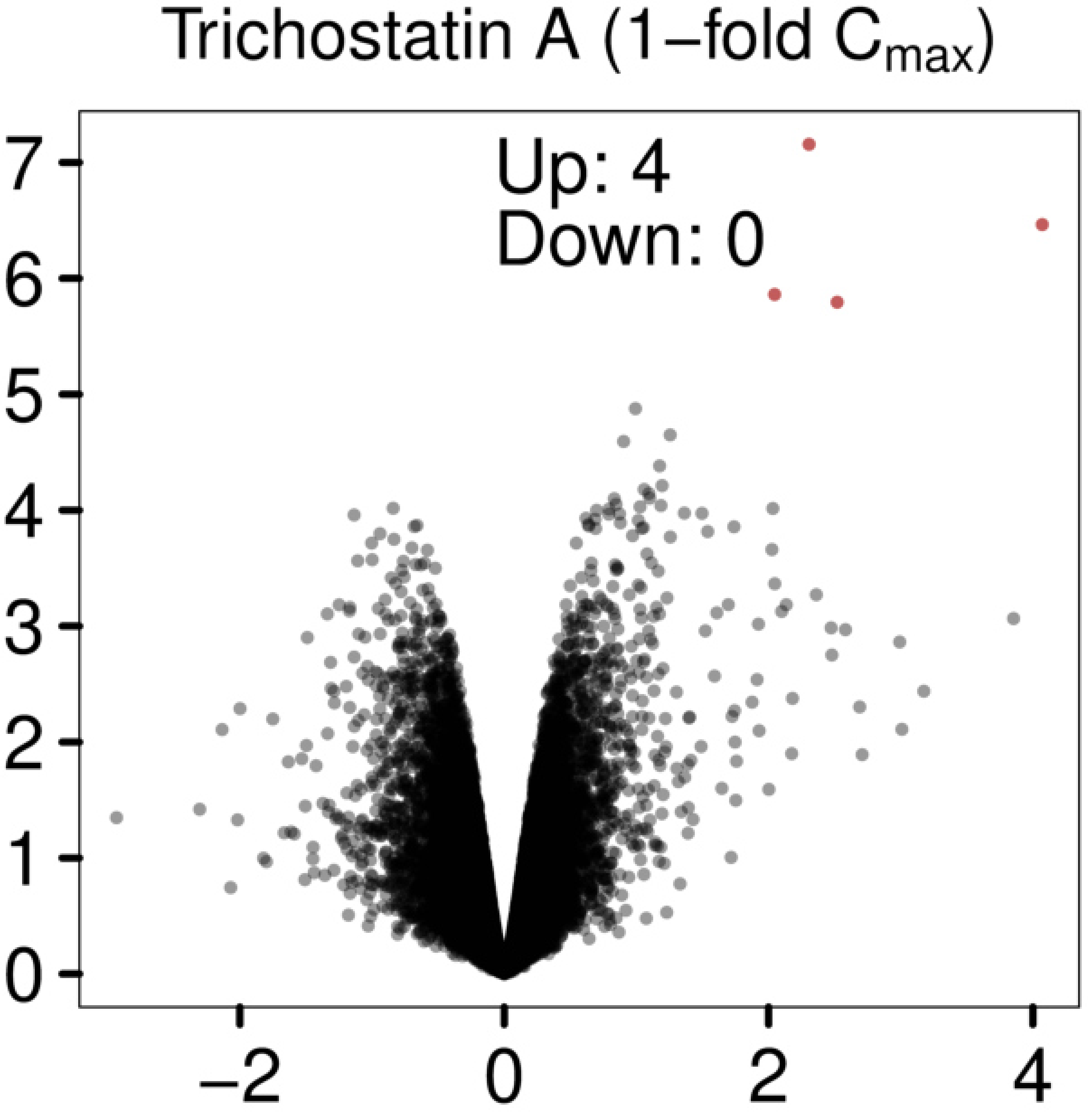

Trichostatin A (1–fold C max)
Up: 4
Down: 0
7
6
5
4
3
2
1
0
−2
0
2
4

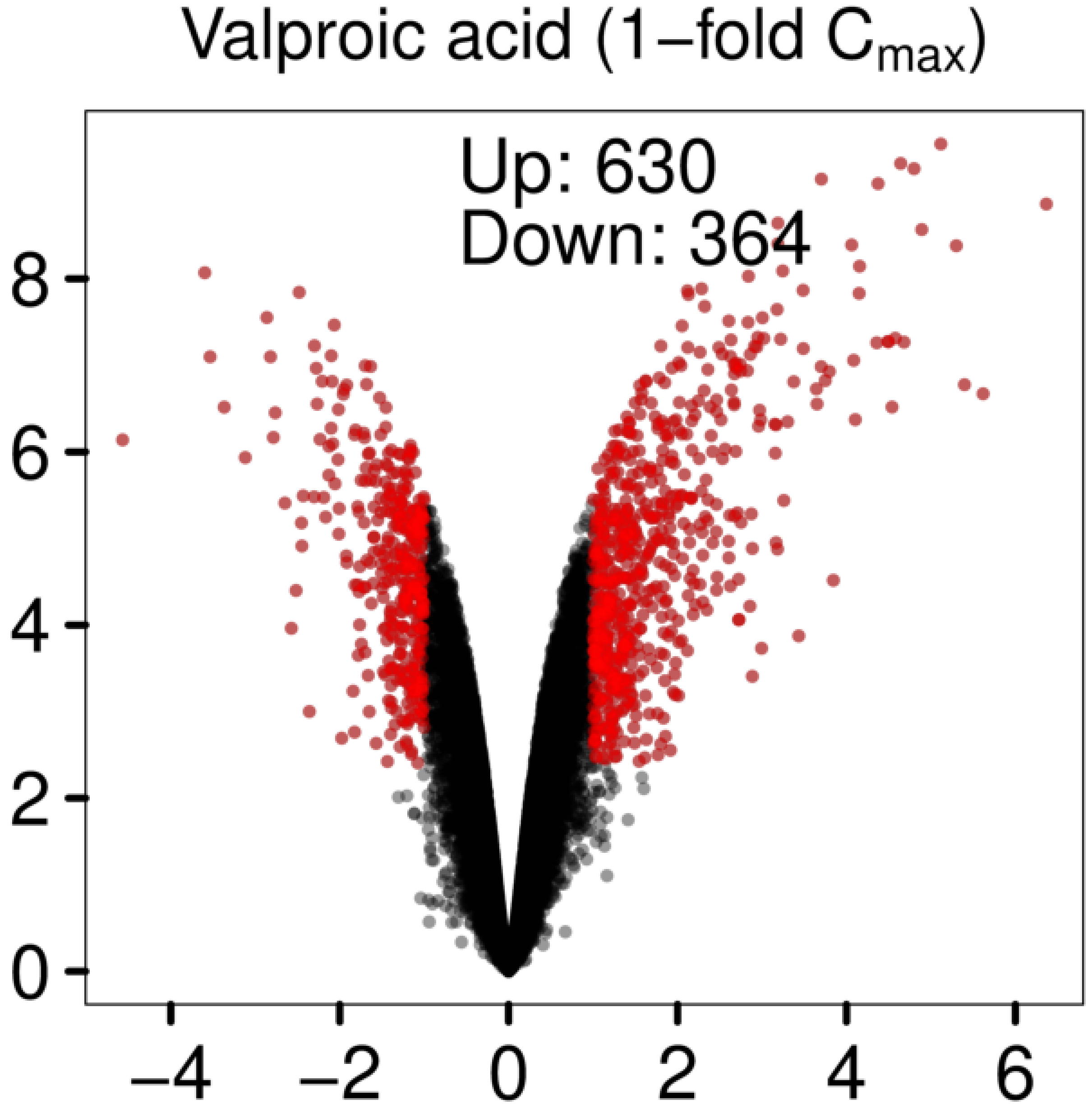
Valproic acid (1–fold C max)
Up: 630
Down: 364
8
6
4
2
0
–4
–2
0
2
4
6

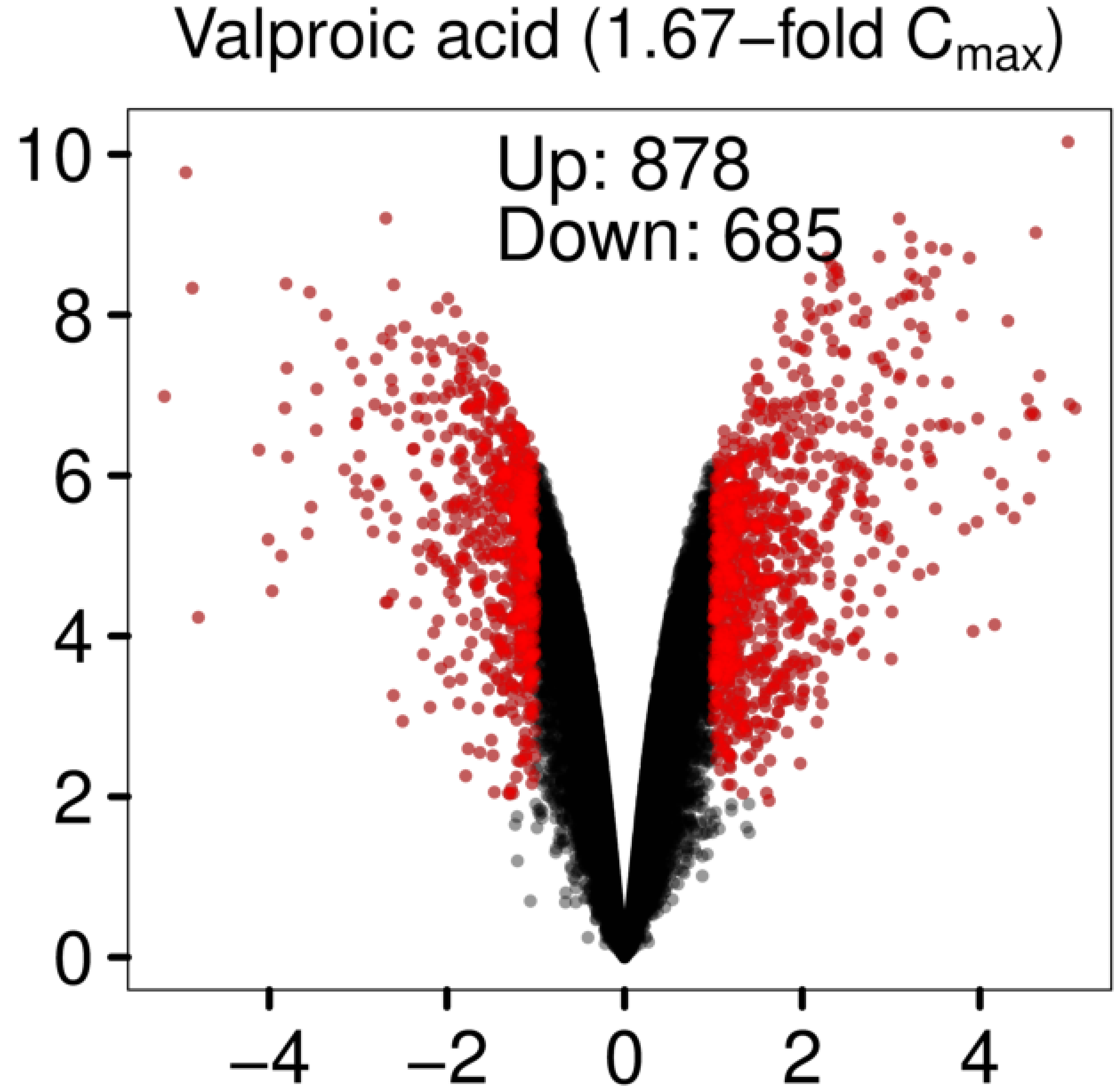
Valproic acid (1.67–fold C_max)
Up: 878
Down: 685
10
8
6
4
2
0
–4
–2
0
2
4

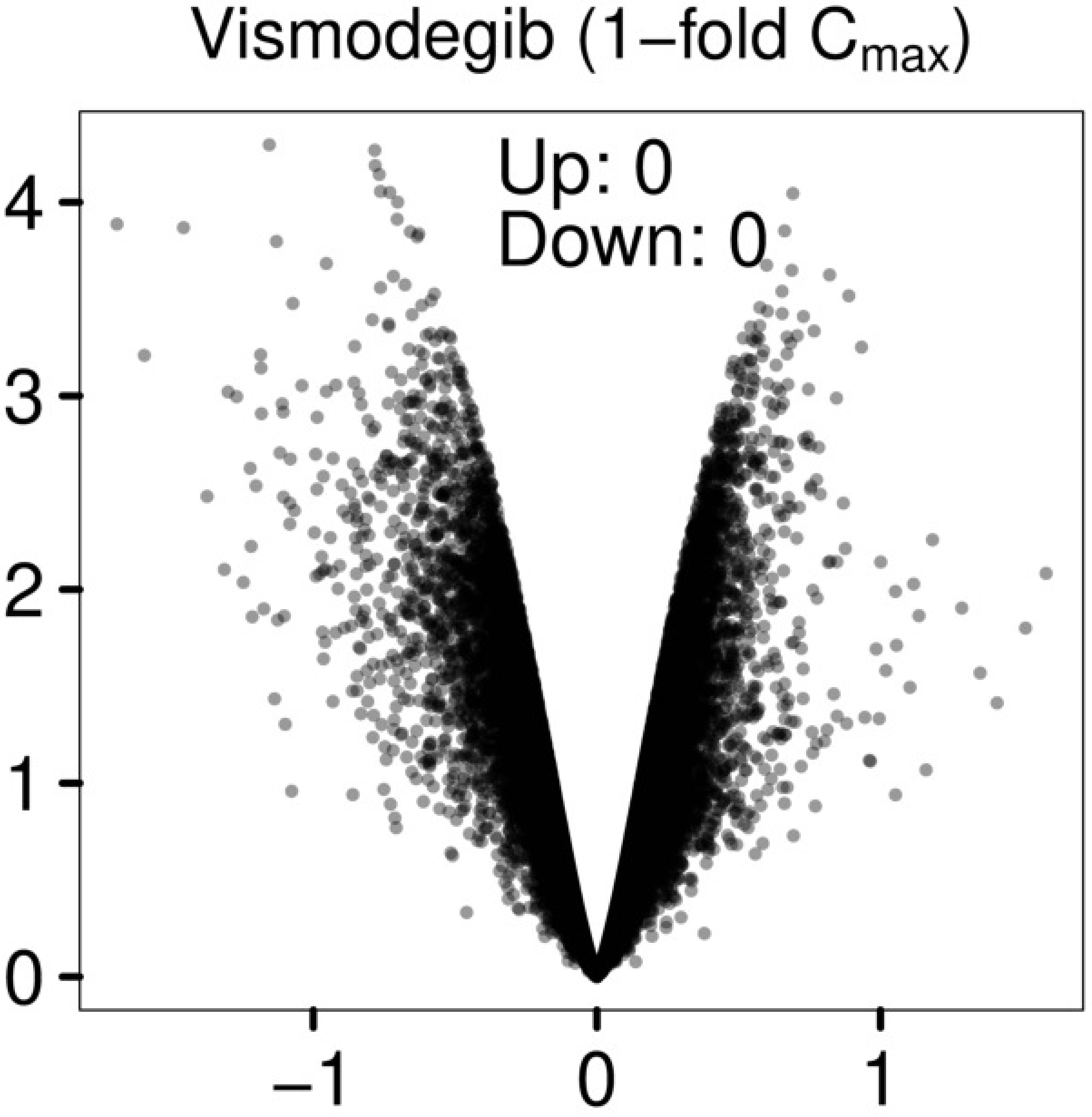
Vismodegib (1–fold Cmax)
Up: 0
Down: 0
4
3
2
1
0
–1
0
1
